# SIRT6 Activation Improves Intervertebral Disc Health in the Aging Spine

**DOI:** 10.1111/acel.70709

**Published:** 2026-10-19

**Authors:** Pranay Ramteke, Bahiyah Watson, Sanjana Jagannath, Sarah E. Bell, Mallory Toci, Olivia Ottone, Qinglin Wu, Oliver Abinader, Ramkrishna Mitra, John A. Collins, Ruteja A. Barve, Makarand V. Risbud

**Affiliations:** ^1^ Department of Orthopedic Surgery Sidney Kimmel Medical College, Thomas Jefferson University Philadelphia Pennsylvania USA; ^2^ Labcorp Morrisville North Carolina USA; ^3^ Division of Biostatistics and Bioinformatics, Department of Pharmacology Physiology, and Cancer Biology, Thomas Jefferson University Philadelphia Pennsylvania USA; ^4^ Center for Translational Bioinformatics, Washington University School of Medicine St. Louis Missouri USA

**Keywords:** autophagy, epigenetic regulation, extracellular matrix remodeling, histone modification, nucleus pulposus, senescence

## Abstract

Aging is a critical risk factor for intervertebral disc degeneration, a major contributor to chronic low back pain. Loss‐of‐function studies showed that SIRT6, a nuclear NAD^+^‐dependent deacetylase, maintains disc health. We therefore investigated whether long‐term SIRT6 activation via systemic delivery of the small‐molecular agonist MDL‐800 improves disc health in 24‐month‐old mice. Studies revealed healthy disc morphology, enhanced cell viability, and lower degeneration scores following MDL‐800 treatment. SIRT6 activation decreased H3K9ac levels, improved extracellular matrix quality, and reduced senescence, as evidenced by lower levels of p21, IL‐6, TGF‐β, and CCL2. Tissue RNA‐Seq, measurements of histone 3 modification, and multi‐omics ATAC‐seq/RNA‐seq revealed altered epigenetic (decreased H3K9ac, H3K36me3, and H3K79me2) and transcriptomic landscapes. MDL‐800 increased autophagy without affecting disc cellular bioenergetics. Plasma metabolomics, scRNA‐sequencing of lymphoid tissues (bone marrow and spleen), and cytokine profiling showed good systemic tolerance of the treatment. While we noted a small decrease in vertebral structural parameters and mild hyperalgesia in treated cohorts, there were no vertebral fractures, and the broader motor function and behavior remained unaffected, raising no safety concerns. Our studies underscore the feasibility of SIRT6 activation as a pharmacological strategy to maintain the health of the aging spine.

## Graphical abstract

Our studies for the first time underscore the feasibility of long‐term SIRT6 activation as a pharmacological strategy to maintain the health of the intervertebral disc in the aging spine.

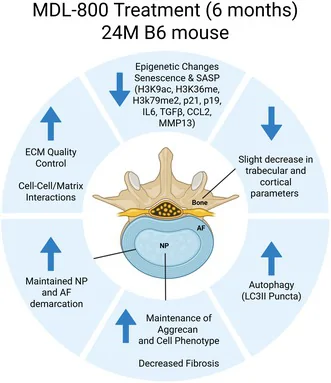

## 1 Introduction

Low back pain (LBP) is the leading cause of global disability, affecting an estimated 619 million individuals in 2020, with projections reaching 843 million by 2050 due to population growth and aging (Ferreira et al. 2023). The peak prevalence of LBP is observed between the ages of 50 and 55, extending into older age groups (Ferreira et al. 2023). One of the primary contributors to LBP is intervertebral disc degeneration and age‐related spinal changes, which lead to structural degeneration, pain, and functional impairments. Disc degeneration is a progressive, multifactorial process involving dysregulated autophagy, cellular senescence, chronic inflammation, and extracellular matrix degradation (Madhu et al. 2021). Current treatments remain symptomatic and often ineffective, underscoring the need for noninvasive, biologically targeted disease‐modifying strategies to reduce the growing global burden of LBP.

Sirtuin 6 (SIRT6) is a nuclear NAD^+^‐dependent enzyme with deacetylase, defatty‐acylase, and mono‐ADP‐ribosyltransferase activities. By removing acetyl groups from histone marks such as H3K9ac, H3K18ac, and H3K56ac, SIRT6 preserves chromatin integrity and coordinates DNA repair, metabolic regulation, and inflammatory responses—processes essential for tissue homeostasis and healthy aging (Kawahara et al. 2009; Korotkov et al. 2021; Tasselli et al. 2016; Yang et al. 2009). Recent findings from human lifespan studies show a prominent role of SIRT6 in aging and age‐associated disorders (Simon et al. 2022; Zhao et al. 2021). In mouse models, loss of SIRT6 accelerates genomic instability, metabolic dysfunction, premature aging, and reduced lifespan, disproportionately affecting males (Mostoslavsky et al. 2006; Peshti et al. 2017). While Kanfi et al. reported a significantly longer lifespan in male SIRT6 transgenic mice through downregulation of IGF‐1 signaling, a later study showed lifespan extension in both male and female mice, albeit with a stronger effect in males than in females via preservation of hepatic glucose output and glucose homeostasis (Kanfi et al. 2012; Roichman et al. 2021). Similarly, a positive impact of SIRT6 on lifespan in other species, mediated by mechanisms including efficient DNA double‐strand break repair and MYC inhibition, has been demonstrated, underscoring SIRT6's role as an epigenetic regulator of genome stability and aging (Taylor et al. 2022; Tian et al. 2019).

SIRT6 plays a prominent role in the musculoskeletal system (Kim et al. 2020; Ramteke et al. 2025). *Sirt6* ^*AcanCreERT2*^ mice (Sirt6 loss) show significantly repressed IGF‐1/Akt signaling that was linked to enhanced injury‐induced and age‐associated osteoarthritis (OA) severity (Collins et al. 2021, 2023). Moreover, these SIRT6‐deficient mouse chondrocytes showed diminished antioxidant capacity, evidenced by reduced peroxiredoxin 1 (Prx1) and increased thioredoxin interacting protein (TXNIP) levels (Collins et al. 2021). Similarly, in another mouse model of SIRT6‐loss in cartilage (*Sirt6* ^*Col2a1CreERT2*^) increased chondrocyte senescence and age‐associated OA severity, and a critical role of SIRT6 in STAT5 deacetylation, inhibiting the pathogenic IL‐15/JAK3/STAT5 axis was noted (Ji et al. 2022). Notably, SIRT6 activation in chondrocytes suppresses age‐related DNA damage (Copp et al. 2023). Loss of SIRT6 in osteoblast lineage cells resulted in reduced osteoprotegrin levels and increased osteoclast activation, leading to osteopenia (Kim et al. 2020). Likewise, osteoblast‐ and osteocyte‐targeted *Sirt6* ^*Dmp1Cre*^ mice exhibited osteopenia due to elevated osteocytic expression of *Sost* and *Fgf23*, as well as increased cell senescence (Aobulikasimu et al. 2023). An inactivating SIRT6 mutation has been linked to perinatal lethality and craniofacial abnormalities in humans (Ferrer et al. 2018), and *Sirt6* deletion in Wnt1‐expressing neural crest cells in mice delays palatal osteogenesis, resulting in a cleft palate (Wang et al. 2025). In hematopoietic cells, SIRT6 promotes the osteoclast differentiation program by increasing *NFAT2* and *BLIMP1* and by suppressing *MAFB* (Park et al. 2016). In the rotator cuff, an age‐related decline in SIRT6 has been postulated to contribute to impaired tendon‐to‐bone healing after injury. Experimental overexpression in rotator cuff and primary tenocytes restored Wnt/β‐catenin signaling and reduced sclerostin expression, improving enthesis repair in aged rats following tenotomy with reconstruction, suggesting therapeutic potential for tendon injuries in older individuals (Moon et al. 2023). These context‐specific roles of SIRT6 underscore its importance in maintaining skeletal tissue homeostasis.

In our recent studies, disc‐targeted Sirt6 deletion in mice disrupted disc homeostasis and increased degenerative features with age. We observed increased H3K9ac, DNA damage, cell senescence, and the senescence‐associated secretory phenotype (SASP), accompanied by decreased cellular autophagy in knockout mice, indicating a causal link between SIRT6 activity and disc health (Ramteke et al. 2025). Similarly, SIRT6 activation has been shown to suppress senescence in an acute traumatic disc injury model (Chen et al. 2018). Whether it will improve disc health in the context of age‐dependent degeneration remains unknown. Preclinically validated SIRT6 activators have been shown to accelerate wound healing (Jiang et al. 2022), mitigate hepatic fibrosis (Hamad et al. 2025; Zhang et al. 2021), and improve muscle performance (Song et al. 2022), demonstrating their utility for in vivo applications. We systemically treated aged B6 mice long‐term with MDL‐800, a well‐studied SIRT6 allosteric activator, and observed a significant improvement in disc health outcomes compared with vehicle‐treated, age‐matched mice. Mechanistically, there were changes in chromatin accessibility, evident by reduced H3K9 acetylation, decreased cell death and senescence, and increased autophagy. Systematically, we observed minimal alterations in plasma cytokine levels and the metabolome, and fewer changes in the immune cell profile, suggesting a noninflammatory immunophenotype. In summary, this study is the first proof‐of‐principle validation of long‐term SIRT6 activation in a naturally aged mouse model, supporting this as a promising biological therapeutic strategy for treating age‐related disc degeneration.

## 2 Results

### 2.1 MDL‐800 Treatment Improves Disc Health in Old Mice Without Adverse Effects on Systemic Metabolism

To investigate whether SIRT6 activation improves disc health in the context of age‐dependent degeneration, we examined the spinal phenotype of B6 mice treated with MDL‐800 for 6 months starting at 18 months of age (Figure 1A). MDL‐800 treatment significantly decreased H3K9 acetylation levels in the disc tissues, confirming increased SIRT6 activity (Figure 1B). When disc degeneration was assessed histologically in the lumbar spine, treatment with MDL‐800 lead to a marked decline in the age‐dependent degenerative features, including NP fibrosis, loss of NP‐AF compartment demarcation, lamellar defects, and clefts indicative of structural disruptions (Figure 1C). Notably, NP cells showed a healthy morphology as evident from preservation of vacuolated cells in the cell band. Modified Thompson grading of MDL‐800‐treated mice showed significantly lower cumulative degeneration scores across the lumbar spine in both the NP and AF compartments compared to vehicle‐treated controls (Figure 1C–E). Notably, level‐by‐level analysis of grading data suggested that L3/4 and L4/5 discs were more responsive to the MDL‐800 treatment than L5/6 and L6/S1 discs, implying that outcomes were likely driven by location‐specific biomechanics (Figure S1). Since cell death is observed during disc degeneration, we performed TUNEL labeling, which showed a significant decrease in TUNEL‐positive cells in *MDL‐800‐treated* mice, but a comparable total cell number, indicating that the elevated apoptosis was not the primary driver of later stages of degeneration (Figure 1F,G). Despite an overall improvement in disc health parameters, MDL‐800‐treated mice did not exhibit altered disc height (DH) or disc height index (DHI) when compared to vehicle‐treated controls (Figure 1H–K).

**FIGURE 1:**
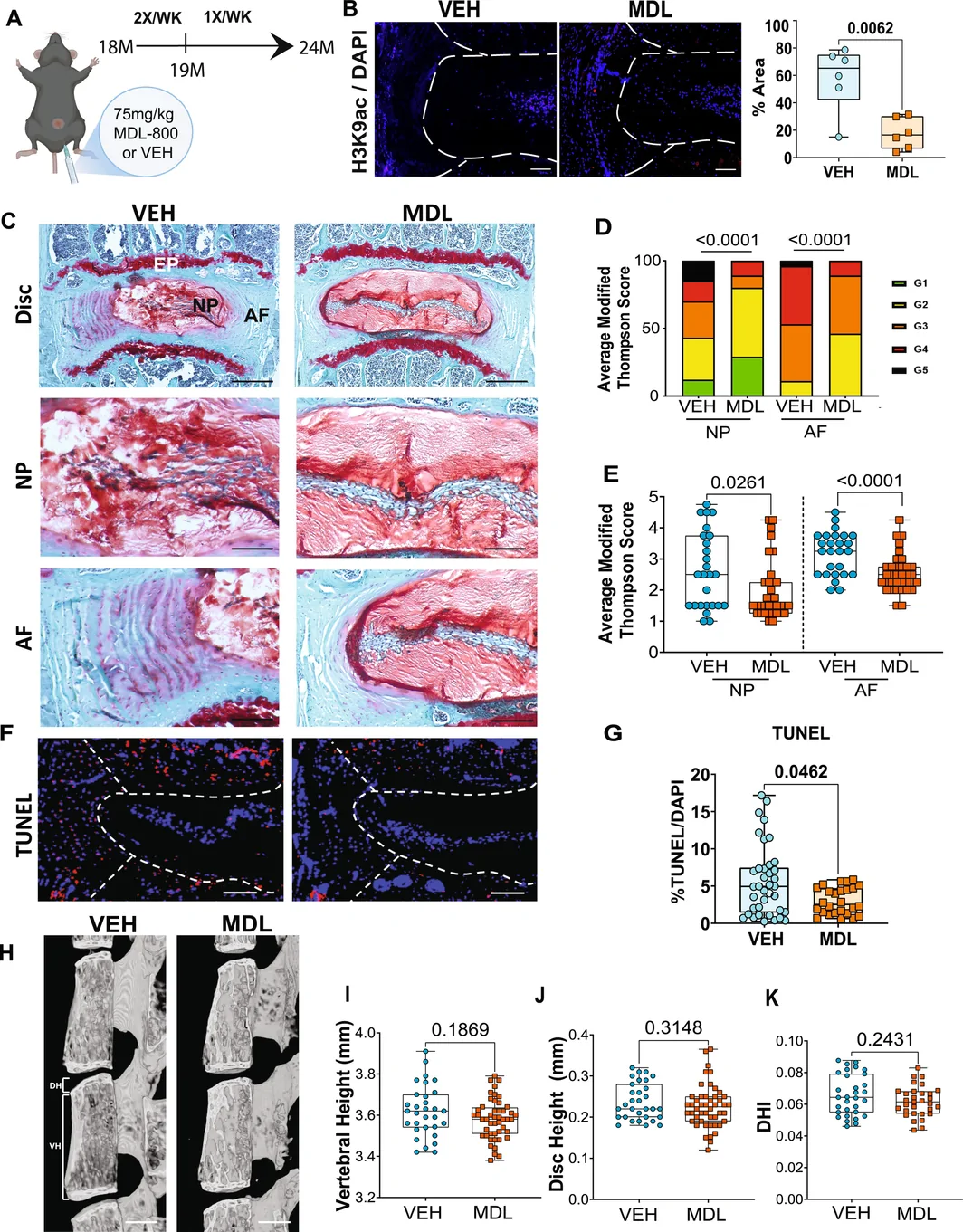
SIRT6 activation by MDL‐800 improves age‐associated intervertebral disc health. (A) Experimental design showing the timeline of MDL‐800 treatment of 18‐month‐old B6 mice. (B) Immunofluorescence staining for H3K9ac shows a significant decrease in staining in the lumbar disc, confirming SIRT6 activation. (C) Safranin‐O/Fast Green staining of lumbar discs of vehicle control (VEH) and MDL‐800 (MDL) treated mice at 24 months. Scale bar row 1 = 200 μm; row 2 = 50 μm. (D, E) Distribution of and average modified Thompson's grades for NP and AF compartments of lumbar discs of VEH and MDL‐800 mice, *n* = 7 vehicle control, and 9 MDL‐800 mice, 2–4 discs/animal. (F) TUNEL staining images and (G) TUNEL quantification, Scale bar = 50 μm. (H–K) μCT analysis showing disc height (DH), vertebral height (VB), and disc height index (DHI) measured at 24 months. *n* = 8–12 mice/group, three discs/animal, scale bar = 1 mm. Quantitative measurements are reported as the median with the interquartile range. Statistical difference between grade distributions (D) was tested using the chi‐square test, and all other quantitative data were compared using an unpaired *t*‐test or Mann–Whitney test, as appropriate.

We then performed plasma metabolic analyses to determine the systemic effects of long‐term MDL‐800 treatment in old mice using LC/MS (Figure S2) and NMR (Figures S3 and S4) assays. LC/MS analysis identified 184 plasma metabolites with high confidence but did not reveal any treatment‐dependent PCA or hierarchical clustering (Figure S2B,C). Out of 6 metabolites that showed significant differences, four metabolites, including palmitoyl sphingomyelin, 5‐aminolevulinic acid, 2‐oxindole, and jasmonic acid, showed a significant increase, while nicotinamide and hypotaurine showed a decrease in MDL‐treated mice (Figure S2D–I). Plasma NMR analysis showed no changes in any of the measured analytes, including indicators of inflammation and metabolic regulation: GlycA, total ketone bodies, acetoacetate, acetone, beta hydroxybutyrate total branched chained amino acids (BCAA), alanine, leucine, isoleucine, valine, citrate, ApoA‐1, ApoB, total triglyceride, total cholesterol, total TRLP, small TRLP, total calibrated low‐density lipoprotein particle (cLDLP), total calibrated high‐density lipoprotein particle (cHDLP), medium LDLP, H4P, TRL size, LDLC, HDLC, HDL size, TRL TG (Figures S3B–M and S4). In conclusion, these plasma analyses revealed minimal metabolic effects of long‐term MDL‐800 treatment in B6 mice, supporting a favorable safety profile under the conditions tested.

### 2.2 MDL‐800 Treatment Preserves NP Cell Phenotype, Aggrecan Levels, and Ameliorates Disc Fibrosis in Aged Mice

As SIRT6 activation improved histological scores of disc health, with a notable effect on NP cell morphology, we determined whether MDL‐800 treatment preserved cell phenotype and matrix constituents using Picrosirius red staining and quantitative immunohistochemistry. Polarized imaging of picrosirius red‐stained disc sections showed diminished presence of collagen fibers in the NP compartment and an increased abundance of small‐diameter fibers and a decrease in thick fibers in the AF of MDL‐800‐treated mice (Figure S5A–D). These findings suggest an overall increase in collagen turnover and amelioration of age‐associated NP fibrosis resulting from sustained SIRT6 activation. To determine the integrity of the disc ECM, we stained the disc sections for Aggrecan (ACAN), a predominant constituent of the NP matrix, and noted a significant increase in abundance (Figure S5E,F). Likewise, NP cells showed increased GLUT1 abundance, a known phenotypic marker, indicating preservation of NP tissue health (Figure S5G,H). There were little changes in the abundance of FCHP, a marker of denatured collagen, and COL1 in the AF (Figure S6A,B). Similarly, both groups exhibited comparable COLX abundance in the NP (Figure S6E), suggesting that SIRT6 activation did not modulate the age‐dependent acquisition of chondrocyte‐like characteristics. These findings suggested that SIRT6 is critical in maintaining a healthy cell phenotype and disc tissue matrix.

### 2.3 SIRT6 Activation Diminishes Senescence and SASP Burden in the Disc

The senescence burden and SASP have been causally linked to age‐dependent disc degeneration (Novais et al. 2021). We therefore investigated the senescence status of MDL‐800‐treated mice. We noted that levels of p19, an upstream regulator of senescence, and p21, a CDK inhibitor important for senescence initiation, were significantly decreased in the discs of MDL‐800‐treated mice (Figure 2A–D) (Stein et al. 1999). Additionally, we determined levels of IL‐6 (Figure 2E,F), TGF‐β (Figure 2G,H), MMP13 (Figure S7A,B), CCL2 (Figure S7C,D), important markers of SASP in the disc (Novais et al. 2021), which were lower in discs of MDL‐800‐treated mice, suggesting an attenuation of the overall burden of SASP and profibrotic factors.

**FIGURE 2:**
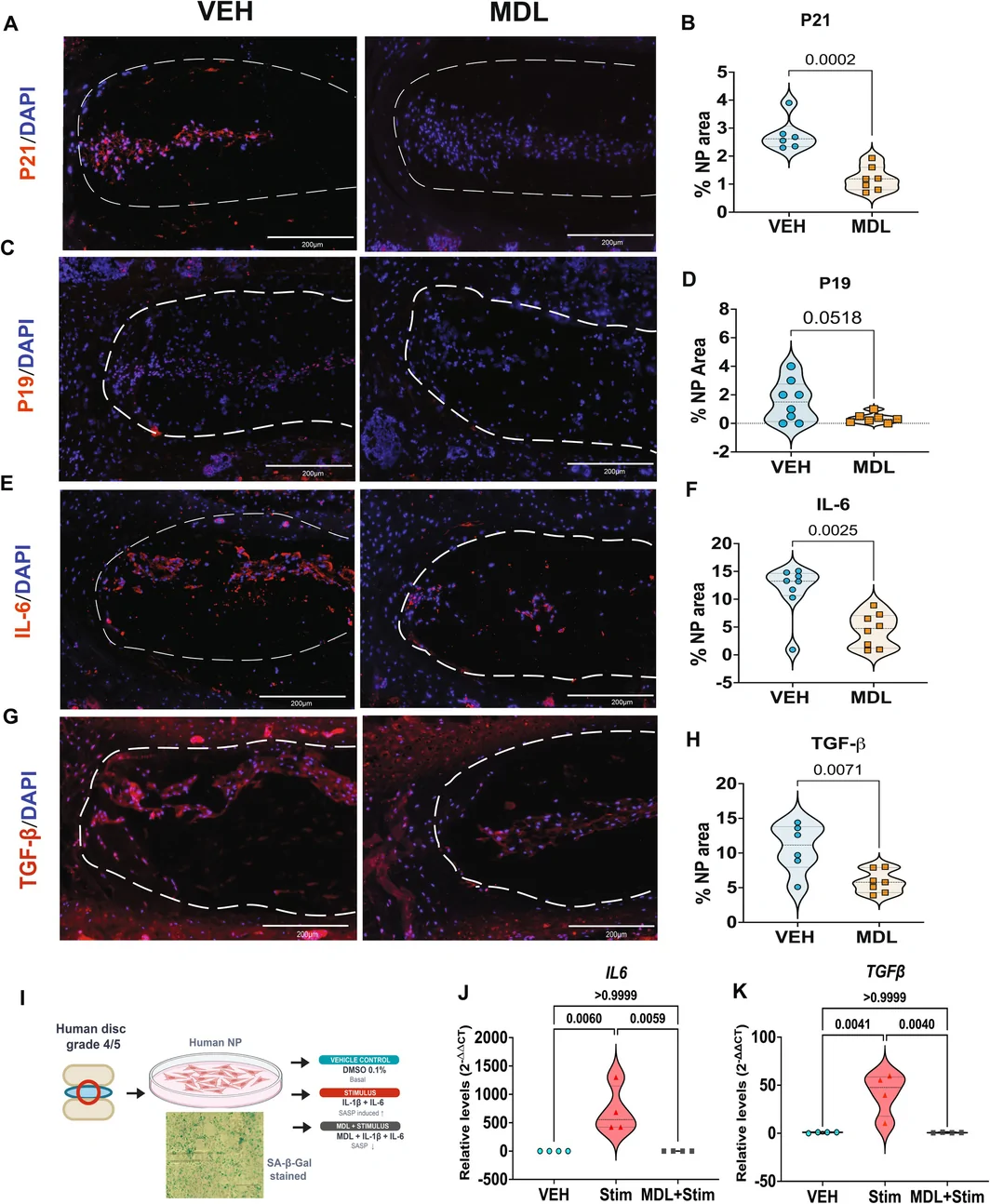
SIRT6 activation decreases senescence and SASP burden in intervertebral discs of MDL‐treated mice and in human NP cells. (A–H) Representative immunofluorescence images and corresponding quantitative analysis of senescence and SASP markers (A, B) p21, (C, D) p19, (E, F) IL‐6, and (G, H) TGF‐β, *n* = 4 animals/group, 2–3 discs/mouse. Scale bar = 200 μm. (I–K) In Grade IV and V human NP cells, MDL‐800 treatment reduces expression of *IL6* (I) and *TGF‐β* (J) to levels comparable to unstimulated levels following inflammatory stimulation. Values are represented as a violin plot showing the median with the interquartile range. Significance was determined using One‐way ANOVA followed by Tukey's multiple comparison test (*n* = 4 biological replicates).

To determine whether the MDL‐800‐mediated decrease in SASP factors observed in mice is reproduced in humans, we conducted a pilot study using NP cells isolated from Grade IV and V degenerated discs from four patients (Figure 2I). The baseline senescence in these cells was high, as evidenced by significant β‐galactosidase positivity. When cells were subjected to a short proinflammatory stimulus of IL‐1β and IL‐6, we observed increases in *IL6* (Figure 2J), *TGFβ* (Figure 2K), *MMP13* (Figure S7E), and *CCL2* (Figure S7F) but not in the *p16* transcript (Figure S7G). Notably, MDL‐800 co‐treatment with stimulus significantly reduced expression of all these markers, with a strong trend toward decreasing *p16* levels.

### 2.4 SIRT6 Activation Drives Changes in the Transcriptomic Profile of NP and AF Tissues

To determine the broader effects of long‐term, sustained SIRT6 activation on the transcriptomic landscape in NP and AF tissues, we performed RNA sequencing. While SIRT6 suppresses gene expression via limiting chromatin accessibility, long‐term effects were expected to reflect second‐order adaptive changes. Indeed, we observed that more genes were upregulated than downregulated in both NP and AF tissues by MDL‐800 treatment (Figure S8). In NP tissue, there were major upregulated thematic clusters related to (1) Histone acetylation/activity, and JNK/MAPK signaling (2) a large supercluster for circadian clock, cell polarity/notochord, follistatin‐inhibin‐activin signaling, connexins/Cx43, and matrix remodeling (3) lipid metabolism and PPAR signaling, and (4) microtubules anchoring at centrosome (Figure 3). Notably, when downregulated DEGs were analyzed, we observed a less prominent thematic clustering with major clustering centered around (1) follistatin‐myostatin and actin‐myosin, (2) connexins/Cx43 and Natriuretic peptide/myocardium (Figure S9). The upregulated DEGs in AF clustered into two major superclusters. The first cluster was related to skeletal tissues, with themes including articular cartilage development, endochondral processes, and collagen/MMPs. While the second supercluster was related to neuronal processes, it encompassed themes such as the hypothalamic nucleus, synaptic vesicle exocytosis, axonal guidance, sensory neurons, synapses, clathrin‐coated vesicles, and voltage‐gated Ca^2+^ channels (Figure S10). Again, compared with the extensive clustering observed in upregulated DEGs in AF, less clustering was observed in downregulated DEGs. Some of the enriched themes in these downregulated DEGs included immune cell function (CD8 effector T cells, granzyme secretion, MCP‐1 Production, PD‐1 secretion, macrophage response), caspase activation, myogenesis, Schwann cells, and oligodendrocytes and myelination (Figure S11).

**FIGURE 3:**
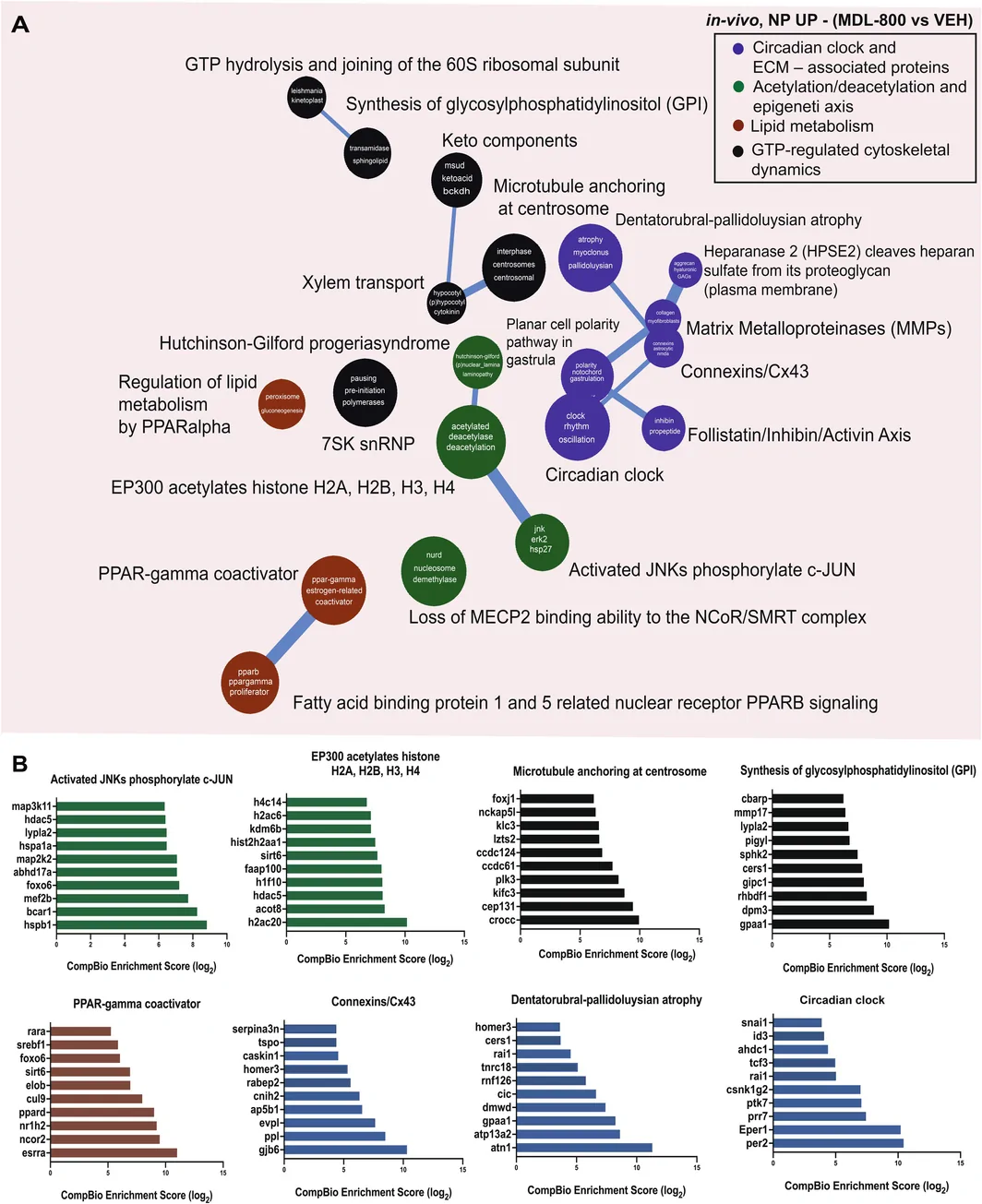
SIRT6 activation alters the transcriptomic landscape of NP tissue. (A) Thematic map of CompBio analysis of Upregulated DEGs (*p* < 0.05, FC > 1.75) from RNA sequencing of NP tissue transcripts from 24‐month‐old, MDL‐800 treated B6 mice compared to VEH‐treated mice, represented as a ball and stick model, *n* = 5 mice/group, 5–6 pooled discs/animal. DEGs were mapped to major themes related to histone acetylation, JNK/MAPK signaling, circadian clock, follistatin/inhibin, connexins, matrix remodeling, lipid metabolism, and microtubules. The enrichment of themes is shown by the size of the ball, and the connectedness between themes is proportional to the thickness of the lines between them. Themes of interest are colored, and superclusters comprised of related themes are highlighted. (B) Top thematic DEGs plotted based on CompBio entity enrichment score.

### 2.5 MDL‐800 Treatment Causes Changes in Immune Cell Profiles in Lymphatic Tissues Without Systemic Inflammation

Immune cells play a crucial role in regulating aging phenotypes (Delgado‐Pulido et al. 2025). To investigate the immune cell profile and its association with disc phenotype in MDL‐800 treated mice, we performed single‐cell RNA sequencing (scRNA‐seq) of two major lymphatic tissues, the spleen and bone marrow (Figure 4, Figures S12 and S13). After normalization, the splenocytes (Figure 4) and bone marrow cells (Figure S12) were grouped into 12 and 15 clusters, respectively, using the FindNeighbors and FindClusters functions in the Seurat single‐cell analysis package. Moreover, the data was visualized according to treatment, and cell populations were identified using the SingleR annotation tool, demonstrating differences in the proportion of B cells, T cells, and granulocytes, and a small change in monocytes or macrophages in the MDL‐800 treated group in lymphoid tissues (Figure 4B–E, Figure S12B–E). Previous studies have implied a relationship between immune cell heterogeneity with functional state and origin (Papalexi and Satija 2018; Sebastian et al. 2022). To further visualize cellular heterogeneity, T cell populations were subdivided into CD4 ^+^, CD8a ^+^, and CD4 ^−^, CD8a ^−^ T cells (Figure S13). We observed an overall decrease in T cell numbers in the MDL‐800‐treated mice (Figure S13A–D,G–J). When cell proportions were compared, there was a relative increase in the proportion of CD4 ^+^ and a decreased proportion of CD8a ^+^, and CD4 ^−^, CD8a ^−^ T cells in the spleen (Figure S13D′). However, there was no appreciable change in the proportion of T cell subpopulations in bone marrow (Figure S13J′).

**FIGURE 4:**
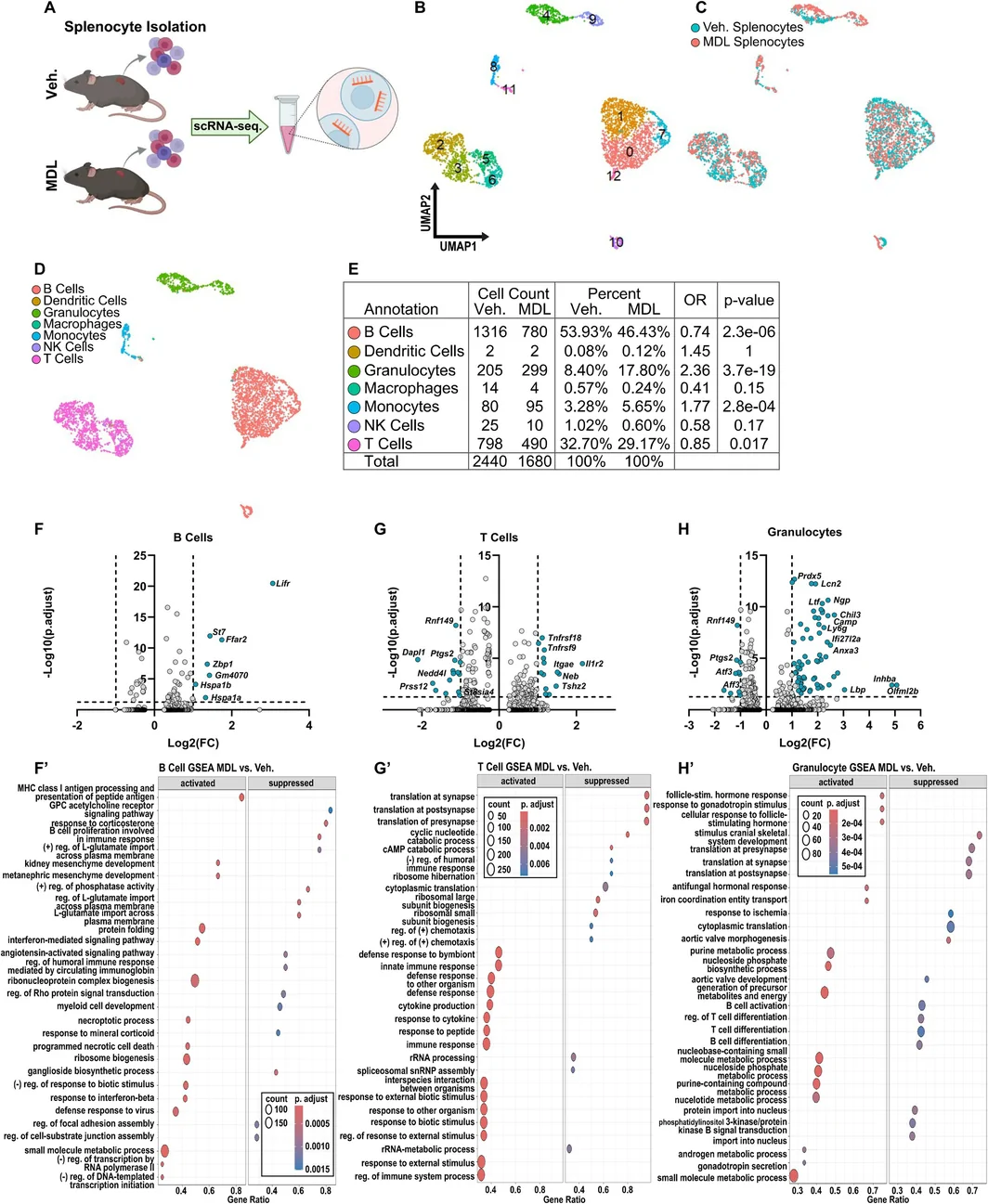
MDL‐treated mice exhibit changes in splenic immune cell profiles. (A) Schematic showing the isolation and scRNA‐sequencing of splenocytes from vehicle‐ and MDL‐800 treated mice. (B) UMAP showing unbiased clustering of splenocytes using Seurat. (C) UMAP with the annotation of Seurat clusters according to treatment. (D) UMAP with the annotation of cells using SingleR. (E) Number and proportion of cells identified in each treatment cohort, with odds ratios and *p*‐values. Volcano plots showing differential gene expression and GSEA in (F, F′) B Cells, (G, G') T Cells, and (H, H′) Granulocytes based on the comparison of MDL‐800 to vehicle‐treated splenocytes.

Gene set enrichment analysis (GSEA) was then performed on DEGs (*p* < 0.05) for cell populations that exhibited significant differences in proportion using the clusterProfiler package (Yu et al. 2012). In the MDL‐800 group, DEGs in splenic B cells showed activation pathways related to MHC Class I processing, plasma membrane protein folding, ribonucleoprotein complex and ribosome biogenesis, and small molecule metabolic process (Figure 4F,F′). In B cells, GSEA identified suppression of the response to corticosterone, B cell proliferation in immune response, plasma membrane L‐glutamate import, and regulation of humoral immune response, with top DEGs including *Lifr*, *St7*, *Ffar2*, *Hspa1a/1b*, responsible for this enrichment (Figure 4F,F′). In the MDL‐800 group, DEGs from splenic T cells showed activation of defense response–related pathways, with top DEGs including *Tnfrsf9/18*, *Itgae*, *Il1r2*, and *Tshz2* (Figure 4G,G′). T cells also exhibited DEGs linked to the suppression of multiple pathways related to protein translation, ribosomes, and rRNA, with top DEGs that included *Dapl1*, *Nedd4l, Prss12*, and *Ptgs2* (Figure 4G,G′). GSEA of granulocytes showed that MDL‐800 treatment led to activation of the reproductive hormone response and nucleoside metabolism, suppression of pathways related to translation, ischemic response, and an overall decrease in B‐ and T‐cell activation and differentiation (Figure 4H,H′). There was no appreciable enrichment of DEGs in GSEA pathways in bone marrow immune cells in MDL‐800 treated mice.

To investigate whether MDL‐800 dependent changes in immune cell profiles in primary lymphatic tissues were correlated with systemic inflammation status, we measured plasma levels of key cytokines and chemokines. We did not observe any significant alterations in levels of IFNγ, IL‐1β, IL‐2, IL‐4, IL‐5, IL‐6, IL‐10, IL‐12/p70, IL‐15, IL‐17A/F, IL‐27/p28/IL‐30, IL‐33, IP‐10, KC/GRO, MCP‐1, MIP‐1α, MIP‐2, and TNF‐α (Figure S14A–R), suggesting an absence of systemic inflammation.

### 2.6 SIRT6 Activation Alters H3K Modifications and Increases Autophagy in NP Cells Without Affecting Cellular Metabolism

To delineate mechanistic insights into how MDL‐800 treatment and subsequent SIRT6 activation affect disc cells, we performed in vitro experiments using primary rat NP cells (Figure 5A). Western blot analysis showed a significant decrease in H3K9ac, a known marker of SIRT6 activation, without changes in SIRT6 levels in MDL‐800 treated NP cells (Figure 5B,C). This finding is further supported by immunofluorescence staining, which shows decreased nuclear H3K9ac staining and comparable levels of H3K27ac in MDL‐800‐treated cells (Figure 5D).

**FIGURE 5:**
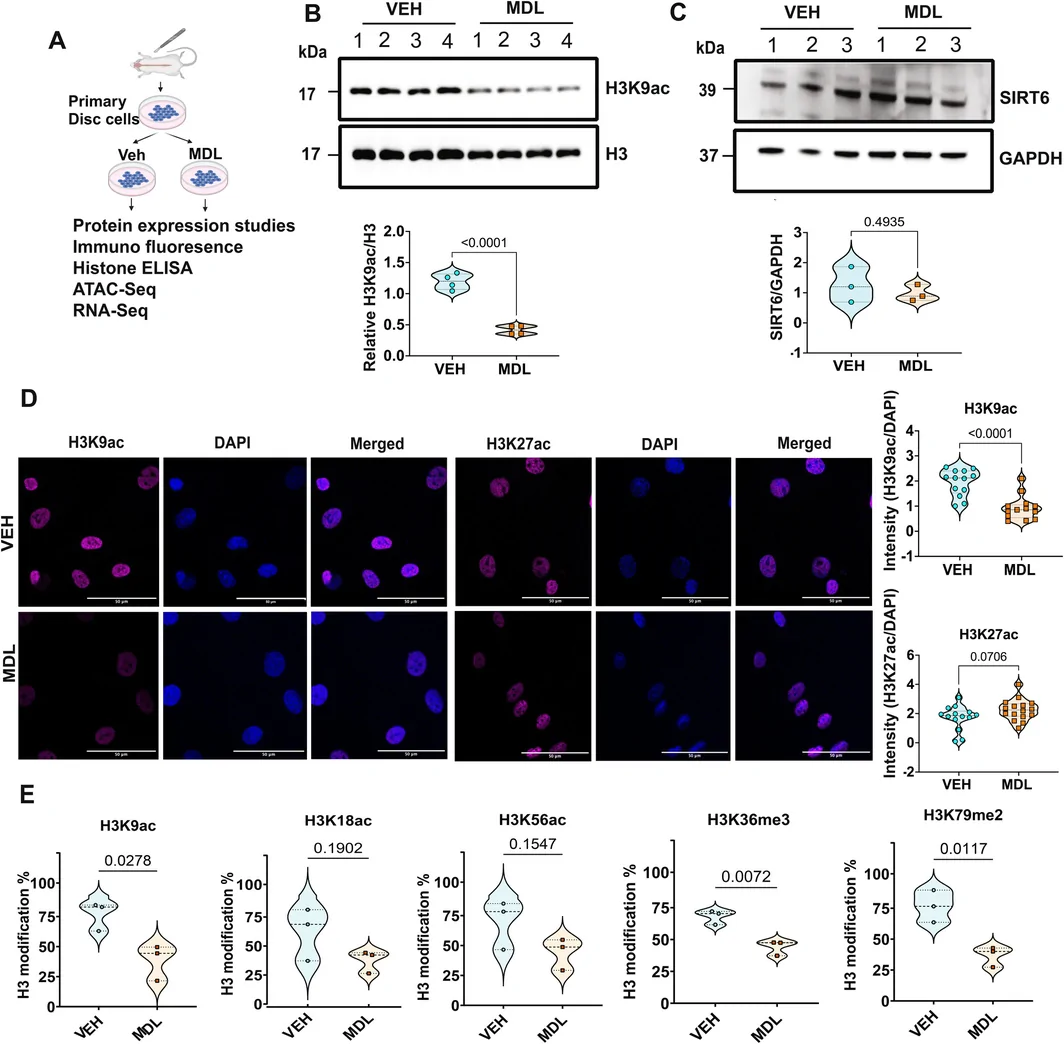
SIRT6 activation alters histone modifications and chromatin accessibility in disc cells. (A) Schematic of in vitro experimental design. (B, C) Western blot analysis of (B) H3K9ac and (C) SIRT6 and corresponding densitometric quantitation normalized to total H3 and GAPDH of VEH and MDL‐800 treated NP cells. *n* = 4 independent cell isolations. (D) Immunofluorescent staining and quantification of H3K9ac and H3K27ac in VEH and MDL‐treated NP cells. (E) Quantitative ELISA for H3 lysine modifications from VEH and MDL‐800 treated NP cells. *n* = 3 independent cell isolations. Statistical significance was determined by an unpaired *t*‐test.

To understand the broader consequences of SIRT6 activation, we measured various H3K modifications using ELISA. We again saw a significant decrease in H3K9 acetylation levels in *MDL‐treated* NP cells (Figure 5E). Moreover, in line with our earlier studies, acetylation status of H3K18 and H3K56 was unaffected (Figure 5E). MDL‐800 treatment also decreased methylation of H3K36me3 and H3K79me2 in NP cells without changes in H3K4me, H3K9me, H3K27me, H3K36me1, H3K36me2, H3K79me1 and H3K79me3 levels (Figure 5E, Figure S15), underscoring tissue‐type specificity in epigenetic changes following elevated SIRT6 action.

SIRT6 modulates autophagy in various tissues, including disc cells (Cheng et al. 2023; Ramteke et al. 2025), and plays a crucial role in maintaining disc health (Madhu et al. 2021). To investigate whether SIRT6 activation induces autophagy, we measured autophagic flux in the presence of MDL‐800 and Bafilomycin A1, a known inhibitor of autophagic flux. Treatment of cells with MDL‐800 resulted in a significant increase in LC3II levels and the LC3II/GAPDH ratio, which showed a trend of further increase in the presence of BafA1, suggesting that MDL promoted autophagic flux (Figure 6A,B). Furthermore, we observed a significant increase in the number of LC3‐positive autophagic puncta in MDL‐800‐treated NP cells (Figure 6C,D). Since SIRT6 is known to modulate glycolytic metabolism, we determined the effect of MDL treatment on the bioenergetics of NP cells using Seahorse assays (Sebastián et al. 2012). MDL‐800‐treated cells showed ATP production rates comparable to those of vehicle‐treated control cells, suggesting no short‐term effect on cellular bioenergetics (Figure 6E–G).

**FIGURE 6:**
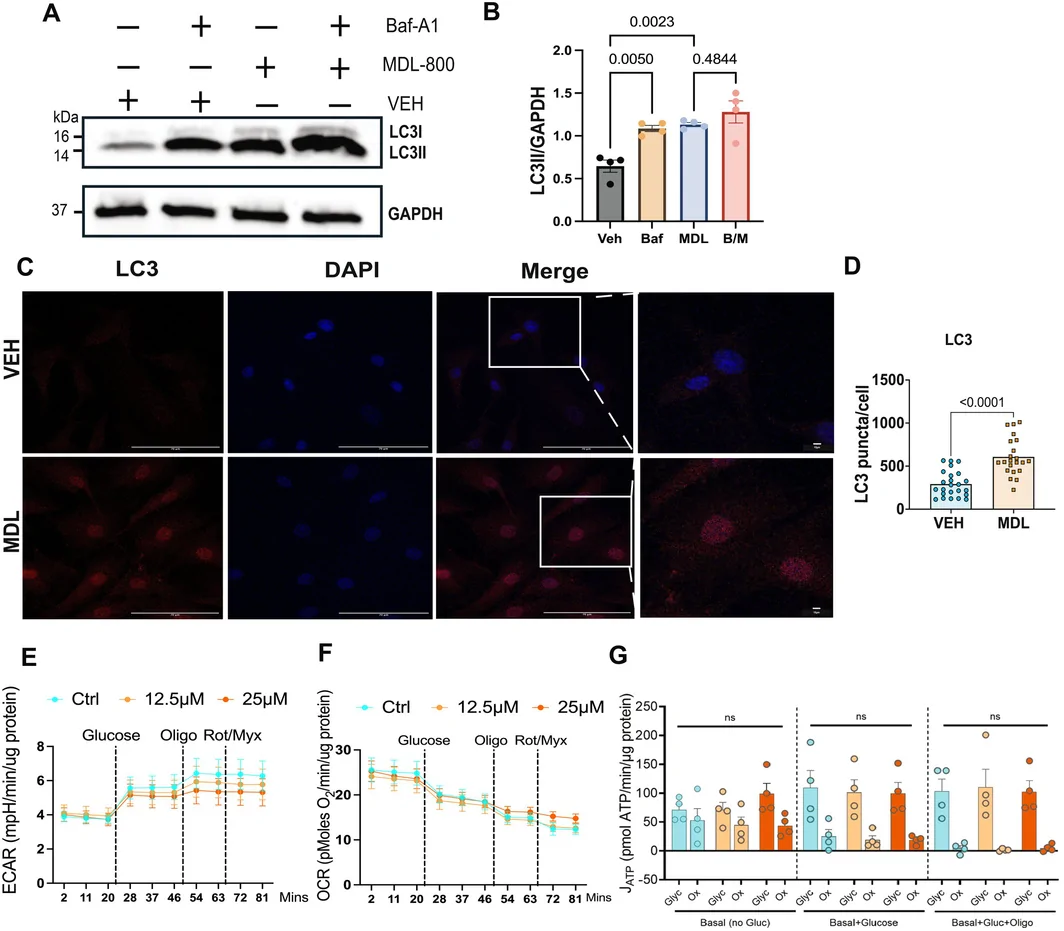
SIRT6 activation promotes NP cell autophagy without altering cellular bioenergetics. (A) Immunoblot of LC3 and (B) corresponding densitometric quantitation of LC3II/GADPH of VEH and MDL‐800 treated NP cells with or without BafA1. *n* = 4 independent cell isolations. (C) Immunofluorescence analysis and (D) quantitation of LC3 puncta in VEH and MDL‐800 treated NP cells cultured in hypoxia. Significance was tested with an unpaired *t*‐test. Scale bar = 70 μ. *n* = 3 independent cell isolations. (E, F) OCR and ECAR traces in VEH versus NP cells treated with MDL‐800 for 24 h were used to determine the (G) ATP production rate. Quantitative data are represented as the mean ± SEM; *n* = 4 independent cell isolations, each experiment performed in quadruplicate. Significance was determined using one‐way ANOVA with Sidak's or Kruskal–Wallis with Dunn's multiple comparison test.

### 2.7 Transcriptomic Changes in MDL‐Treated Primary NP Cells Align With Processes Affected in Mice

We performed RNA‐seq and ATAC‐seq to understand the direct consequence of SIRT6 activation on the transcriptomic landscape of *MDL‐800‐treated NP* cells. CompBio analysis of DEGs from RNAseq (Log_2_FC ≥ 1.5, FDR ≤ 0.05) revealed upregulation of three major interconnected super thematic clusters related to (i) actin‐myosin cytoskeleton and cell junctions, (ii) skeletal tissues, TGFβ/actin/BMP signaling, and extracellular matrix, (iii) glycolysis, purinergic signaling, and aryl hydrocarbon receptor activity (Figure S16). The downregulated clusters in *MDL‐*treated cells included two superclusters with themes related to: (1) fatty acid β‐oxidation, PPAR signaling, mevalonate pathway, dioxygenases and diacylglycerol formation, and (2) Hsp70/Hsc70 chaperons, Jagged1‐Notch1 signaling, Cx36 gap junctions, and primary cilia (Figure S17). These processes have been correlated with disc health (Hiyama et al. 2011; Zhang and Lin 2008). We then used an advanced feature of the CompBio tool, which identifies significantly affected shared pathways between two datasets, to further identify the pathways commonly affected in NP tissue from MDL‐800 treated mice and in primary NP cells in vitro, regardless of the duration of *SIRT6* activation. A significant overlap in upregulated themes was noted between the two datasets for ECM proteins, collagens, ECM turnover, MMPs, myofibroblasts, gap junctions, cell–cell and cell‐matrix adhesion, actin cytoskeleton, microtubules, activin/BMP/FGF, and eNOS signaling (Figure S18A). Within downregulated datasets, themes related to ECM, collagens and fibronectin, chemokine and cytokines (interferon, IL12), coagulation and clotting (fibrinogen, fibrinolysis, and plasmin) were shared (Figure S18B).

Next, using ATAC‐seq data, we identified chromatin accessibility peaks and performed differential chromatin accessibility analysis between the groups. Chromatin accessibility signal profiles for each sample were visualized as heatmaps, and the average relative distribution of ATAC‐seq peaks assigned to genomic features, including promoters, introns, exons, and distal regions, in vehicle control and MDL‐800‐treated NP cells was computed (Figure S19A,B). We identified 251 upregulated (enriched ATAC peaks) and 21 downregulated (diminished ATAC peaks) shared genes between ATAC‐ and RNA‐seq datasets (FC ≥ 2, FDR < 0.05), which are shown in a heatmap (top genes sorted based on ATAC‐seq fold‐changes) and a quadrant plot (Figure S19C,D), demonstrating a strong correlation between chromatin accessibility and gene expression. We performed CompBio analysis on these shared genes and identified enriched and highly connected themes in upregulated genes related to HIF‐1 signaling, Glut1‐dependent glucose import, glycolytic metabolism, actin cytoskeletal organization, chondrocyte morphogenesis‐aggrecan, ECM‐collagens, and MAPK signaling, suggesting favorable transcriptomic outcomes. On the other hand, downregulated genes showed enrichment in themes related to multiple aspects of lipid biology (Figure S20). Additionally, a set of seven genes *Serpine1, Evpl, Kcnh2, Gadd45g, Dlk2, Csrnp1*, and *Ptk7* were identified as consistently upregulated by MDL‐800 treatment in both in vivo and in vitro NP RNA‐seq datasets and shared with ATAC‐seq data. *Serpine1 and Evpl are* shown as average chromatin accessibility maps (Figure S19E). No genes were consistently downregulated across all three datasets.

### 2.8 MDL‐800 Treatment Results in Vertebral Bone Changes and Causes Mild Hyperalgesia Without Mechanical Allodynia, Axial Discomfort, or Ambulation Impairment in Mice

While there were no discernable differences in EP morphology between treatment groups (Figure 1c), in the MDL‐800 treated cohort, we noted a slight decrease in vertebral trabecular bone structural parameters (Figure S21A), including BV/TV, trabecular number (Tb.N), and thickness (Tb. Th), without changes in trabecular spacing (Figure S21B–E), suggesting a minimal effect on bone turnover. *MDL* *‐800 treated* mice also exhibited a slight decrease in cortical bone parameters, including % closed porosity, cortical thickness (Cs. Th); again, notably, without changes in bone perimeter (B.Pm) (Figure S21F–I). Notably higher lumbar bone mineral density, both integral and trabecular, has been correlated with an increased risk of disc height loss and diminished disc area in humans (Harada et al. 1998; Kaiser et al. 2018), suggesting that a small decrease in bone structural parameters could be protective.

To assess if MDL‐800 treatment caused any behavioral changes, mechanical (Von Frey filament) and temperature (Hargreaves thermal; cold plantar assay) sensitization were measured in MDL‐800 and vehicle‐treated mice (Figure S22A) (Novais et al. 2025). While MDL‐800 treatment did not alter the threshold to mechanical stimulation, we observed decreased latencies to cold and hot stimuli compared with vehicle controls (Figure S22B–E), which may arise from the drug's action on peripheral nerves independent of its action on the disc. The open field test, however, showed comparable performance between cohorts, suggesting no adverse effect on overall ambulation in MDL‐800‐treated mice (Figure S22F). Likewise, in both treatment cohorts, we observed a similar response to the grip strength test, an important metric used to assess frailty in humans and to measure axial pain in mice (Figure S22G) (Dudzińska‐Griszek et al. 2017; Millecamps et al. 2012). To further study the locomotion‐related parameters, gait analysis was performed. We did not observe changes in swing‐, stance‐, and stride durations, as well as stride length and frequency, suggesting the absence of impaired mobility or gait asymmetry (Figure S22H–K). Accordingly, while these measurements suggested a small effect on vertebral structural parameters and mild hyperalgesia following long‐term MDL‐800 treatment, the effect sizes were too small to cause vertebral fractures, and overall motor function and animal behavior remained unaffected, raising no safety concerns.

## 3 Discussion

Studies have demonstrated a strong link between SIRT6 activity and organismal aging, with *Sirt6* showing a significant correlation with increased lifespan in humans (Hirvonen et al. 2017; Simon et al. 2022), mice (Kanfi et al. 2012; Roichman et al. 2021), and other long‐lived species (Tian et al. 2019). Notably, SIRT6 plays a prominent role in skeletal health (Collins et al. 2023; Ji et al. 2022). Our previous loss‐of‐function studies have established a direct correlation between SIRT6 and intervertebral disc health in the aging spine (Ramteke et al. 2025). Disc cells reside in an avascular, therefore diffusively limited, hypoxic niche and consequently possess limited regenerative capacity when injured (Silagi et al. 2021). Increased apoptosis and senescence, altered matrix synthesis, and activation or inhibition of catabolic processes involved in ECM turnover play pivotal roles in aging and degenerative conditions affecting the disc (Choi et al. 2018; Novais et al. 2021; Sao and Risbud 2025). While an earlier study showed suppression of cellular senescence in an acute traumatic disc injury model following SIRT6 activation (Chen et al. 2018), whether this approach promotes healthy spinal aging remains unknown. Notably, small‐molecular SIRT6 activators have shown favorable outcomes across various preclinical disease models (Hamad et al. 2025; Jiang et al. 2022; Song et al. 2022; Zhang et al. 2021), underscoring their utility for in vivo interventions. Here, we show, for the first time, that systemic and long‐term treatment of aged mice with MDL‐800, a SIRT6 agonist, significantly improves outcomes in age‐dependent disc degeneration and promotes healthier phenotypes. Mechanistically, SIRT6 activation results in broader tissue‐level changes in histone acetylation, particularly diminished H3K9 acetylation, along with H3K36 and H3K79 methylation. Furthermore, we noted reduced senescence and SASP burden and increased autophagy in disc cells. This study not only establishes a positive link between SIRT6 activity and intervertebral disc health but also identifies mechanisms central to SIRT6 action on the disc.

Although SIRT6 regulates multiple signaling pathways and cellular processes across different tissues, many of its functions are tissue‐type specific. Importantly, the enzymatic activity of SIRT6—a Class III histone deacetylase, long‐chain fatty‐acylase, and mono‐ADP‐ribosyltransferase—depends on the availability of an essential cofactor, such as NAD^+^. Notably, NAD^+^ levels decline significantly with aging (Yang et al. 2022). Therefore, it is logical to conclude that reduced NAD^+^ availability would largely determine its enzymatic activity and functional impact in the aging spine. To overcome this challenge, we chose MDL‐800, a highly selective allosteric activator of SIRT6 that, by directly binding to an allosteric surface site, enhances SIRT6's affinity for both NAD+ and acylated substrates, thereby promoting catalytic efficiency (Huang et al. 2018). In this study, MDL‐800 delivery to the disc and increased SIRT6 activation were evident by decreased H3K9 acetylation in NP cells.

SIRT6 plays a prominent role in the skeletal system (Kim et al. 2020). Particularly, loss of SIRT6 function in the intervertebral disc and knee articular cartilage leads to disc degeneration and OA (Collins et al. 2023; Ramteke et al. 2025). In line with these findings, Thompson grading in MDL‐800‐treated mice showed lower disc degeneration grades, suggesting improved histological outcomes of disc aging. Moreover, SIRT6‐deficient NP cells and articular chondrocytes exhibited diminished antioxidant defense, increased senescence, DNA damage, and cell death (Nagai et al. 2015; Ramteke et al. 2025). Accordingly, decreased TUNEL‐positive cells and higher GLUT1 and ACAN levels, indicating diminished cell death and maintenance of the NP cell phenotype, supported a positive role for SIRT6 in maintaining a healthy disc cell population.

In NP tissues of MDL‐800 treated mice, we observed enrichment of upregulated genes related to histone acetylation/activity, supporting the central role of SIRT6 in epigenetic regulation in disc (Korotkov et al. 2021; Ramteke et al. 2025). Notably, a strong signal in upregulated DEGs was also centered around interconnected themes related to circadian rhythm/clock and ECM homeostasis, which are positively correlated to disc tissue health (Dudek et al. 2023; Wang et al. 2022). Furthermore, in agreement with our previous observation of increased H3K9ac with methylation levels of H3K36 in Sirt6‐deficient disc cells (Ramteke et al. 2025), SIRT6 activation resulted in diminished acetylation of H3K9, along with decreased methylation of H3K36 and H3K79. Additionally, our studies validated that SIRT6 does not regulate the acetylation status of H3K18 and H3K56 in the disc. Notably, studies have shown a positive correlation between cell senescence and H3K36me and H3K79me levels (Leon et al. 2021; Manosalva and González 2010; Suter et al. 2026). Indeed, a significant decrease in p19 and p21 levels, as well as in important pro‐fibrotic SASP molecules IL‐6, TGF‐β, and CCL2, in MDL‐800‐treated mice highlighted that SIRT6 activation counteracts the senescence pathway. Accordingly, these context‐specific roles of SIRT6 and its epigenetic functions underscore its importance in maintaining disc homeostasis during aging. Furthermore, we noted an overlap in enriched themes between the in vivo and in vitro transcriptomic datasets with select upregulated themes related to collagens, ECM turnover, cell–cell and cell‐matrix adhesion, activin/BMP/FGF signaling; processes linked to disc health (Choi et al. 2018; Novais et al. 2021; Sao and Risbud 2025). On the other hand, downregulated shared themes were also linked to ECM, fibronectin, cytokines, and coagulation and clotting (fibrinogen, fibrinolysis, and plasmin). Notably, one of the shared upregulated genes, SERPIN1/PAI‐1, reflects a broader decrease in the coagulation‐related activity and improved phenotypic outcomes in discs of MDL‐treated mice, as elevated fibrinolysis and plasmin activity are linked to the pathogenesis of OA (Wang et al. 2024). Supporting transcriptomic findings related to ECM homeostasis, we observed changes in the proportions of thin and thick collagen fibers in the AF, suggesting increased matrix turnover and a decreased incidence of NP fibrosis. Notably, these observations in concert with decreased SASP further supported the ability of SIRT6 to promote matrix homeostasis in the disc, as elevated TGF‐β and profibrotic myofibroblast activation have been observed in models of Sirt6 deficiency, linking diminished SIRT6 to fibrosis in multiple tissues, including disc (Hamad et al. 2025; Ramteke et al. 2025; Zhang et al. 2021). Our results also align with the information‐theoretic framework of aging, which characterizes aging as a function of epigenetic changes (Lu et al. 2023).

Autophagy is essential for maintaining physiological functions and cell survival in specialized tissue niches such as the disc (Madhu et al. 2021). Notably, one of the commonly dysregulated pathways during disc degeneration is autophagy. Indeed, in disc‐ and cartilage‐targeted *Sirt6* ^cKO^ mice, we observed decreased LC3 levels and a concomitant increase in the abundance of senescence markers (Ramteke et al. 2025). *Notably, in MDL‐800‐treated NP cells, we observed a marked increase in LC3II levels* without affecting cellular bioenergetics, suggesting an important role for SIRT6 in promoting *autophagy* and inhibiting senescence (González‐Gualda et al. 2021). These findings are in line with a report showing that SIRT6 overexpression rescues acute injury‐induced disc degeneration by modulating autophagy and senescence (Chen et al. 2018).

Since MDL‐800 was administered systemically, we investigated whether *Sirt6* activation alters the immune system and vertebrae. Notably, SIRT6 has been shown to modulate mast cell function (Song et al. 2026) and macrophage polarization (Chen et al. 2022) and to suppress NF‐κB‐driven inflammation by enhancing IκBα expression and silencing pro‐inflammatory gene expression (Chen et al. 2022). While we observed a few changes in immune cell populations in the spleen and bone marrow, GSEA suggested reduced activation of B cells and granulocytes. Notably, a recent study showed that B cell‐deficient mice maintain a CD4 T cell compartment that resists immunosenescence and age‐related decline in health (Khan et al. 2026). Accordingly, a decrease in B cell numbers in MDL‐800‐treated mice, activation of defense response–related pathways in T cells, and a decrease in B and T cell activation functionality in granulocytes likely suggest a nonactivated immune cell profile. Importantly, stable plasma cytokine levels and broader metabolomic profiles, along with the lack of alterations in the inflammatory marker GlyA, suggested no adverse effects on the immune system and metabolic health. A small decrease in a select bone structural parameter was noted and may have resulted from SIRT6's ability to promote osteoclast differentiation from hematopoietic precursors, but a relatively smaller stimulatory effect on osteoblast function (Park et al. 2016). However, the absence of adverse events, such as vertebral fractures, the lack of changes in overall ambulation, gait, and axial pain, and the known inverse correlation between lumbar trabecular bone mineral density and the risk of disc degeneration in humans (Harada et al. 1998; Kaiser et al. 2018), suggests good tolerance of the treatment. It must also be acknowledged here that, while MDL‐800 is a validated, specific SIRT6 agonist, it is not approved for clinical application and is used here as a proof‐of‐principle approach. Additionally, secondary effects of MDL‐800 on the disc from systemic factors cannot be ruled out. Notably, this investigation studied only male mice due to the well‐documented, robust effects of SIRT6 on multiple age‐related phenotypes in males; therefore, future work should elucidate the role of SIRT6 activation in the spinal health of females using FDA‐approved compounds.

In summary, our studies demonstrate a positive relationship between SIRT6 and intervertebral disc health in vivo (Graphical abstract). SIRT6 activation, by modulating the tissue‐level epigenetic landscape, inhibiting cell death and senescence, promoting autophagy, maintaining ECM homeostasis, and likely influencing yet unknown systemic factors, contributed to healthier discs in MDL‐800‐treated mice. Modulating SIRT6 activity through pharmacological activation with specific, well‐tolerated agonists offers a noninvasive, translational strategy to ameliorate age‐dependent disc degeneration in patient populations with mild to moderate disease burden, thus preserving disc health in the aging spine.

## 4 Methods

### 4.1 Mouse Studies

Animal studies were approved by the Institutional Animal Care and Use Committee of Thomas Jefferson University following guidelines from the National Institutes of Health Guide for the Care and Use of Laboratory Animals. Eighteen‐month‐old male C57BL/6 mice (NIA colony, Charles River) were housed in groups of 4 per cage and had access to water and food ad libitum. Mice were randomly divided into two groups, and except for 2 doses/week in the first month, received weekly intraperitoneal injections of either DMSO (vehicle control group) or 75 mg/kg of MDL‐800 (SIRT6 activator) in DMSO for 6 months, that is, up to 24 months of age, *n* = 13 control, 15 MDL‐800 mice. We chose to use male mice based on our recent study, which showed enhanced disc degeneration in male C57BL/6J mice following SIRT6 deletion, as well as previous studies demonstrating larger effect sizes on lifespan and protective effects against age‐associated changes in male mice (Ramteke et al. 2025; Roichman et al. 2021).

### 4.2 Behavioral Studies

Mice were acclimated to the behavioral testing room for 1 h before all behavioral tests. Forelimb grip strength to measure the frailty and axial pain, open field test to assess the general locomotion, Von Frey test for mechanical allodynia, Hargreaves thermal and cold plantar assays to measure hyperalgesia were performed (*n* = 7 vehicle control, *n* = 9 MDL mice) (Novais et al. 2025; Ottone et al. 2025). Gate analysis was performed to understand any changes in the ambulation (Novais et al. 2025) (see Supporting Information Methods for details in Data S1).

### 4.3 Histological Studies

Spines were dissected and fixed in 4% paraformaldehyde (PFA) for either 6 h or 48 h, followed by decalcification in 20% EDTA at 4°C before embedding in OCT or paraffin for sectioning. 7 μm midcoronal sections from four lumbar levels (L3‐S1) of each mouse (*n* = 7 vehicle control, *n* = 9 MDL mice; 2–4 discs/mouse) were stained with Safranin‐O/Fast Green/Hematoxylin for histological assessment using a modified Thompson grading scale for NP and AF by at least three blind observers. Similarly, Picrosirius Red staining was performed to characterize collagen fibers in tissue sections. Safranin‐O staining was visualized using an Axio Imager 2 microscope (Carl Zeiss, Germany) using 5×/0.15 N‐Achroplan or 20×/0.5 EC Plan‐Neofluar objectives and Zen2TM software (Carl Zeiss). The heterogeneity of collagen organization was evaluated using a polarizing light microscope, Eclipse LV100 POL (Nikon, Tokyo, Japan), equipped with a 10×/0.25 Pol/WD 7.0 objective and a DS‐Fi2 camera. Images were analyzed using NIS Elements AR 4.50.00 (Nikon, Tokyo, Japan). Under polarized light, as the thickness of collagen fibers increases, their birefringence color changes from green to yellow to orange to red, that is, from shorter to longer wavelengths (Dayan et al. 1989; Montes et al. 1989), allowing quantitative assessment of compositional information in tissue sections. Color threshold levels were maintained constant between all analyzed images.

### 4.4 Immunohistological Analyses

Deparaffinized sections following antigen retrieval or frozen sections were blocked in 5% normal serum in PBS‐T and incubated with antibodies against H3K9Ac (1:50, Sigma, 06‐942), Collagen I (1:100, Abcam ab34710), Collagen X (1:500, Abcam ab58632), Aggrecan (1:300, Abcam ab11570); GLUT1 (1:200, Abcam, ab40084), F‐CHP (1:100, 3‐Helix), p21 (1:200, Novus NB100‐1941), IL‐6 (1:50, Novus NB600‐1131), TGF‐β (1:200, Abcam; ab92486) (*n* = 4–6 mice/group, 2–3 lumbar discs/mouse were analyzed). For mouse antibodies, a MOM kit (Vector Laboratories, BMK‐2202) was used for blocking and primary antibody incubation. Tissue sections were washed and incubated with species‐appropriate Alexa Fluor‐594 conjugated secondary antibodies (Jackson ImmunoResearch, 1:700). TUNEL staining was performed using the In situ cell death detection kit (Roche Diagnostics). Briefly, sections were deparaffinized and permeabilized using Proteinase K (20 μg/mL), and the TUNEL assay was performed according to the manufacturer's protocol. The sections were mounted with ProLong Gold Antifade Mountant with DAPI (Fisher Scientific, P36934), visualized using an Axio Imager 2 microscope with 5×/0.15 N‐Achroplan or 20×/0.5 EC Plan‐Neofluar objectives, and images were captured with an Axiocam MRm monochromatic camera (Carl Zeiss) and Zen2 software (Carl Zeiss AG, Germany). Both caudal and lumbar discs were used for the analysis. Staining area and cell number quantification were performed using the ImageJ software, v1.53e, (http://rsb.info.nih.gov/ij/).

### 4.5 Human NP Cell Culture, Treatments, and Gene Expression Analysis

Human cervical and lumbar NP cells isolated from discs with Pfirrmann scores of IV and V were cultured in Dulbecco's modified Eagle's medium (DMEM) containing 10% FBS and antibiotic‐antimycotic solution and grown at 37°C under 1% O_2_. Cells in passage 2 were divided into three treatment groups. Vehicle (0.1% DMSO), stimulus only: IL‐ β (10 ng/mL) and IL‐6 (70 ng/mL), and MDL‐800 (25 μM, MedKoo #565267) with stimulus for a period of 24 h (Novais et al. 2026). Following treatments, total RNA was isolated as previously described (Novais et al. 2026), and qRT‐PCR was performed using SYBR Green chemistry. Target gene expression was normalized to *Hprt1*.

### 4.6 Disc Tissue RNA‐Sequencing

NP and AF tissues were dissected from vehicle control and MDL‐800‐treated mice (*n* = 5 mice/group). Samples were homogenized, and DNA‐free, total RNA was extracted using the RNeasy Mini kit (Qiagen). RNA with RIN > 7 was used for RNA‐sequencing analysis. Significantly up‐ and downregulated DEGs (FC > 1.5, *p* < 0.05) were analyzed using the GTAC‐CompBio Analysis Tool (https://gtac‐compbio‐ex.wustl.edu). CompBio, a multiomic analysis platform built on an episodic and semantic memory‐based intelligence engine, is used to analyze DEGs. This engine stores PubMed abstracts and full‐text articles as memories, referencing the input DEGs to identify relevant processes and pathways. Conditional probability analysis is used to calculate the statistical enrichment score for biological concepts (processes/pathways) relative to those expected by random sampling. The scores are then normalized for significance empirically over a large, randomized query group. Related concepts derived from DEGs are grouped into broader, higher level themes, which represent overarching biological patterns, such as pathways, processes, cell types, and structures as described previously (Barve et al. 2025; Madhu et al. 2025).

### 4.7 ScRNA‐Seqencing of Splenocytes and Bone Marrow Cells

Spleens were collected from vehicle control and MDL‐treated mice. Splenocytes were dissociated from the tissue matrix by gentle compression through a 100‐μm strainer (StemCell Technologies, 27217). Similarly, bone marrow cells were isolated by gently flushing the femur and humerus. Thereafter, RBC lysis (BioLegend, 420302) was performed, followed by two PBS + 3% FBS washes. ScRNA‐sequencing was performed on freshly isolated cells using the standard 10X protocol (see Supplemental Methods in Data S1). Raw sequencing data were aligned to the mouse reference transcriptome (mm10‐2020‐A) and processed with Cell Ranger Multi version 7.0.1. The count matrix was analyzed using Seurat version 4.3.0. Briefly, data were converted into Seurat Objects using the CreateSeuratObject function, and all cells were analyzed for their unique molecular identifier and mitochondrial gene fractions; cells were included if 200 < *n*feat < 2500, and mitochondrial genes accounted for < 5%. Data were integrated with logarithmic normalization (LogNormalize) and organized according to variation in the data using FindVariableFeatures. Dimensional reduction was performed using principal components analysis, and the FindNeighbors (dims = 1:20) function, followed by the FindClusters function, was used to calculate the number of cell clusters using the Louvain algorithm, with the resolution parameter set to 0.5. DimPlot was then used to visualize the data as UMAP. Cell types for each condition were determined using SingleR, with the MouseRNAseqData reference dataset and main labels. The clusterProfiler package was used to conduct GSEA on DEGs (FDR < 0.05) identified in various tissue and treatment‐based comparisons.

### 4.8 Plasma Cytokine Analysis

Blood was collected by intracardiac puncture in heparin‐coated syringes and centrifuged at 1500 relative centrifugal force (rcf) at 4°C for 15 min to isolate the plasma (*n* = 10 mice/group). Samples were stored at −80°C until analysis. According to the manufacturer's specifications, levels of pro‐inflammatory proteins, cytokines, and TH17 mediators were analyzed using the V‐PLEX Mouse Cytokine 29‐Plex Kit (Meso Scale Diagnostics, Rockville, MD).

### 4.9 Plasma Metabolomics by LC/MS and NMR

Plasma LC/MS analysis was performed as described previously (*n* = 8 vehicle, 7 MDL mice) (Li et al. 2020). Briefly, polar metabolites were extracted from 50 μL plasma samples with 500 μL ice‐cold 80% methanol, and deproteinated supernatants were stored at −80°C prior to analysis. A quality control (QC) sample was generated by pooling equal volumes of all samples after extraction. LC–MS analysis was performed on a Thermo Scientific Q‐Exactive HF‐X mass spectrometer with HESI II probe and Vanquish Horizon UHPLC system. Hydrophilic interaction liquid chromatography (HILIC) was performed at a flow rate of 0.2 mL/min on a ZIC‐pHILIC column (150 × 2.1 mm, 5 μm particle size, EMD Millipore) with a ZIC‐pHILIC guard column (20 × 2.1 mm, EMD Millipore) at 45°C. Solvent A was 20 mM ammonium carbonate, 0.1% ammonium hydroxide, pH 9.2, and solvent B was acetonitrile. The gradient was 85% B for 2 min, 85% B to 20% B over 15 min, 20% B to 85% B over 0.1 min, and 85% B for 8.9 min. The autosampler was held at 4°C. For each analysis, 4 μL of the sample was injected. The following parameters were used for the MS analysis: sheath gas flow rate, 40; auxiliary gas flow rate, 10; sweep gas flow rate, 2; auxiliary gas heater temperature, 350°C; spray voltage, 3.5 kV for positive mode and 3.2 kV for negative mode; capillary temperature, 325°C; and funnel RF level, 40. All samples were analyzed by full MS with polarity switching. The QC sample was analyzed at the start of the sample sequence and after every 8–14 samples. The QC sample was also analyzed by data‐dependent MS/MS with separate runs for positive and negative ion modes. Full MS scans were acquired at 120,000 resolution with an automatic gain control (AGC) target of 1e6, maximum injection time (IT) of 100 ms, and scan range of 65–975 *m*/*z*. Data‐dependent MS/MS scans were acquired for the top 10 highest intensity ions at 15,000 resolution with an AGC target of 5e4, maximum IT of 50 ms, isolation width of 1.0 *m*/*z*, and stepped normalized collision energy (NCE) of 20, 40, 60. Data analysis was performed using Compound Discoverer 3.1 (ThermoFisher Scientific) with separate analyses for positive and negative polarities. Results were manually processed to remove entries with apparent peak misintegrations and correct commonly misannotated metabolites. Positive and negative data sets of identified compounds were merged, and the preferred polarity was selected for compounds identified in both polarities. Compound quantifications were normalized per volume of plasma injected, which was equivalent for all samples. Values from the study were further normalized to the summed area of identified metabolites in each sample. For compounds identified multiple times at different retention times, a single entry was selected with priority given to standards database matches, followed by greater mzCloud match factors and peak areas.

Additionally, compounds shown in Figures S2 and S3 were measured from plasma samples (*n* = 8 vehicle control, 7 MDL mice) at Labcorp (Labcorp, Morrisville, NC). NMR spectra were acquired on a 400 MHz NMR instrument, the Vantera Clinical Analyzer, from EDTA plasma samples as described for the *NMR LipoProfile* test. The data were transferred and processed using the LP4 lipoprotein profile deconvolution algorithm, as reported in the supplemental methods (Jeyarajah et al. 2006; Matyus et al. 2014).

### 4.10 Micro‐CT Analysis

Micro‐CT (μCT) scanning (Bruker SkyScan 1275) was performed on fixed spines using parameters of 50 kV (voltage) and 200 μA (current) at 15 μm resolution as previously described (*n* = 8 vehicle control, 12 MDL mice, 4 lumbar vertebrae/mouse) (Novais et al. 2021; Ramteke et al. 2025). Images were reconstructed using the nRecon program (Version: 1.7.1.0, Bruker) and analysis was performed using CTan (version 1.17.7.2, Bruker). Disc height and vertebral length were measured at three different points equidistant from the center of the bone on the sagittal plane and used to calculate the DHI.

### 4.11 NP Cell Isolation and Treatments

Primary NP cells from adult Sprague Dawley rats (3–6‐month‐old, Charles River) were isolated and cultured in antibiotic‐supplemented complete DMEM containing 5 mM glucose and 10% FBS. To explore the role of SIRT6 in vitro, cells were treated with either DMSO (1:2000) or MDL‐800 (25 μM in DMSO) in 1% hypoxia for 24–96 h.

### 4.12 Seahorse XF Analysis

In brief, NP cells were plated in a 24‐well Seahorse V7‐PS test plate, treated with MDL‐800 or vehicle for 4 days, and placed in hypoxia (1% O_2_) 24 h before the experiment (*n* = 4 independent experiments, four replicates/experiment). On the day of the experiment, cells were washed three times with 700 μL of KRPH (Krebs‐Ringer phosphate HEPES) and incubated with KRPH + BSA for 1 h at 37°C. Seahorse XFe24 flux analyzer (Agilent Technologies) was used to determine maximum glycolytic capacity and ATP production rate using methods reported by Mookerjee et al. (Mookerjee et al. 2016) Experimental design for ATP‐consumption included sequential additions of 10 mM glucose, 1 μM rotenone plus 1 μM myxothiazol, 2 μg/mL oligomycin. To measure glycolytic capacity, sequential additions of 10 mM glucose, 1 μM rotenone plus 1 μM myxothiazol, and 200 μM monensin plus 1 μM FCCP were performed. The normalized traces for oxygen consumption rate (OCR) and extracellular acidification rate (ECAR) were used to calculate experimental parameters.

### 4.13 Immunoblotting

Vehicle control and MDL‐800‐treated (25 μM) NP cells (*n* = 4 independent experiments) were used to extract either histones (Abcam‐ab113476) or total cellular proteins. 10–40 μg of protein was electroblotted to nitrocellulose membranes (Amersham, GE, Burlington, MA, USA). The membranes were blocked and incubated overnight at 4°C with antibodies against H3K9ac (1:1000, 06–942S, Millipore), Histone 3 (1:1000, 9715), SIRT6 (1:1000, D8D12–12,486), LC3 (1:1000, 12,741), GAPDH (1:3000, 5174) from Cell Signaling Technology. Immunolabeling was detected on the Azure 300 system using an ECL reagent (Azure Biosystems, Dublin, CA), and densitometric analysis was performed with ImageJ.

### 4.14 Histone ELISA

The Histone Modification Multiplex Assay kit (ab185910, Abcam) was used to quantify Histone 3 modifications, including lysine acetylation and mono‐, di‐, and tri‐methylation, using histones isolated from NP cells treated with vehicle control and MDL‐800 for 24 h, according to the manufacturer's instructions (*n* = 3 independent experiments).

### 4.15 NP Cell RNA‐ and ATAC‐Sequencing

Following vehicle control and MDL‐800 treatment (25 μM) of rat NP cells for 24 h, total DNA‐free RNA was extracted using RNeasy mini columns (Qiagen) (*n* = 4 independent experiments). The extracted RNA with RIN > 7 was used for RNA sequencing, and DEGs (Log_2_FC ≥ 1.5, FDR ≤ 0.05) were analyzed using the GTAC‐CompBio tool (https://gtac‐compbio‐ex.wustl.edu). For ATAC‐sequencing, vehicle control and MDL‐800‐treated NP cells were trypsinized and collected by centrifugation at 600–800 g for 5 min at 4°C. Cells were resuspended in 500 μL cryopreservation medium containing 50% serum and 10% DMSO and then cryopreserved in 2 mL cryopreservation tubes and shipped to Azenta for sequencing. Briefly, cells were thawed and treated with Tn5 transposase, and tagmented DNA fragments were purified using a DNA purification kit for library synthesis (Azenta). Purified sequencing libraries were used for paired‐end 150‐bp‐long reads on Illumina NovaSeq. Reads were mapped to the reference genome with default parameters. Aligned ATAC‐seq reads were used to remove duplicates, and peak calling was performed, followed by a multi‐omic analysis of ATAC‐seq and RNA‐seq (FC ≥ 2, FDR ≤ 0.05) (see Supplemental Methods in Data S1).

### 4.16 Statistics

All statistical analyses were performed using Prism7 or above (GraphPad, La Jolla). Because interactions among genetic, biological, and biomechanical factors at individual spinal levels can result in distinct phenotypic outcomes, each intervertebral disc or vertebra was treated as an independent experimental sample, consistent with methodologies adopted by several other groups (Battié et al. 2008; Mohanty et al. 2019; Novais et al. 2021; Tessier et al. 2020). Data are represented as box and whisker plots with median, minimum, and maximum values or mean ± SEM. Data distribution was assessed using normality tests, and differences between the two groups were analyzed using an unpaired *t*‐test. The differences among the three groups were analyzed using ANOVA or the Kruskal–Wallis test for non‐normally distributed data. A chi‐square (χ^2^) or Fisher test, as appropriate, was used to analyze the differences between the distribution of percentages. *p* ≤ 0.05 was considered statistically significant.

## Author Contributions

M.V.R. and J.A.C. conceptualized the study, P.R. and M.V.R. designed the experiments. P.R., B.W., S.J., S.E.B., M.T. and Q.W. performed the experiments, collected, and analyzed the data. O.O., R.A.B., O.A., and R.M. performed bioinformatics analysis. P.R. and M.V.R. interpreted the results, and M.V.R. wrote the original draft of the manuscript. All authors reviewed and approved the final draft of the manuscript.

## Funding

This work was supported by National Institute on Aging, R01AG073349, Michael Michelson Gift Fund, and National Institute of Arthritis and Musculoskeletal and Skin Diseases, R01AR055655.

## Disclosure

The authors have nothing to report.

## Ethics Statement

All animal experiments were performed in accordance with protocols approved by the Thomas Jefferson University IACUC.

## Conflicts of Interest

Q.W. is an employee of Labcorp. The authors declare no conflicts of interest.

## Supporting information


**Figure S1:** Level‐by‐level Thompson grading scores of NP and AF compartments. Grading scores at spinal levels L3/4, L4/5, L5/6, and L6/S1 suggest that genetic, biological, and biomechanical factors at individual spinal levels result in distinct phenotypic outcomes (*n* = 7 mice/group). Data are presented as median with interquartile range, and groups are compared using an unpaired *t*‐test or Mann–Whitney test, as appropriate.
**Figure S2:** LC/MS analysis shows minimal changes in plasma metabolome in MDL treated mice. (A) Schematic of experimental design for plasma metabolomic analysis by LC/MS. (B) PCA plot shows no distinct clustering of samples based on the treatment. (C) Heatmaps of metabolite levels in individual mouse plasma samples and QC controls showing hierarchical clustering of samples. Only six metabolites, (D) palmitoyl sphingomyelin, (E) 5‐aminolevulinic acid, (F) 2‐oxindole, (G) jasmonic acid, (H) nicotinamide, and (I) hypotaurine, showed differential levels between the vehicle and MDL‐800 treatment groups. *n* = 8 vehicle control, and 7 MDL‐800 mice. Significance was determined using an unpaired test or Mann–Whitney test, as appropriate.
**Figure S3:** NMR analysis shows no alterations in plasma metabolic parameters following MDL‐800 treatment. (A) Schematic of experimental design for plasma analysis using NMR. Levels of (B) GlycA, (C) Total ketone bodies, (D) Acetoacetate, (E) Acetone, (F) Beta hydroxybutyrate (G) Total branched chained amino acids (BCAA) (H) Alanine, (I) Leucine, (J) Isoleucine, (K) Valine, (L) Citrate, (M) ApoA‐1, showed no changes with MDL treatment, *n* = 8 vehicle control, and 7 MDL‐800 mice. Significance was determined using an unpaired test or Mann–Whitney test, as appropriate.
**Figure S4:** NMR analysis shows stable plasma lipid profile in MDL‐800 treated mice. Plasma NMR analysis showed no difference in levels of (A) ApoB, (B) total triglyceride, (C) total cholesterol, (D) Total TRLP (E) Small TRLP, (F) total calibrated low‐density lipoprotein particle (cLDLP), (G) total calibrated high‐density lipoprotein particle (cHDLP), (H) medium LDLP, (I) H4P, (J) TRL size, (K) LDLC, (L) HDLC, (M). HDL size, (N) TRL TG between vehicle‐ and MDL‐800 treated mice, *n* = 8 vehicle control, and 7 MDL‐800 mice. Significance was determined using an unpaired test or Mann–Whitney test, as appropriate.
**Figure S5:** SIRT6 activation promotes ECM homeostasis and decreases NP tissue fibrosis. (A) Representative bright field and (B) polarized images of Picrosirius Red stained lumbar disc sections and (C) quantification of collagen fibers from VEH and. MDL‐800 treated mouse discs, *n* = 7 vehicle control, and 9 MDL‐800 mice; 2–4 discs/mouse. Scale bar = 200 μm. (D) Quantification of NP compartment fibrosis using polarized images. (E, F) Representative immunofluorescence images of lumbar disc sections and (G, H) corresponding quantification for Aggrecan (ACAN) and GLUT1. Scale bar = 50 μm, *n* = 6 mice/group and 2–4 discs/animal. White dotted lines demarcate disc compartments. Quantitative measurements are reported as the median with the interquartile range, and significance was determined by using an unpaired *t*‐test or Mann–Whitney test, as appropriate.
**Figure S6:** SIRT6 activation does not alter the overall abundance of Collagen I and X in the disc. (A–F) Representative immunofluorescence images of lumbar disc sections and respective quantitation for (A, B) FCHP, (C, D) COL1, and (E, F) COLX. in 24‐month‐old vehicle‐ and MDL‐800 treated mice, scale bar = 50 μm. *n* = 6 mice/group, 2–4 discs/animal. White dotted lines demarcate disc compartments. Significance was determined using an unpaired *t*‐test.
**Figure S7:** SIRT6 activation suppresses senescence of intervertebral disc cells. (A. D) Representative immunofluorescence images and corresponding quantitative analysis of (A, B) MMP13 and (C, D) CCL2. *n* = 4 animals/group, three discs/mouse. Scale bar = 200 μm. (E–G) Gene expression of senescence marker (E) MMP‐13, (F) CCL‐2 and (G) p16 from Grade IV and V human NP cells following stimulation (Stim) with proinflammatory cytokines IL‐1b and IL‐6 and MDL‐800 co‐treatment with stimulation (MDL + Stim). Values are represented as mean ± SD. Significance was determined using One‐way ANOVA followed by Tukey's multiple comparison test (*n* = 4 biological replicates).
**Figure S8:** SIRT6 activation results in changes in the transcriptomic landscape of disc tissue. RNA‐sequencing of NP and AF tissue transcripts from vehicle and MDL. Eight hundred treated 24‐month‐old mice represented as (A, B) Heat map and hierarchical clustering of *Z*‐score of the top differentially expressed genes (DEGs) from (A) NP and (B) AF tissues from MDL‐800 and vehicle‐treated mice (FC ≥ 1.75, *p* ≤ 0.05). (C, D) Volcano plot of DEGs in the (C) NP and (D) AF showing *p*‐value versus magnitude of change (fold change), *n* = 5 mice/group.
**Figure S9:** SIRT6 activation results in changes in NP tissue gene expression. (A) CompBio analysis of Downregulated DEGs (FC > 1.75, *p* < 0.05) in NP tissue of 24 month‐old MDL‐800 treated B6 mice represented in a ball and stick model, *n* = 5 mice/group, 5–6 pooled discs/animal. The enrichment of themes is shown by the size of the ball, and connectedness is shown based on the thickness of the lines between them. Themes of interest are colored, and superclusters comprised of related themes are highlighted. (B) Top thematic DEGs plotted based on CompBio entity enrichment score.
**Figure S10:** SIRT6 activation results in transcriptomic changes in AF tissue. (A). CompBio analysis of Upregulated DEGs (FC > 1.75, *p* < 0.05) in AF tissue of 24‐month old MDL‐800 treated B6 mice represented in a ball and stick model, *n* = 5 mice/group, 5–6 pooled discs/animal. The enrichment of themes is shown by the size of the ball, and connectedness is shown based on the thickness of the lines between them. Themes of interest are colored, and superclusters comprised of related themes are highlighted. (B) Top thematic DEGs plotted based on CompBio entity enrichment score.
**Figure S11:** SIRT6 activation results in gene expression changes in AF tissue. (A) CompBio analysis of downregulated DEGs (FC > 1.75, *p* < 0.05) in AF tissue of 24 month‐old MDL‐800 treated B6 mice represented in a ball and stick model, *n* = 5 mice/group, 5–6 pooled discs/animal. The enrichment of themes is shown by the size of the ball, and connectedness is shown based on thickness of the lines between them. Themes of interest are colored, and superclusters comprised of related themes are highlighted. (B) Top thematic DEGs plotted based on CompBio entity enrichment score.
**Figure S12:** MDL‐800 treated mice exhibit changes in bone marrow immune cell profiles. (A) Schematic showing the isolation and scRNA‐sequencing of bone marrow cells from vehicle‐ and MDL‐800 treated mice. (B) UMAP showing unbiased clustering of bone marrow cells using Seurat. (C) UMAP with the annotation of Seurat clusters according to treatment. (D) UMAP with the annotation of cells using SingleR. (E) Number and proportion of cells identified in each treatment cohort, with odds ratios and *p*‐values. Volcano plots showing differential gene expression in (F) Monocytes and (G) Granulocytes.
**Figure S13:** MDL‐800 treated mice exhibit changes in T cell profiles. (A) UMAP showing splenic T cells, labeled according to treatment cohort. (B) CD4^+^ T cells and (C) CD8a^+^ T cells within the splenic T cell clusters. (D, D′) The number and proportion of CD4^+^ and CD8a^+^ T cells, with odds ratio and *p*‐value. Volcano plots showing differential gene expression in (E) CD4^+^ T cells and (F) CD8a^+^ T cells. (G) UMAP showing bone marrow T cells, labeled according to treatment cohort. (H) CD4^+^ T cells and (I) CD8a^+^ T cells within the bone marrow‐derived T cell clusters. (J, J') The number and proportion of CD4^+^ and CD8a^+^ T cells, with odds ratio and *p*‐value.
**Figure S14:** Plasma analysis of MDL‐800 treated mice does not show increased systemic inflammation. Multiplex analysis showed no significant changes in the plasma concentrations of (A) IFN‐g, (B) IL‐1b, (C) IL‐2, (D) IL‐4, (E) IL‐5, (F) IL‐6, (G). IL‐10, (H) IL‐12/p70, (I) IL‐15, (J) IL‐17A/F, (K) IL‐27/p28/IL‐30, (L) IL‐33, (M) IP‐10, (N) KC/GRO, (O) MCP‐1, (P) MIP‐1α, (Q) MIP‐2, (R) TNF‐α in MDL‐800 treated mice compared to vehicle‐treated mice (*n* = 10 mice/group). Significance was determined using an unpaired *t*‐test or Mann–Whitney test, as appropriate.
**Figure S15:** Effect of MDL‐800 treatment on H3 acetylation and methylation marks. (A–M) Quantitative ELISA showing histone three modifications in VEH and MDL‐800 treated NP cells, *n* = 3 independent experiments. Significance for quantitative measures was determined by using an unpaired *t*‐test.
**Figure S16:** SIRT6 activation in NP cells results in clustering of upregulated DEGs in themes related to the cytoskeleton, cell junctions, skeletal tissues, extracellular matrix, and purinergic signaling. (A) CompBio analysis generated a. thematic map of upregulated DEGs from an RNA sequencing experiment (Log2FC > 1.5, FDR < 0.05) comparing MDL‐800‐treated versus VEH‐treated NP cells, represented as a ball‐and‐stick model. Themes of interest are colored, and superclusters comprised of related themes are highlighted. (B) Top thematic DEGs plotted based on CompBio entity enrichment score. *n* = 4 independent experiments.
**Figure S17:** SIRT6 activation in NP cells results in clustering of downregulated. DEGs in themes related to fatty acid β‐oxidation, PPAR signaling, and Hsp70/Hsc70 chaperons and Jagged1‐Notch1 signaling. (A) CompBio analysis generated a thematic map of downregulated DEGs (Log2FC > 1.5, FDR < 0.05) from the RNA sequencing experiment of MDL‐800‐treated versus VEH‐treated NP cells, represented as a ball‐and‐stick model. Themes of interest are colored, and superclusters comprised of related themes are highlighted. (B) Top thematic DEGs plotted based on CompBio entity enrichment score. *n* = 4 independent experiments.
**Figure S18:** DEGs in NP tissue from MDL‐800‐treated mice share the thematic commonality with NP cells treated with MDL‐800 in vitro. Territorial maps of CompBio Assertion engine analysis showing common themes derived from (A) upregulated and (B) downregulated DEGs from NP tissues of MDL‐800 treated mice and MDL‐800 treated cultured NP cells.
**Figure S19:** Multiomics analysis of ATACseq and RNAseq of MDL‐800‐treated NP cells shows a good overlap in DEGs. (A) Heatmaps showing chromatin accessibility signal profiles in vehicle‐ and MDL‐800‐treated samples, average signal profiles are shown above the heatmaps. *n* = 3 independent experiments. (B) Stacked bars showing the average relative distribution of ATAC‐seq peaks in vehicle‐ and MDL‐800 treated NP cells, annotated to genomic features including promoters, introns, exons, and distal regions. *n* = 3 independent experiments. (C) Heatmap of top 50 up and 21 down DEGs shared between RNA‐seq and ATAC‐seq (FC ≥ 2, FDR < 0.05), sorted based on ATAC‐seq fold‐change intensity. (D) Quadrant map of commonly upregulated and downregulated DEGs between ATAC‐seq and RNA‐seq of MDL‐800 treated NP cells (FC ≥ 2, FDR < 0.05), mapped to one of the thematic superclusters in RNA‐Seq data shown in Figure 8 and Figure S13. (E) Gene tracks showing enriched peaks for Serpine1 and Evp1 shared across ATAC‐seq and RNA‐seq (in vitro and in vivo) analyses. ATACseq *n* = 3 experiments, in vitro RNAseq *n* = 4 independent experiments, in vivo RNAseq *n* = 5 mice/group.
**Figure S20:** CompBio analysis of unique DEGs shared between RNAseq and ATACseq datasets of NP cells treated with MDL‐800. Thematic map of unique DEGs shared between RNAseq and ATACseq (Log2FC > 1.5, FDR < 0.05) from MDL 800‐treated versus VEH‐treated NP cells, represented as a ball‐and‐stick model. (A) Map of upregulated DEGs and (B) downregulated DEGs.
**Figure S21:** MDL‐800 treatment in B6 mice affects vertebral bone structural parameters. Representative μCT reconstructions showing (A) transverse section through the lumbar vertebra of 24‐month‐old VEH and MDL‐treated mice. (B–E) Trabecular bone properties, (B) BV/TV, (C) Tb. N. (trabecular number), (D) Tb. Th. (trabecular thickness), (E) Trab. Sp. (trabecular separation) (F–I) Cortical bone properties showing (F) closed porosity (%), (G) Cs. Th. (cross‐sectional thickness), (H) Cs. Ar. (cross‐sectional bone area) and (I) Cs. Pm. (cross‐sectional perimeter) are shown, *n* = 8 vehicle control, and 12 MDL‐800 mice, four vertebrae/mouse analyzed. Statistical differences between groups were tested using an unpaired *t*‐test; *p* < 0.05.
**Figure S22:** Long‐term MDL treatment has minimal effects on behaviors associated with pain and ambulation in old B6 mice. (A) Schematic of the experimental setup of the behavioral assays. Sensitization analysis: (B, C) mechanical (Von Frey), left and right paw, (D) hot (Hargreaves), and (E) cold stimulus. Ambulation, frailty, and axial pain measurements: (F) Open field (G) Grip strength and Gait analysis parameters. (H) Swing duration (I) Stance duration, (J) Stride duration (K) stride length, (L) swing time, (M) Stride frequency, *n* = 7 vehicle control, *n* = 9 MDL‐800 mice. Statistical difference between groups was tested using an unpaired *t*‐test or a Mann–Whitney test, as appropriate.

**Data S1:** Supporting Information material.

## Data Availability

Sequencing data associated with this study are openly available in Gene Expression Omnibus (GEO) repository at https://www.ncbi.nlm.nih.gov/gds, reference number GSE320197, GSE320429, GSE337792, GSE337900. All datasets generated and analyzed during this study are included in this published article.
