# Supplementary material for: SIRT6 Activation Improves Intervertebral Disc Health in the Aging Spine
Source: Aging Cell. 2026 Oct 19;25(10):e70709. doi: 10.1111/acel.70709 (PMC13679769; doi:10.1111/acel.70709)

Supplemental material

First Probing - H3K9ac
Stripped, second probing-H3

A Original image for 6B

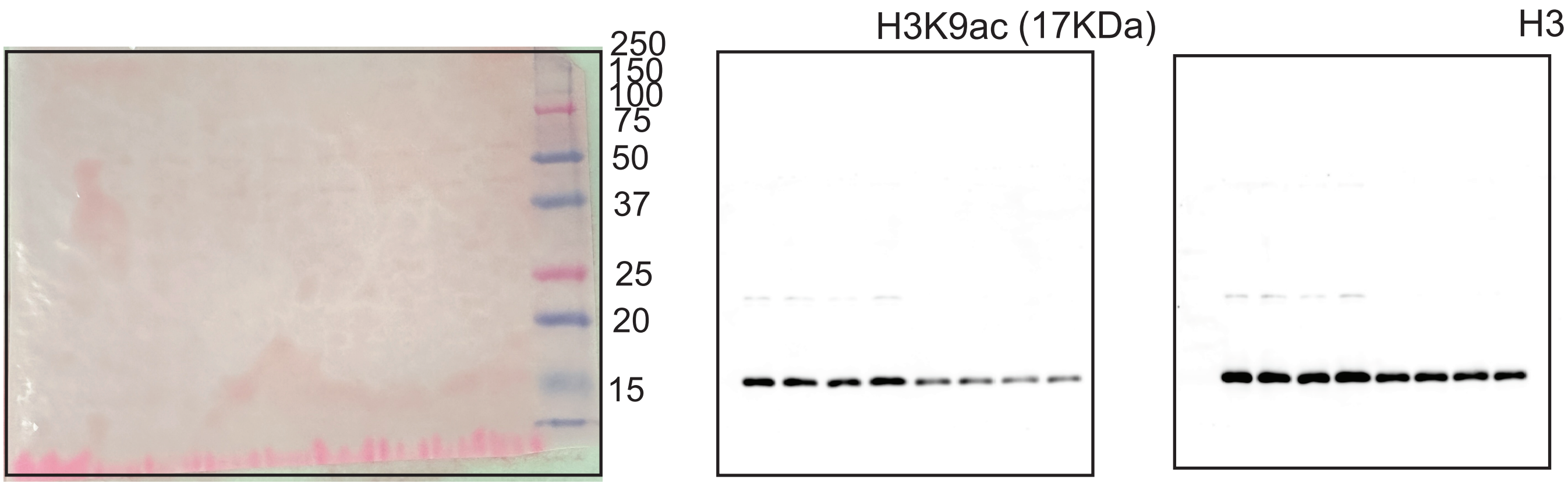

B Original image for 6C

First Probing - SIRT6
Stripped, second probing-GAPDH

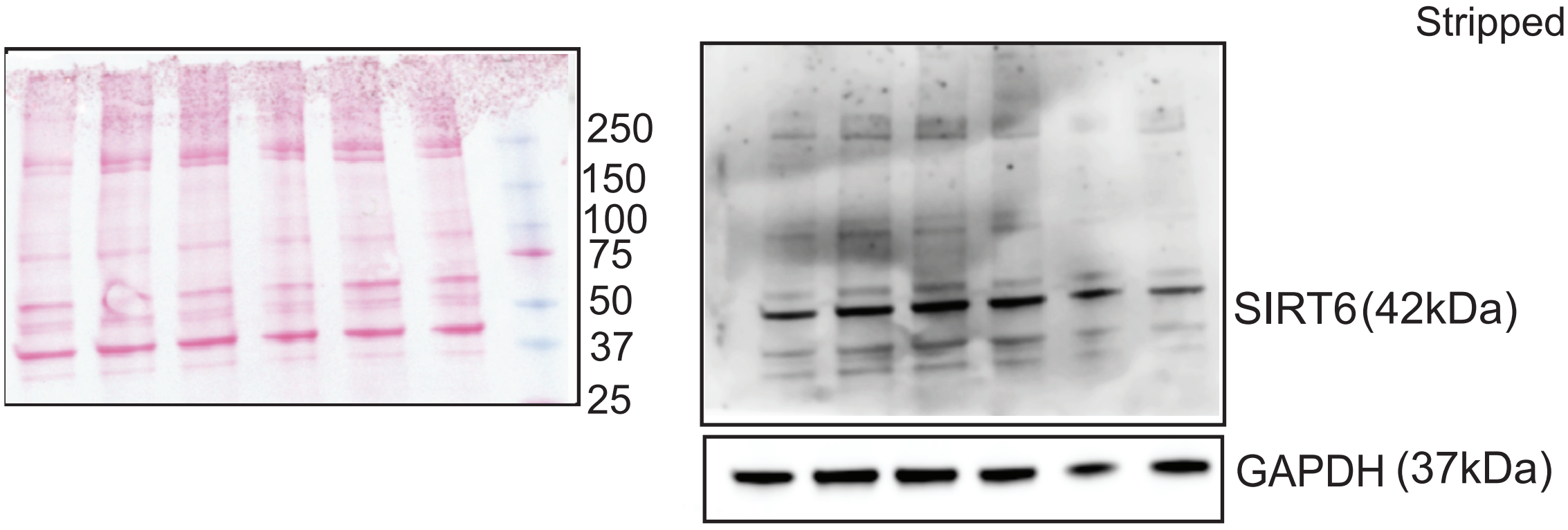

C Original image for 7A

First Probing - LC3
Stripped, second probing-GAPDH

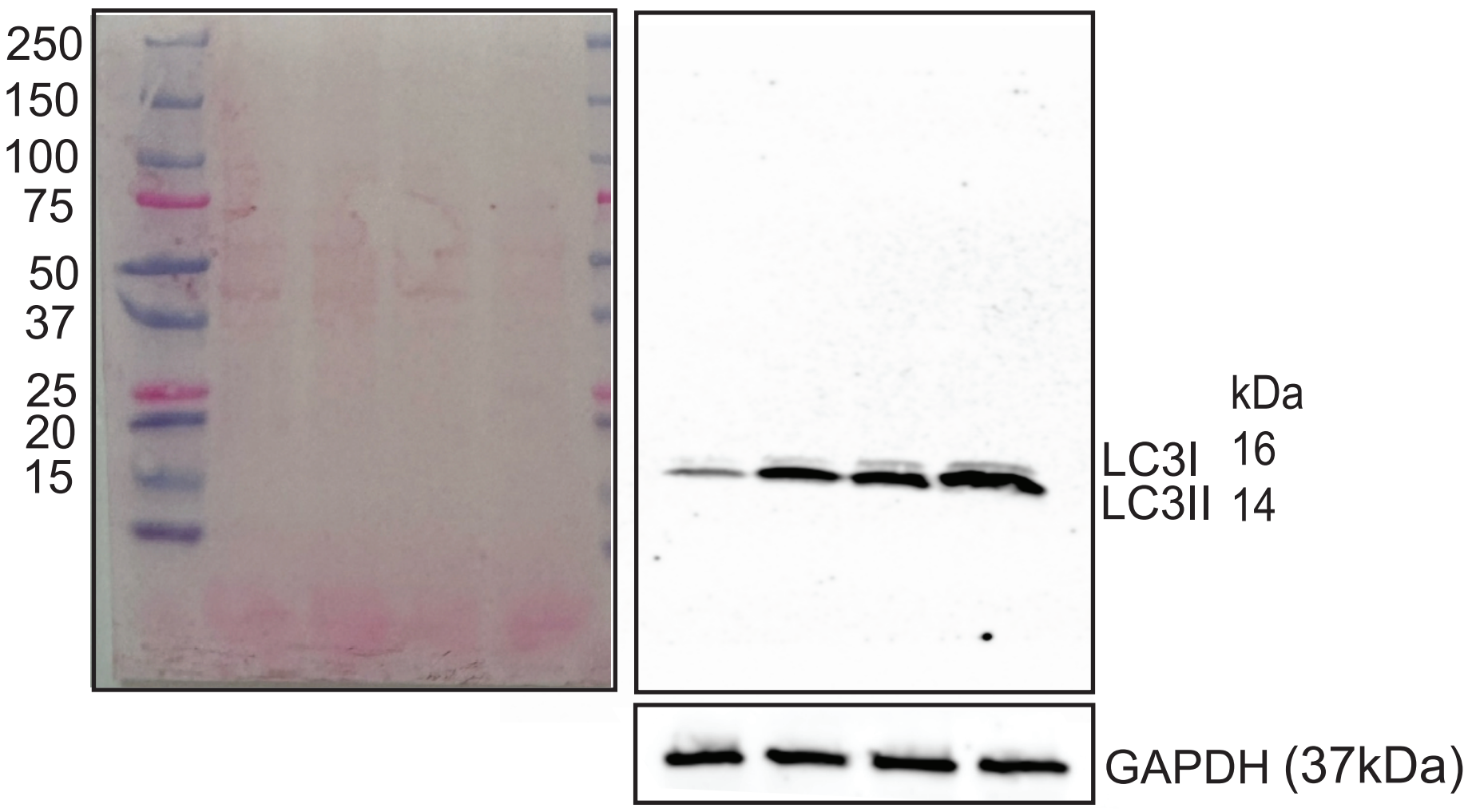

Supplement: Supplementary file 1 [file ACEL-25-e70709-s001.pdf]
