# Supplementary material for: SIRT6 Activation Improves Intervertebral Disc Health in the Aging Spine
Source: Aging Cell. 2026 Oct 19;25(10):e70709. doi: 10.1111/acel.70709 (PMC13679769; doi:10.1111/acel.70709)
Supplement: Supplementary file 2 [file ACEL-25-e70709-s002.pdf]

SIRT6 Activation Improves Intervertebral Disc Health in the Aging Spine

Supplemental Figure Legends

Fig. S1. Level-by-level Thompson grading scores of NP and AF compartments. Grading scores at spinal levels L3/4, L4/5, L5/6, and L6/S1 suggest that genetic, biological, and biomechanical factors at individual spinal levels result in distinct phenotypic outcomes (n=7 mice/group). Data are presented as median with interquartile range, and groups are compared using an unpaired t-test or Mann-Whitney test, as appropriate.

Fig. S2. LC/MS analysis shows minimal changes in plasma metabolome in MDL-treated mice. (A) Schematic of experimental design for plasma metabolomic analysis by LC/MS. (B) PCA plot shows no distinct clustering of samples based on the treatment. (C) Heatmaps of metabolite levels in individual mouse plasma samples and QC controls showing hierarchical clustering of samples. Only six metabolites, (D) palmitoyl sphingomyelin, (E) 5-aminolevulinic acid, (F) 2-oxindole, (G) jasmonic acid, (H) nicotinamide, and (I) hypotaurine, showed differential levels between the Vehicle and MDL-800 treatment groups. n = 8 Vehicle Control, and 7 MDL-800 mice. Significance was determined using an unpaired test or Mann-Whitney test, as appropriate.

Fig. S3. NMR analysis shows no alterations in plasma metabolic parameters following MDL-800 treatment. (A) Schematic of experimental design for plasma analysis using NMR. Levels of (B) GlycA, (C) Total Ketone Bodies, (D) Acetoacetate, (E) Acetone, (F) Beta Hydroxybutyrate (G) Total branched chained amino acids (BCAA) (H) Alanine, (I) Leucine, (J) Isoleucine, (K) Valine, (L) Citrate, (M) ApoA-1, showed no changes with MDL treatment, n = 8 Vehicle Control, and 7 MDL-800 mice. Significance was determined using an unpaired test or Mann-Whitney test, as appropriate.

Fig. S4. NMR analysis shows stable plasma lipid profile in MDL-800 treated mice Plasma NMR analysis showed no difference in levels of (A) ApoB, (B) Total Triglyceride, (C) Total Cholesterol, (D) Total TRLP (E) Small TRLP, (F) total calibrated low-density lipoprotein particle (cLDLP), (G) total calibrated high-density lipoprotein particle (cHDLP), (H) medium LDLP, (I) H4P, (J) TRL Size, (K) LDLC, (L) HDLC, (M) HDL Size, (N) TRL TG between Vehicle- and MDL-800 treated mice, n = 8 Vehicle Control, and 7 MDL-800 mice. Significance was determined using an unpaired test or Mann-Whitney test, as appropriate.

Fig. S5. SIRT6 activation promotes ECM homeostasis and decreases NP tissue fibrosis. (A) Representative bright field and (B) polarized images of Picrosirius Red-stained lumbar disc sections and (C) quantification of collagen fibers from VEH and MDL-800 treated mouse discs, n = 7 Vehicle Control, and 9 MDL-800 mice; 2-4 discs/mouse. Scale bar= 200 μm. (D) Quantification of NP compartment fibrosis using polarized images. (E-F) Representative immunofluorescence images of lumbar disc sections and (G, H) corresponding quantification for Aggrecan (ACAN) and GLUT1. Scale bar = 50 μm, n = 6 mice/group and 2-4 discs/animal. White dotted lines demarcate disc compartments. Quantitative measurements are reported as the median with the interquartile range, and significance was determined by using an unpaired *t*-test or Mann-Whitney test, as appropriate.

Fig. S6. SIRT6 activation does not alter the overall abundance of Collagen I and X in the disc. (A-F) Representative immunofluorescence images of lumbar disc sections and respective quantitation for (A, B) FCHP, (C, D) COL1, and (E, F) COLX in 24-month-old vehicle- and MDL-800 treated mice, scale bar = 50 μm. n = 6 mice/group, 2-4 discs/animal. White dotted lines demarcate disc compartments. Significance was determined using an unpaired t-test

Fig. S7. SIRT6 activation suppresses senescence of intervertebral disc cells. (A-D) Representative immunofluorescence images and corresponding quantitative analysis of (A, B) MMP13 and (C, D) CCL2. n = 4 animals/group, 3 discs/mouse. Scale bar = 200 μm. (E-G) Gene expression of senescence marker (E) *MMP-13*, (F) *CCL-2* and (G) *p16* from Grade IV and V human NP cells following stimulation (Stim) with

proinflammatory cytokines IL-1β and IL-6 and MDL-800 co-treatment with stimulation (MDL+Stim). Values are represented as mean ± SD. Significance was determined using One-way ANOVA followed by Tukey's multiple comparison test (n= 4 biological replicates).

Fig. S8. SIRT6 activation results in changes in the transcriptomic landscape of disc tissue. RNA-sequencing of NP and AF tissue transcripts from Vehicle and MDL-800 treated 24-month-old mice represented as (A, B) Heat map and hierarchical clustering of Z-score of the top differentially expressed genes (DEGs) from (A) NP and (B) AF tissues from MDL-800 and Vehicle-treated mice (FC≥1.75, $p \leq 0.05$). (C, D) Volcano plot of DEGs in the (C) NP and (D) AF showing *p*-value versus magnitude of change (fold change), n = 5 mice/group.

Fig. S9. SIRT6 activation results in changes in NP tissue gene expression. (A) CompBio analysis of Downregulated DEGs (FC≥1.75, p<0.05) in NP tissue of 24-month-old MDL-800 treated B6 mice represented in a ball and stick model, n=5 mice/group, 5-6 pooled discs/animal. The enrichment of themes is shown by the size of the ball, and connectedness is shown based on the thickness of the lines between them. Themes of interest are colored, and superclusters comprised of related themes are highlighted. (B) Top thematic DEGs plotted based on CompBio entity enrichment score.

Fig. S10. SIRT6 activation results in transcriptomic changes in AF tissue. (A) CompBio analysis of Upregulated DEGs (FC≥1.75, p<0.05) in AF tissue of 24-month-old MDL-800 treated B6 mice represented in a ball and stick model, n=5 mice/group, 5-6 pooled discs/animal. The enrichment of themes is shown by the size of the ball, and connectedness is shown based on the thickness of the lines between them. Themes of interest are colored, and superclusters comprised of related themes are highlighted. (B) Top thematic DEGs plotted based on CompBio entity enrichment score.

Fig. S11. SIRT6 activation results in gene expression changes in AF tissue. (A) CompBio analysis of Downregulated DEGs (FC≥1.75, p<0.05) in AF tissue of 24-

month-old MDL-800 treated B6 mice represented in a ball and stick model, n=5 mice/group, 5-6 pooled discs/animal. The enrichment of themes is shown by the size of the ball, and connectedness is shown based on thickness of the lines between them. Themes of interest are colored, and superclusters comprised of related themes are highlighted. (B) Top thematic DEGs plotted based on CompBio entity enrichment score.

Fig. S12. MDL-800 treated mice exhibit changes in bone marrow immune cell profiles. (A) Schematic showing the isolation and scRNA-sequencing of bone marrow cells from vehicle- and MDL-800 treated mice. (B) UMAP showing unbiased clustering of bone marrow cells using Seurat. (C) UMAP with the annotation of Seurat clusters according to treatment. (D) UMAP with the annotation of cells using SingleR. (E) Number and proportion of cells identified in each treatment cohort, with odds ratios and p-values. Volcano plots showing differential gene expression in (F) Monocytes and (G) Granulocytes.

Fig. S13. MDL-800 treated mice exhibit changes in T cell profiles. (A) UMAP showing splenic T cells, labeled according to treatment cohort. (B) Cd4+ T cells and (C) Cd8a+ T cells within the splenic T cell clusters. (D, D’) The number and proportion of Cd4+ and Cd8a+ T cells, with odds ratio and p-value. Volcano plots showing differential gene expression in (E) Cd4+ T Cells and (F) Cd8a+ T Cells. (G) UMAP showing bone marrow T cells, labeled according to treatment cohort. (H) Cd4+ T cells and (I) Cd8a+ T cells within the bone marrow-derived T cell clusters. (J, J’) The number and proportion of Cd4+ and Cd8a+ T cells, with odds ratio and p-value.

Fig. S14. Plasma analysis of MDL-800 treated mice does not show increased systemic inflammation. Multiplex analysis showed no significant changes in the plasma concentrations of (A) IFN-γ, (B) IL-1β, (C) IL-2, (D) IL-4, (E) IL-5, (F) IL-6, (G) IL-10, (H) IL-12/p70, (I) IL-15, (J) IL-17A/F, (K) IL-27/p28/IL-30, (L) IL-33, (M) IP-10, (N) KC/GRO, (O) MCP-1, (P) MIP-1α, (Q) MIP-2, (R) TNF-α in MDL-800 treated mice compared to Vehicle-treated mice (n =10 mice/group). Significance was determined using an unpaired t-test or Mann-Whitney test, as appropriate.

Fig. S15. Effect of MDL-800 treatment on H3 acetylation and methylation marks (A-M) Quantitative ELISA showing Histone 3 modifications in VEH and MDL-800 treated NP cells, n=3 independent experiments. Significance for quantitative measures was determined by using an unpaired *t*-test.

Fig. S16. SIRT6 activation in NP cells results in clustering of upregulated DEGs in themes related to the cytoskeleton, cell junctions, skeletal tissues, extracellular matrix, and purinergic signaling. (A) CompBio analysis generated a thematic map of upregulated DEGs from an RNA sequencing experiment ($Log_2FC \geq 1.5$, FDR < 0.05) comparing MDL-800-treated vs VEH-treated NP cells, represented as a ball-and-stick model. Themes of interest are colored, and superclusters comprised of related themes are highlighted. (B) Top thematic DEGs plotted based on CompBio entity enrichment score. n = 4 independent experiments.

Fig. S17. SIRT6 activation in NP cells results in clustering of downregulated DEGs in themes related to fatty acid β-oxidation, PPAR signaling, and Hsp70/Hsc70 chaperons and Jagged1-Notch1 signaling. (A) CompBio analysis generated a thematic map of downregulated DEGs ($Log_2FC \geq 1.5$, FDR < 0.05) from the RNA sequencing experiment of MDL-800-treated vs VEH-treated NP cells, represented as a ball-and-stick model. Themes of interest are colored, and superclusters comprised of related themes are highlighted. (B) Top thematic DEGs plotted based on CompBio entity enrichment score. n = 4 independent experiments.

Fig. S18. **DEGs in NP tissue from MDL-800-treated mice share the thematic commonality with NP cells treated with MDL-800 in vitro.** Territorial maps of CompBio Assertion engine analysis showing common themes derived from (A) upregulated and (B) downregulated DEGs from NP tissues of MDL-800 treated mice and MDL-800 treated cultured NP cells.

Fig. S19. Multiomics analysis of ATACseq and RNAseq of MDL-800-treated NP cells shows a good overlap in DEGs. (A) Heatmaps showing chromatin accessibility signal profiles in Vehicle- and MDL-800-treated samples, average signal profiles are shown above the heatmaps. n = 3 independent experiments. (B) Stacked bars showing the average relative distribution of ATAC-seq peaks in Vehicle- and MDL-800

treated NP cells, annotated to genomic features including promoters, introns, exons, and distal regions. n = 3 independent experiments. (C) Heatmap of top 50 up and 21 down DEGs shared between RNA-seq and ATAC-seq (FC ≥ 2, FDR $\leq$ 0.05), sorted based on ATAC-seq fold-change intensity. (D) Quadrant map of commonly upregulated and downregulated DEGs between ATAC-seq and RNA-seq of MDL-800 treated NP cells (FC ≥ 2, FDR $\leq$ 0.05), mapped to one of the thematic superclusters in RNA-Seq data shown in Figure 8 and Figure S13. (E) Gene tracks showing enriched peaks for *Serpine1* and *Evp1* shared across ATAC-seq and RNA-seq (*in vitro* and *in vivo*) analyses. ATACseq n = 3 experiments, in vitro RNAseq n= 4 independent experiments, in vivo RNAseq n = 5 mice/group.

Fig. S20. CompBio analysis of unique DEGs shared between RNAseq and ATACseq datasets of NP cells treated with MDL-800. Thematic map of unique DEGs shared between RNAseq and ATACseq ($Log_2FC \geq 1.5$, FDR < 0.05) from MDL-800-treated vs VEH-treated NP cells, represented as a ball-and-stick model. (A) Map of upregulated DEGs and (B) downregulated DEGs.

Fig. S21. MDL-800 treatment in B6 mice affects vertebral bone structural parameters. Representative µCT reconstructions showing (A) transverse section through the lumbar vertebra of 24-month-old VEH and MDL-treated mice. (B-E) Trabecular bone properties, (B) BV/TV, (C) Tb. N. (trabecular number), (D) Tb. Th. (trabecular thickness), (E) Trab. Sp. (trabecular separation) (F-I) Cortical bone properties showing (F) closed porosity (%), (G) Cs. Th. (cross-sectional thickness), (H) Cs. Ar. (cross-sectional bone area) and (I) Cs. Pm. (cross-sectional perimeter) are shown, n =8 Vehicle Control, and 12 MDL-800 mice, 4 vertebrae/mouse analyzed. Statistical differences between groups were tested using an unpaired t-test; $p < 0.05$.

Fig. S22. Long-term MDL treatment has minimal effects on behaviors associated with pain and ambulation in old B6 mice. (A) Schematic of the experimental setup of the behavioral assays. Sensitization analysis: (B, C) mechanical (Von Frey), left and right paw, (D) hot (Hargreaves), and (E) cold stimulus. Ambulation, frailty, and axial pain measurements: (F) Open field (G) Grip strength and Gait analysis parameters. (H) Swing Duration (I) Stance duration, (J) Stride duration (K) stride length, (L) swing time, (M) Stride frequency, n = 7 Vehicle

Control, n = 9 MDL-800 mice. Statistical difference between groups was tested using an unpaired t-test or a Mann-Whitney test, as appropriate.

Figure S. 1

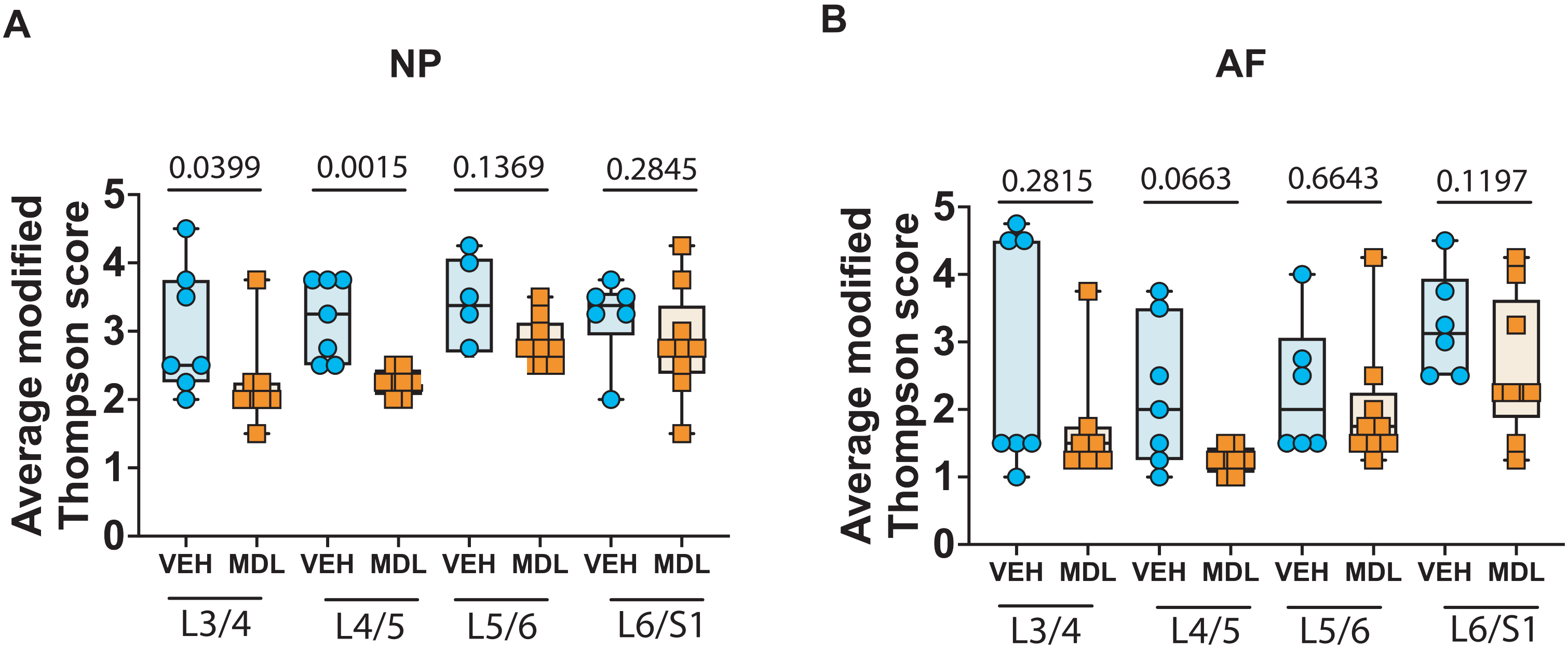
A
NP
0.0399
0.0015
0.1369
0.2845
Average modified Thompson score
5
4
3
2
1
0
VEH
MDL
VEH
MDL
VEH
MDL
VEH
MDL
L3/4
L4/5
L5/6
L6/S1
B
AF
0.2815
0.0663
0.6643
0.1197
Average modified Thompson score
5
4
3
2
1
0
VEH
MDL
VEH
MDL
VEH
MDL
VEH
MDL
L3/4
L4/5
L5/6
L6/S1

Figure S.2

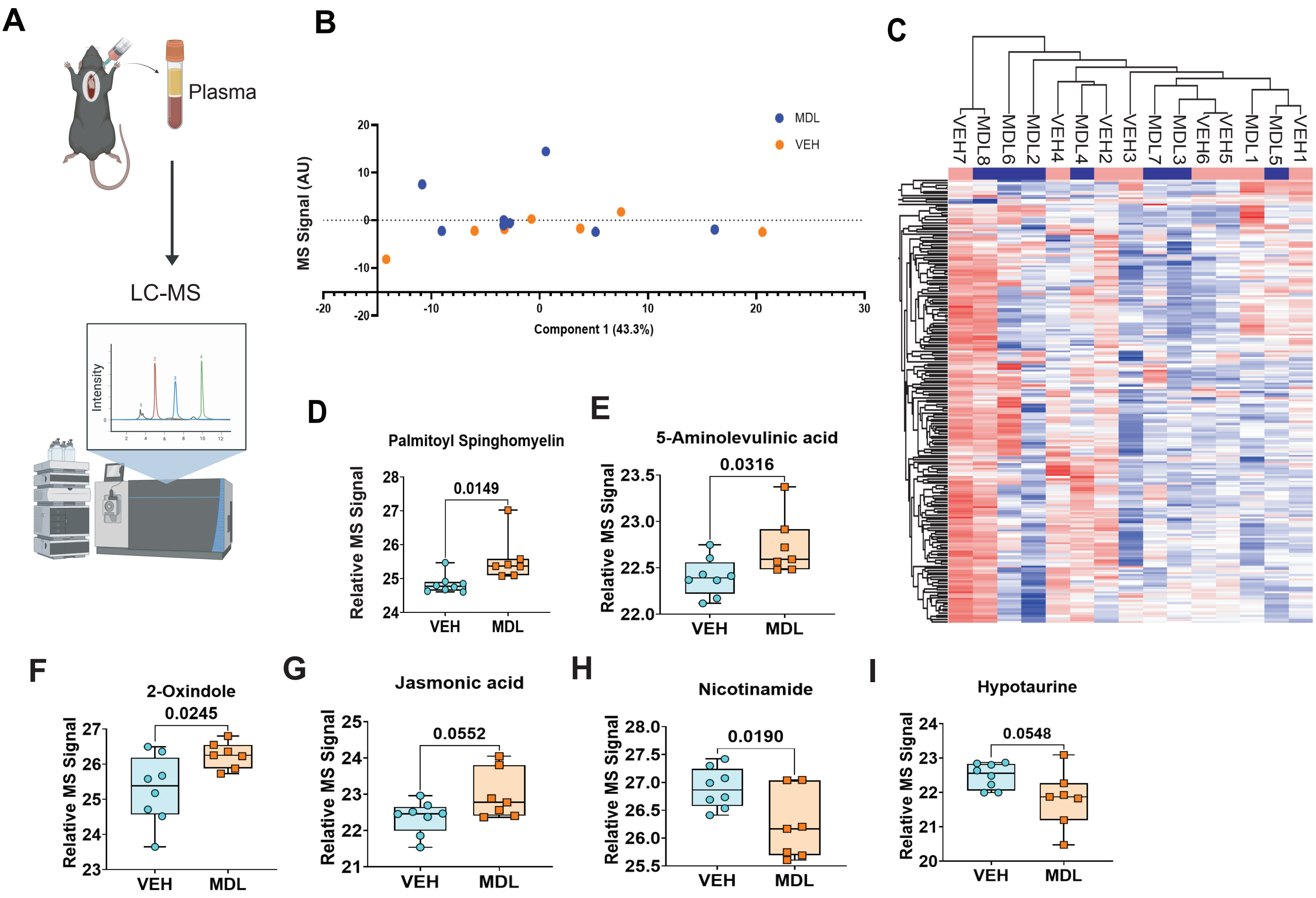
A
Plasma
LC-MS
Intensity
B
MS Signal (AU)
Component 1 (43.3%)
MDL
VEH
C
VEH7
MDL8
MDL6
MDL2
VEH4
MDL4
VEH2
VEH3
MDL7
MDL3
VEH6
VEH5
MDL1
MDL5
VEH1
D
Palmitoyl Spinghomyelin
0.0149
Relative MS Signal
VEH
MDL
E
5-Aminolevulinic acid
0.0316
Relative MS Signal
VEH
MDL
F
2-Oxindole
0.0245
Relative MS Signal
VEH
MDL
G
Jasmonic acid
0.0552
Relative MS Signal
VEH
MDL
H
Nicotinamide
0.0190
Relative MS Signal
VEH
MDL
I
Hypotaurine
0.0548
Relative MS Signal
VEH
MDL

Figure S.3

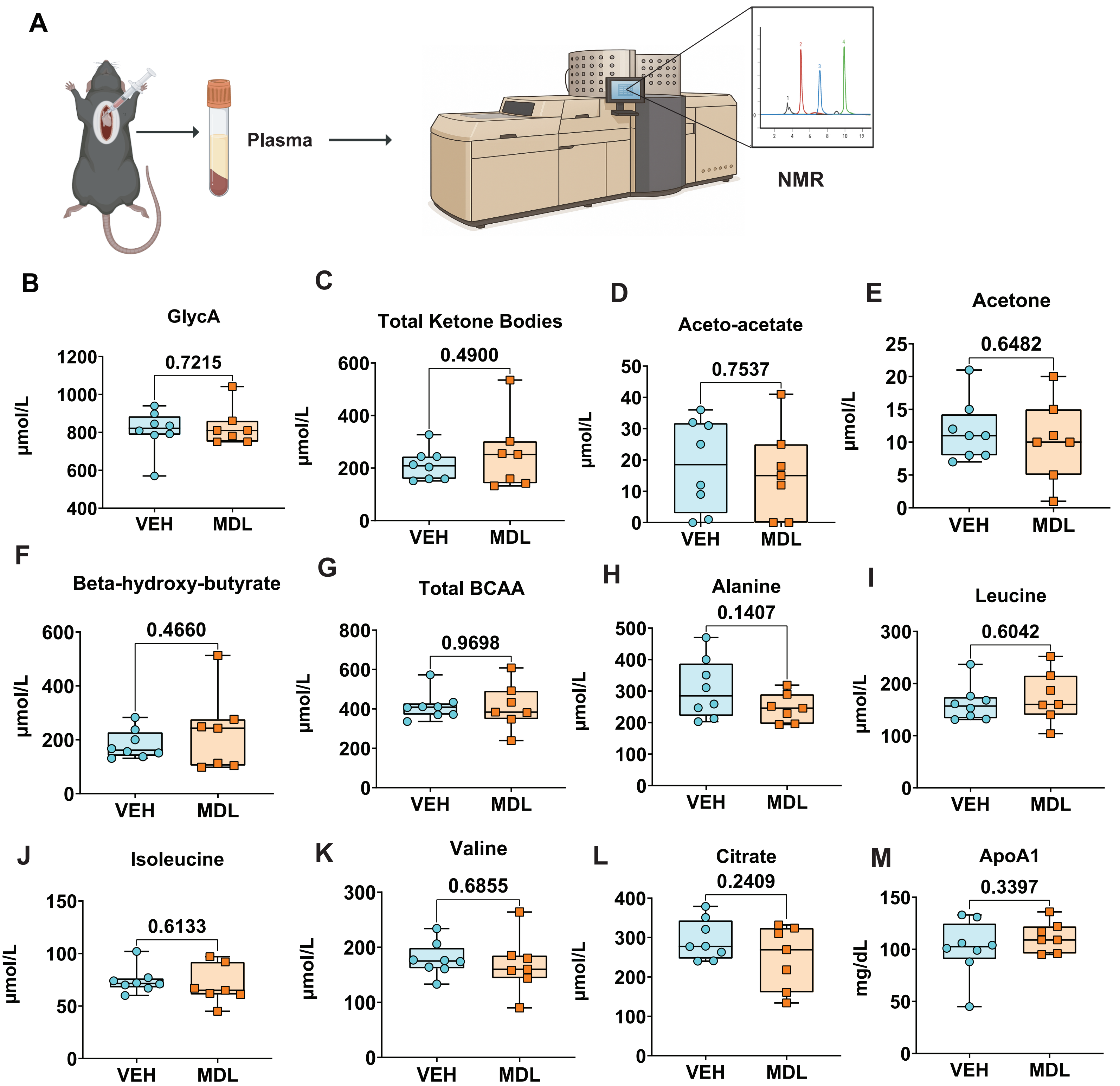

A
Plasma
NMR
B
GlycA
0.7215
µmol/L
VEH
MDL
C
Total Ketone Bodies
0.4900
µmol/L
VEH
MDL
D
Aceto-acetate
0.7537
µmol/L
VEH
MDL
E
Acetone
0.6482
µmol/L
VEH
MDL
F
Beta-hydroxy-butyrate
0.4660
µmol/L
VEH
MDL
G
Total BCAA
0.9698
µmol/L
VEH
MDL
H
Alanine
0.1407
µmol/L
VEH
MDL
I
Leucine
0.6042
µmol/L
VEH
MDL
J
Isoleucine
0.6133
µmol/L
VEH
MDL
K
Valine
0.6855
µmol/L
VEH
MDL
L
Citrate
0.2409
µmol/L
VEH
MDL
M
ApoA1
0.3397
mg/dL
VEH
MDL

Figure S.4

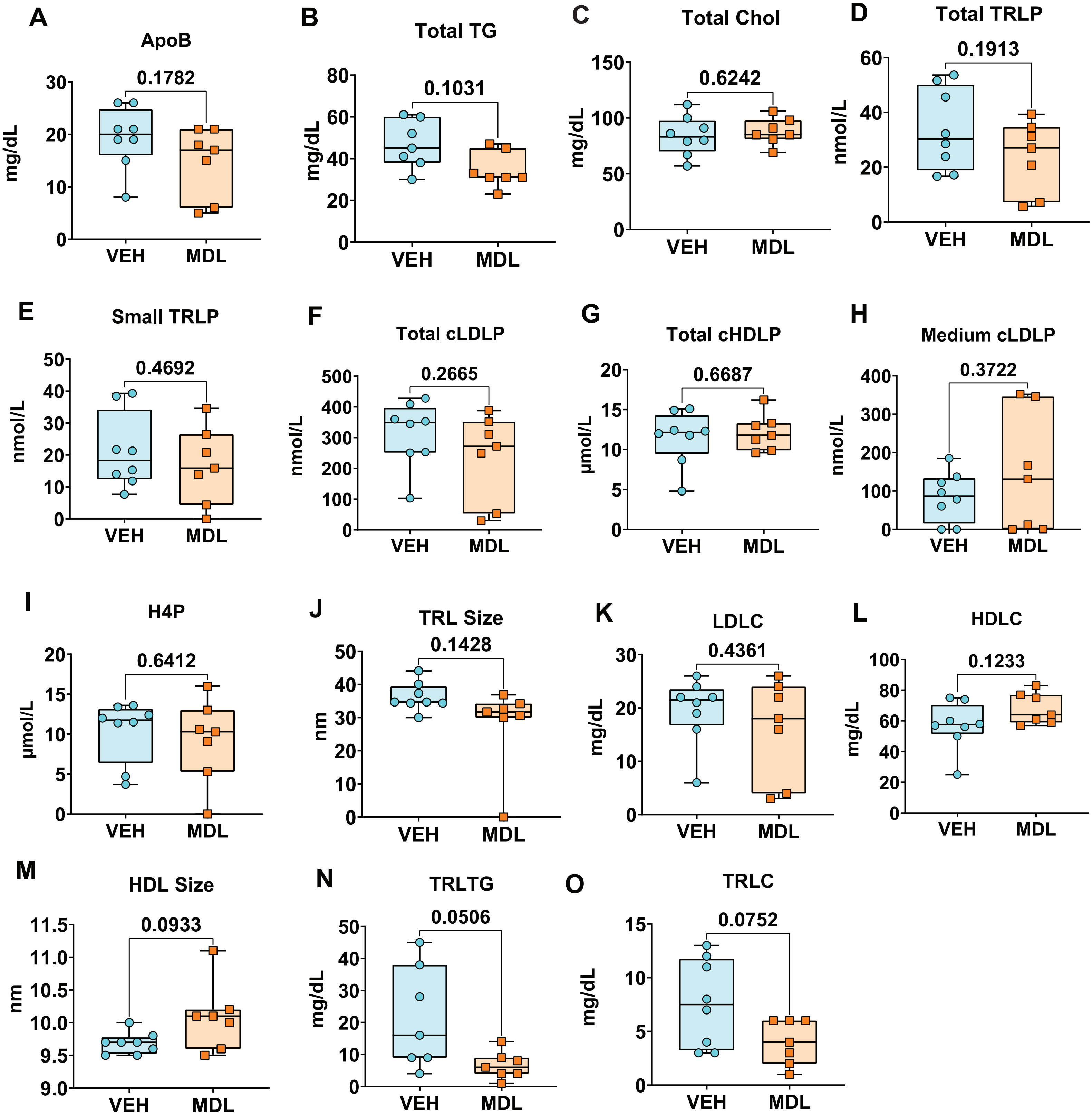

A
ApoB
0.1782
mg/dL
VEH
MDL
B
Total TG
0.1031
mg/dL
C
Total Chol
0.6242
mg/dL
D
Total TRLP
0.1913
nmol/L
E
Small TRLP
0.4692
nmol/L
F
Total cLDLP
0.2665
nmol/L
G
Total cHDLP
0.6687
μmol/L
H
Medium cLDLP
0.3722
nmol/L
I
H4P
0.6412
μmol/L
J
TRL Size
0.1428
nm
K
LDLC
0.4361
mg/dL
L
HDLC
0.1233
mg/dL
M
HDL Size
0.0933
nm
N
TRLTG
0.0506
mg/dL
O
TRLC
0.0752
mg/dL

Figure S.5

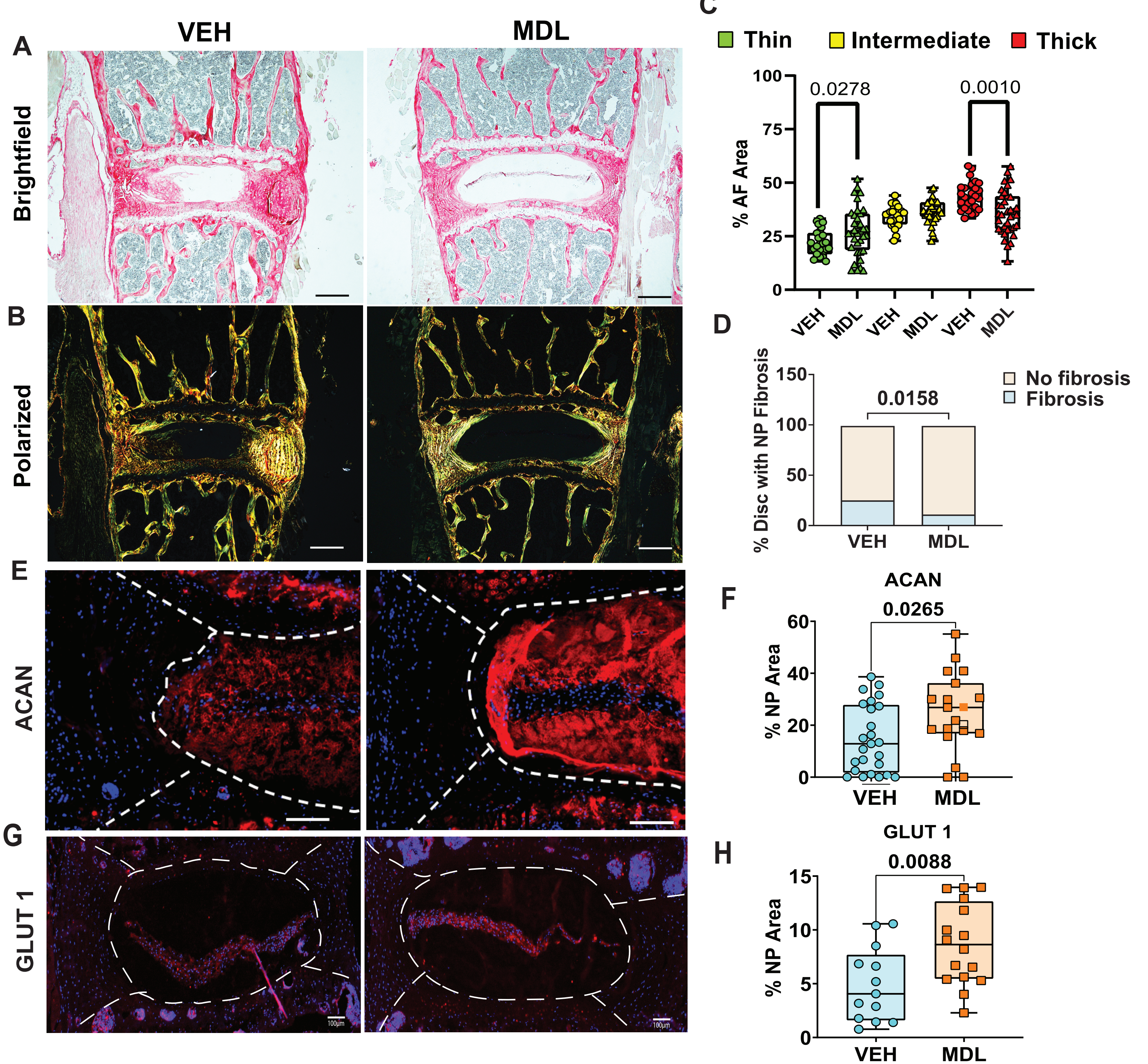

A
VEH
MDL
Brightfield
B
Polarized
C
Thin
Intermediate
Thick
% AF Area
100
75
50
25
0
0.0278
0.0010
VEH
MDL
VEH
MDL
VEH
MDL
D
% Disc with NP Fibrosis
150
100
50
0
0.0158
No fibrosis
Fibrosis
VEH
MDL
E
ACAN
F
ACAN
0.0265
% NP Area
60
40
20
0
VEH
MDL
G
GLUT 1
100µm
100µm
H
GLUT 1
0.0088
% NP Area
15
10
5
0
VEH
MDL

Figure S.6

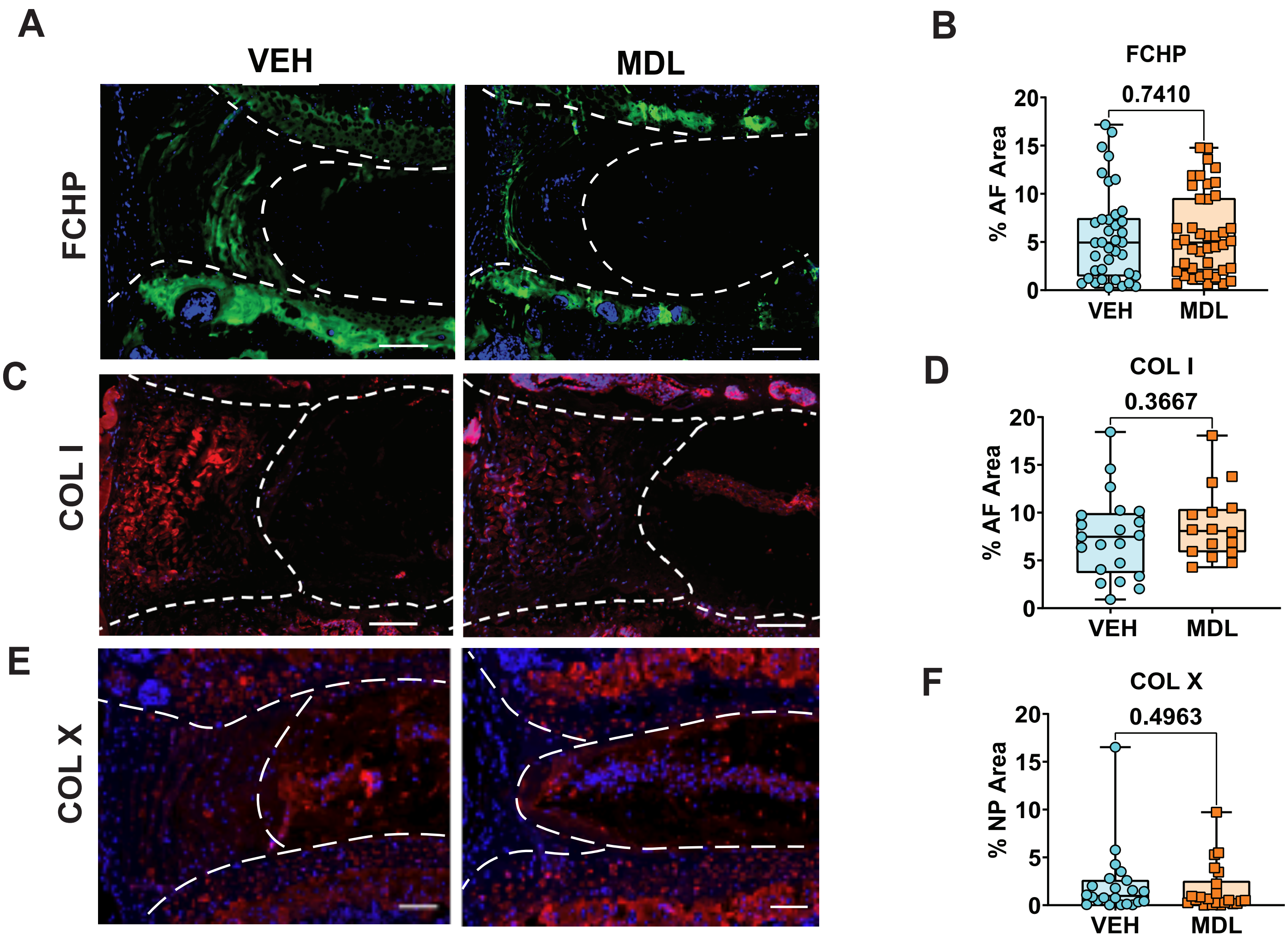

A
VEH
MDL
FCHP
B
FCHP
0.7410
% AF Area
20
15
10
5
0
VEH
MDL
C
COL I
D
COL I
0.3667
% AF Area
20
15
10
5
0
VEH
MDL
E
COL X
F
COL X
0.4963
% NP Area
20
15
10
5
0
VEH
MDL

Figure S.7

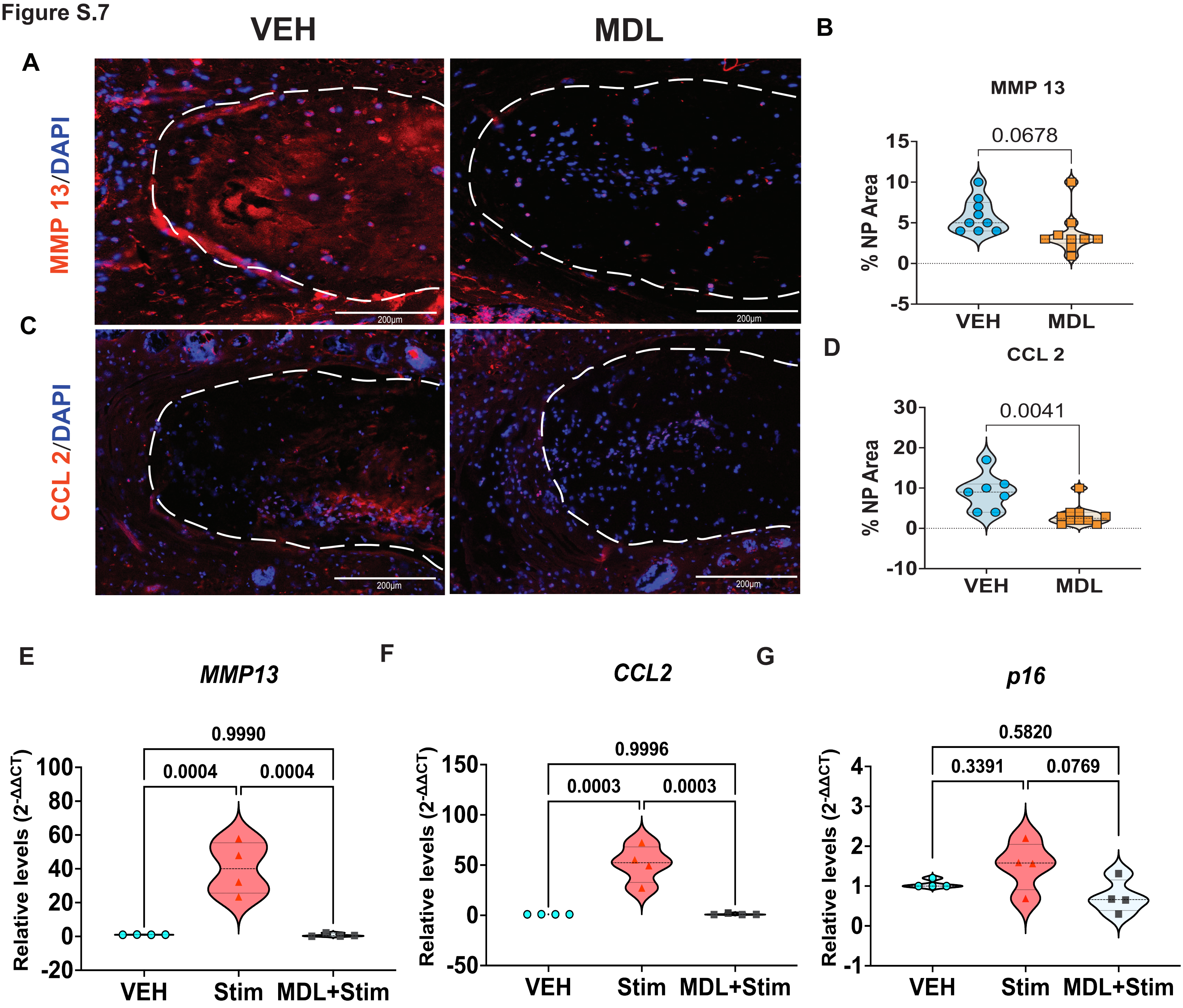

VEH
MDL
A
MMP 13/DAPI
200μm
200μm
B
MMP 13
0.0678
% NP Area
15
10
5
0
-5
VEH
MDL
C
CCL 2/DAPI
200μm
200μm
D
CCL 2
0.0041
% NP Area
30
20
10
0
-10
VEH
MDL
E
MMP13
0.9990
0.0004
0.0004
Relative levels (2^-ΔΔCT)
100
80
60
40
20
0
-20
VEH
Stim
MDL+Stim
F
CCL2
0.9996
0.0003
0.0003
Relative levels (2^-ΔΔCT)
150
100
50
0
-50
VEH
Stim
MDL+Stim
G
p16
0.5820
0.3391
0.0769
Relative levels (2^-ΔΔCT)
4
3
2
1
0
-1
VEH
Stim
MDL+Stim

Figure S.8

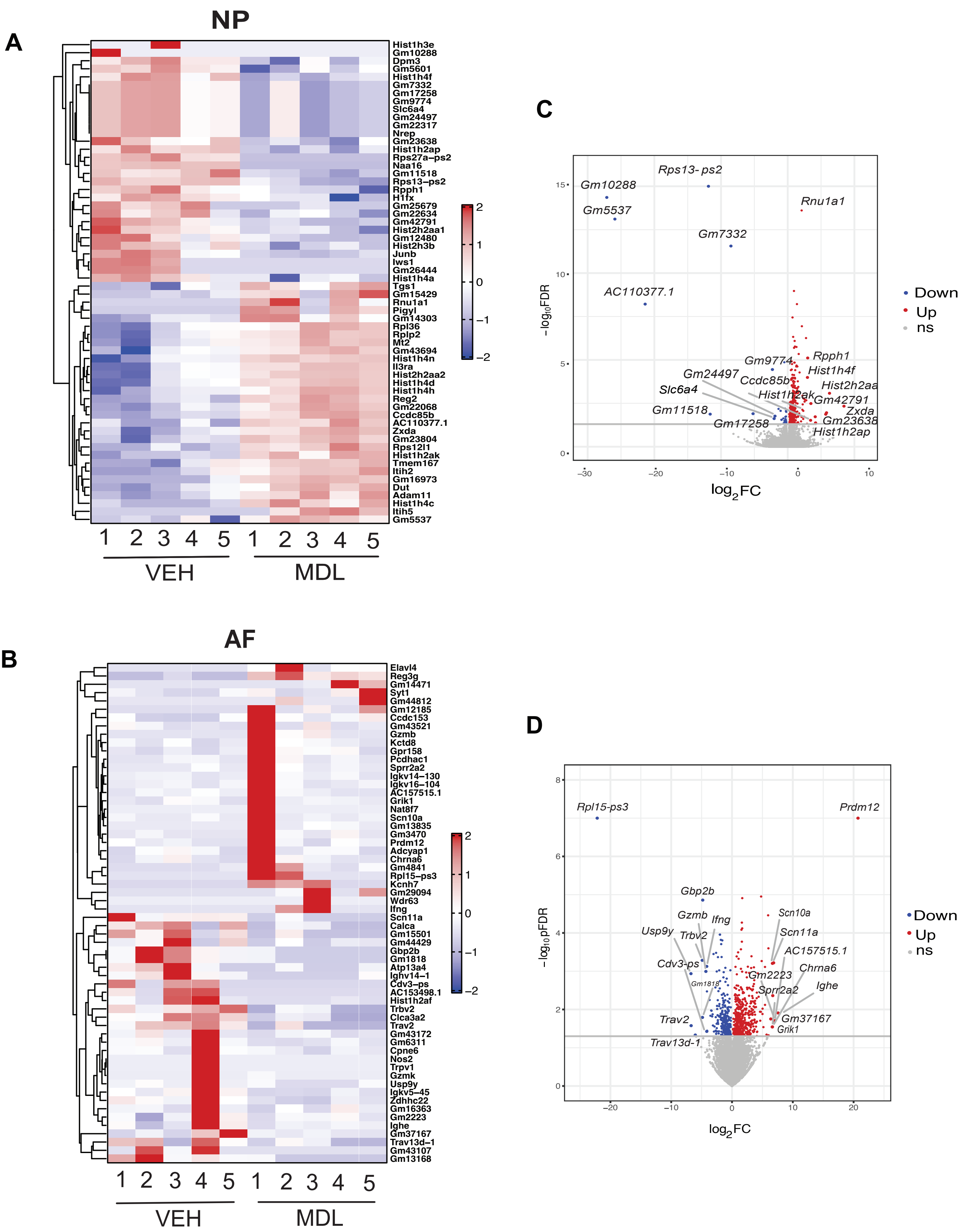
A
NP
Hist1h3e
Gm10288
Dpm3
Gm5601
Hist1h4f
Gm7332
Gm17258
Gm9774
Slc6a4
Gm24497
Gm22317
Nrep
Gm23638
Hist1h2ap
Rps27a−ps2
Naa16
Gm11518
Rps13−ps2
Rpph1
H1fx
Gm25679
Gm22634
Gm42791
Hist2h2aa1
Gm12480
Hist2h3b
Junb
Iws1
Gm26444
Hist1h4a
Tgs1
Gm15429
Rnu1a1
Pigyl
Gm14303
Rpl36
Rplp2
Mt2
Gm43694
Hist1h4n
Il3ra
Hist2h2aa2
Hist1h4d
Hist1h4h
Reg2
Gm22068
Ccdc85b
AC110377.1
Zxda
Gm23804
Rps12l1
Hist1h2ak
Tmem167
Itih2
Gm16973
Dut
Adam11
Hist1h4c
Itih5
Gm5537
2
1
0
−1
−2
1 2 3 4 5 1 2 3 4 5
VEH
MDL
B
AF
Elavl4
Reg3g
Gm14471
Syt1
Gm44812
Gm12185
Ccdc153
Gm43521
Gzmb
Kctd8
Gpr158
Pcdhac1
Sprr2a2
Igkv14−130
Igkv16−104
AC157515.1
Grik1
Nat8f7
Scn10a
Gm13835
Gm3470
Prdm12
Adcyap1
Chrna6
Gm4841
Rpl15−ps3
Kcnh7
Gm29094
Wdr63
Ifng
Scn11a
Calca
Gm15501
Gm44429
Gbp2b
Gm1818
Atp13a4
Ighv14−1
Cdv3−ps
AC153498.1
Hist1h2af
Trbv2
Clca3a2
Trav2
Gm43172
Gm6311
Cpne6
Nos2
Trpv1
Gzmk
Usp9y
Igkv5−45
Zdhhc22
Gm16363
Gm2223
Ighe
Gm37167
Trav13d−1
Gm43107
Gm13168
2
1
0
−1
−2
1 2 3 4 5 1 2 3 4 5
VEH
MDL
C
−log10FDR
log2FC
Rps13- ps2
Gm10288
Gm5537
Rnu1a1
Gm7332
AC110377.1
Gm9774
Rpph1
Hist1h4f
Gm24497
Ccdc85b
Hist2h2aa
Slc6a4
Hist1h2ak
Gm42791
Gm11518
Zxda
Gm17258
Gm23638
Hist1h2ap
15
10
5
0
−30
−20
−10
0
10
Down
Up
ns
D
−log10pFDR
log2FC
Rpl15-ps3
Prdm12
Gbp2b
Gzmb
Ifng
Scn10a
Usp9y
Trbv2
Scn11a
AC157515.1
Cdv3-ps
Chrna6
Gm2223
Gm1818
Sprr2a2
Ighe
Trav2
Gm37167
Grik1
Trav13d-1
8
6
4
2
0
−20
−10
0
10
20
Down
Up
ns

Figure S.9

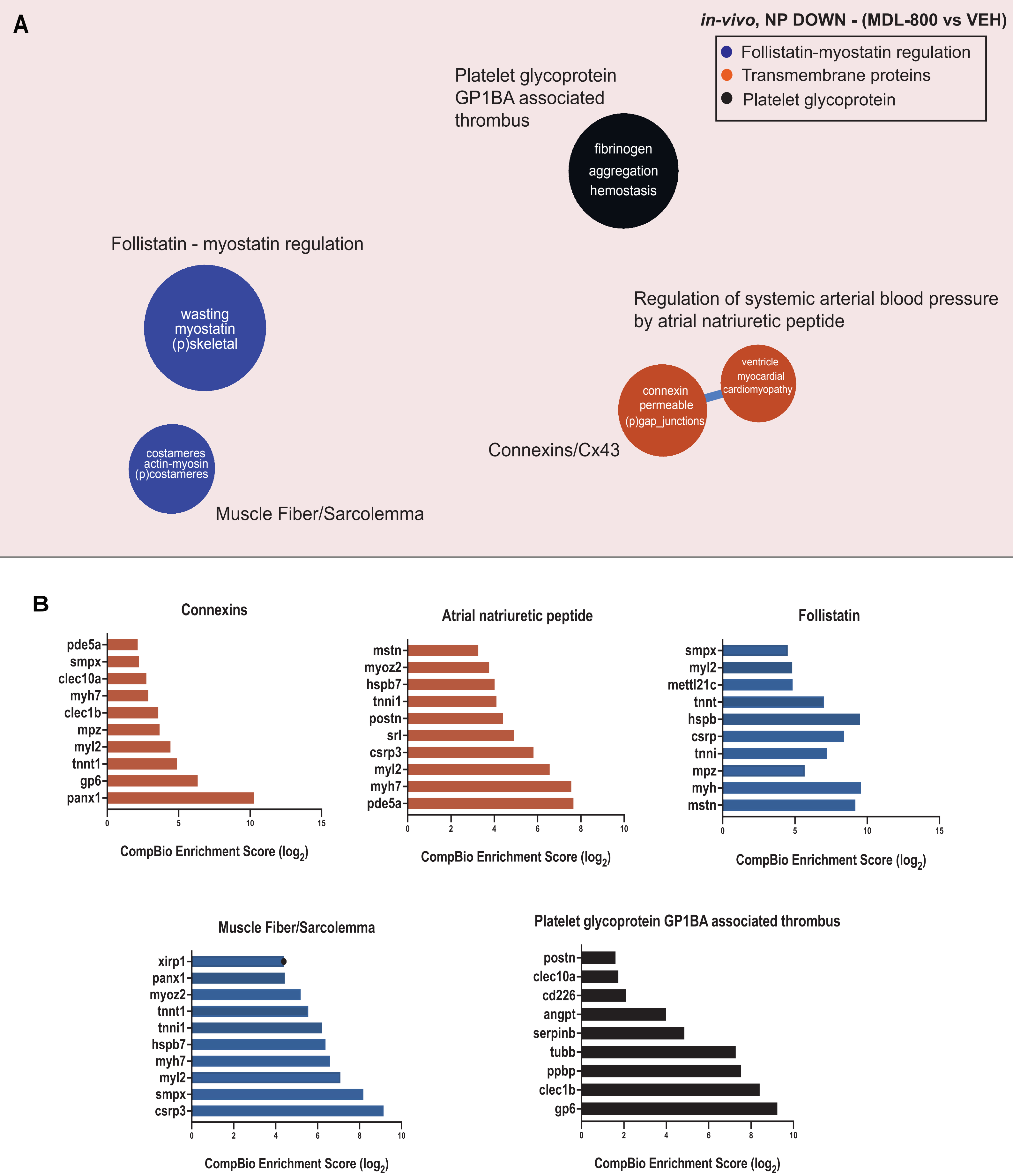

A
in-vivo, NP DOWN - (MDL-800 vs VEH)
Follistatin-myostatin regulation
Transmembrane proteins
Platelet glycoprotein
Platelet glycoprotein GP1BA associated thrombus
fibrinogen
aggregation
hemostasis
Follistatin - myostatin regulation
wasting
myostatin
(p)skeletal
Regulation of systemic arterial blood pressure by atrial natriuretic peptide
ventricle
myocardial
cardiomyopathy
connexin
permeable
(p)gap_junctions
Connexins/Cx43
costameres
actin-myosin
(p)costameres
Muscle Fiber/Sarcolemma
B
Connexins
pde5a
smpx
clec10a
myh7
clec1b
mpz
myl2
tnnt1
gp6
panx1
CompBio Enrichment Score (log2)
Atrial natriuretic peptide
mstn
myoz2
hspb7
tnni1
postn
srl
csrp3
myl2
myh7
pde5a
CompBio Enrichment Score (log2)
Follistatin
smpx
myl2
mettl21c
tnnt
hspb
csrp
tnni
mpz
myh
mstn
CompBio Enrichment Score (log2)
Muscle Fiber/Sarcolemma
xirp1
panx1
myoz2
tnnt1
tnni1
hspb7
myh7
myl2
smpx
csrp3
CompBio Enrichment Score (log2)
Platelet glycoprotein GP1BA associated thrombus
postn
clec10a
cd226
angpt
serpinb
tubb
ppbp
clec1b
gp6
CompBio Enrichment Score (log2)

Figure S.10

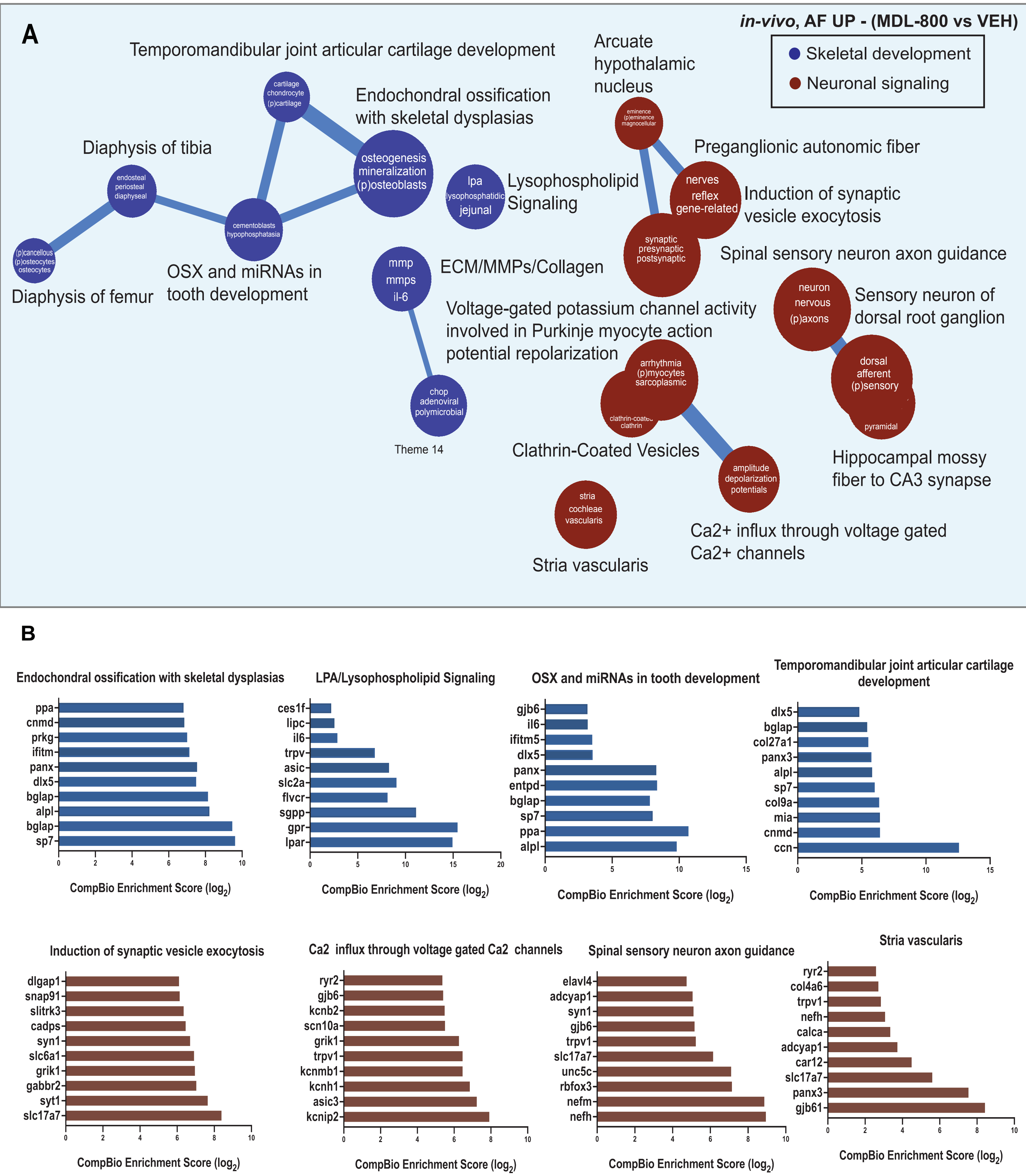
A
in-vivo, AF UP - (MDL-800 vs VEH)
Skeletal development
Neuronal signaling
Temporomandibular joint articular cartilage development
cartilage chondrocyte (p)cartilage
Endochondral ossification with skeletal dysplasias
osteogenesis mineralization (p)osteoblasts
Diaphysis of tibia
endosteal periosteal diaphyseal
cementoblasts hypophosphatasia
(p)cancellous (p)osteocytes osteocytes
Diaphysis of femur
OSX and miRNAs in tooth development
lpa lysophosphatidic jejunal
Lysophospholipid Signaling
mmp mmps il-6
ECM/MMPs/Collagen
chop adenoviral polymicrobial
Theme 14
Arcuate hypothalamic nucleus
eminence (p)eminence magnocellular
Preganglionic autonomic fiber
nerves reflex gene-related
Induction of synaptic vesicle exocytosis
synaptic presynaptic postsynaptic
Spinal sensory neuron axon guidance
neuron nervous (p)axons
Sensory neuron of dorsal root ganglion
dorsal afferent (p)sensory
pyramidal
Hippocampal mossy fiber to CA3 synapse
Voltage-gated potassium channel activity involved in Purkinje myocyte action potential repolarization
arrhythmia (p)myocytes sarcoplasmic
clathrin-coated clathrin
Clathrin-Coated Vesicles
amplitude depolarization potentials
Ca2+ influx through voltage gated Ca2+ channels
stria cochleae vascularis
Stria vascularis
B
Endochondral ossification with skeletal dysplasias
ppa, cnmd, prkg, ifitm, panx, dlx5, bglap, alpl, bglap, sp7
LPA/Lysophospholipid Signaling
ces1f, lipc, il6, trpv, asic, slc2a, flvcr, sgpp, gpr, lpar
OSX and miRNAs in tooth development
gjb6, il6, ifitm5, dlx5, panx, entpd, bglap, sp7, ppa, alpl
Temporomandibular joint articular cartilage development
dlx5, bglap, col27a1, panx3, alpl, sp7, col9a, mia, cnmd, ccn
Induction of synaptic vesicle exocytosis
dlgap1, snap91, slitrk3, cadps, syn1, slc6a1, grik1, gabbr2, syt1, slc17a7
Ca2 influx through voltage gated Ca2 channels
ryr2, gjb6, kcnb2, scn10a, grik1, trpv1, kcnmb1, kcnh1, asic3, kcnip2
Spinal sensory neuron axon guidance
elavl4, adcyap1, syn1, gjb6, trpv1, slc17a7, unc5c, rbfox3, nefm, nefh
Stria vascularis
ryr2, col4a6, trpv1, nefh, calca, adcyap1, car12, slc17a7, panx3, gjb61
CompBio Enrichment Score (log2)

Figure S.11

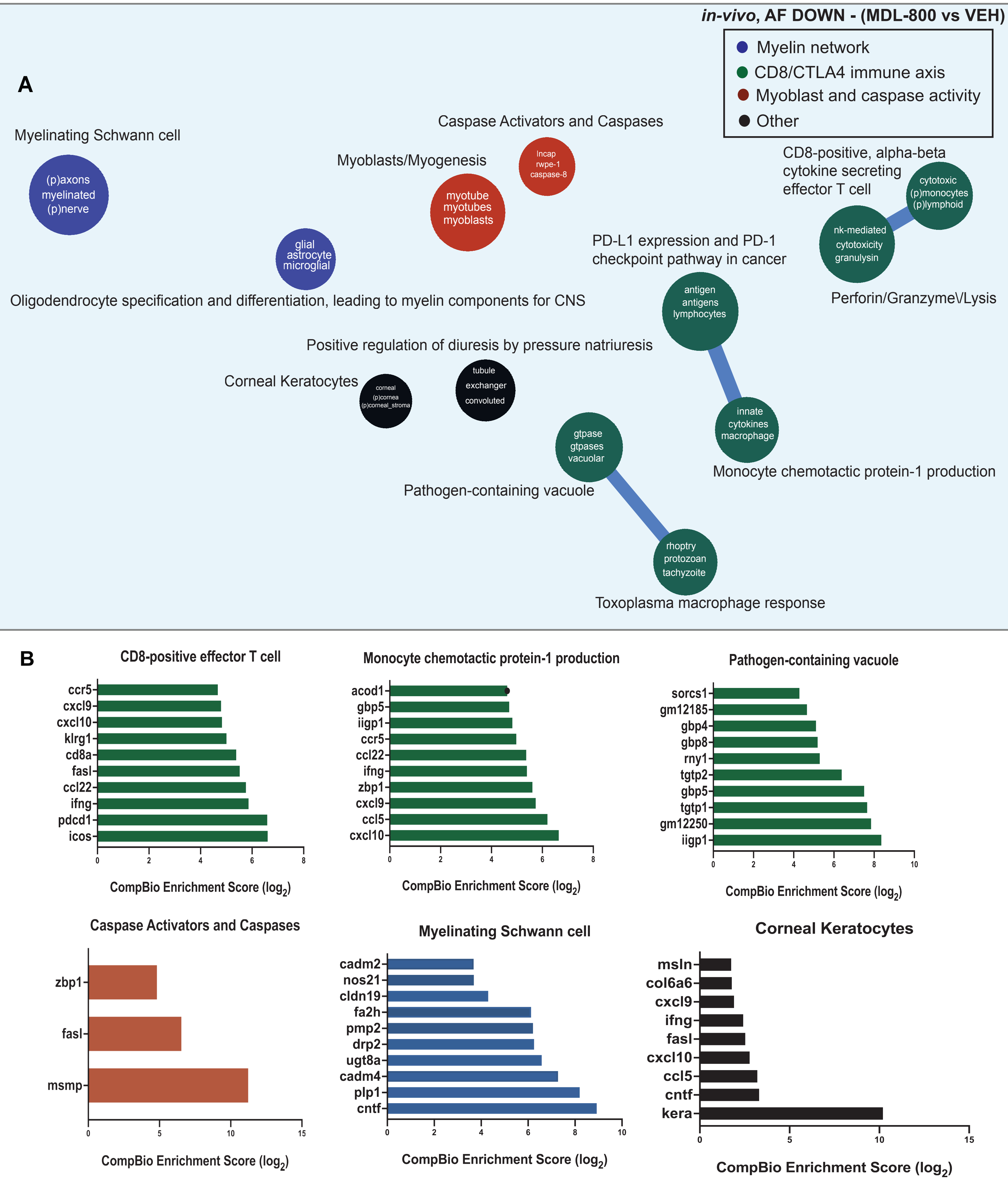

in-vivo, AF DOWN - (MDL-800 vs VEH)
Myelin network
CD8/CTLA4 immune axis
Myoblast and caspase activity
Other
A
Myelinating Schwann cell
(p)axons myelinated (p)nerve
Caspase Activators and Caspases
lncap rwpe-1 caspase-8
Myoblasts/Myogenesis
myotube myotubes myoblasts
glial astrocyte microglial
Oligodendrocyte specification and differentiation, leading to myelin components for CNS
CD8-positive, alpha-beta cytokine secreting effector T cell
cytotoxic (p)monocytes (p)lymphoid
nk-mediated cytotoxicity granulysin
Perforin/GranzymeV/Lysis
PD-L1 expression and PD-1 checkpoint pathway in cancer
antigen antigens lymphocytes
Positive regulation of diuresis by pressure natriuresis
tubule exchanger convoluted
Corneal Keratocytes
corneal (p)cornea (p)corneal_stroma
innate cytokines macrophage
Monocyte chemotactic protein-1 production
gtpase gtpases vacuolar
Pathogen-containing vacuole
rhoptry protozoan tachyzoite
Toxoplasma macrophage response
B
CD8-positive effector T cell
ccr5
cxcl9
cxcl10
klrg1
cd8a
fasl
ccl22
ifng
pdcd1
icos
CompBio Enrichment Score (log2)
Monocyte chemotactic protein-1 production
acod1
gbp5
iigp1
ccr5
ccl22
ifng
zbp1
cxcl9
ccl5
cxcl10
CompBio Enrichment Score (log2)
Pathogen-containing vacuole
sorcs1
gm12185
gbp4
gbp8
rny1
tgtp2
gbp5
tgtp1
gm12250
iigp1
CompBio Enrichment Score (log2)
Caspase Activators and Caspases
zbp1
fasl
msmp
CompBio Enrichment Score (log2)
Myelinating Schwann cell
cadm2
nos21
cldn19
fa2h
pmp2
drp2
ugt8a
cadm4
plp1
cntf
CompBio Enrichment Score (log2)
Corneal Keratocytes
msln
col6a6
cxcl9
ifng
fasl
cxcl10
ccl5
cntf
kera
CompBio Enrichment Score (log2)

Figure S. 12

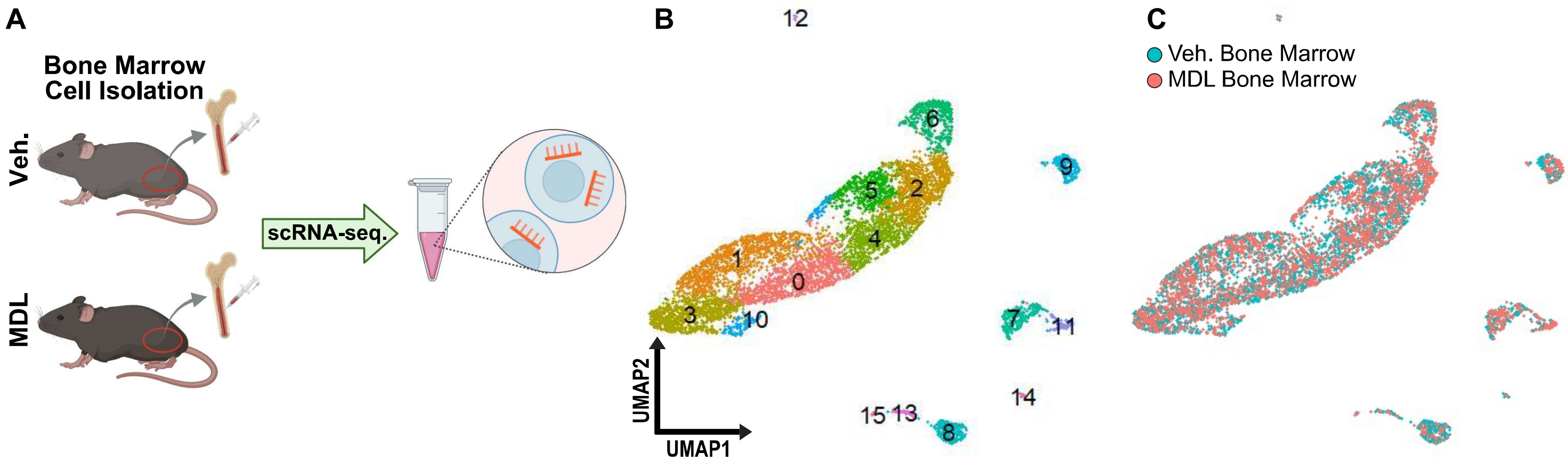

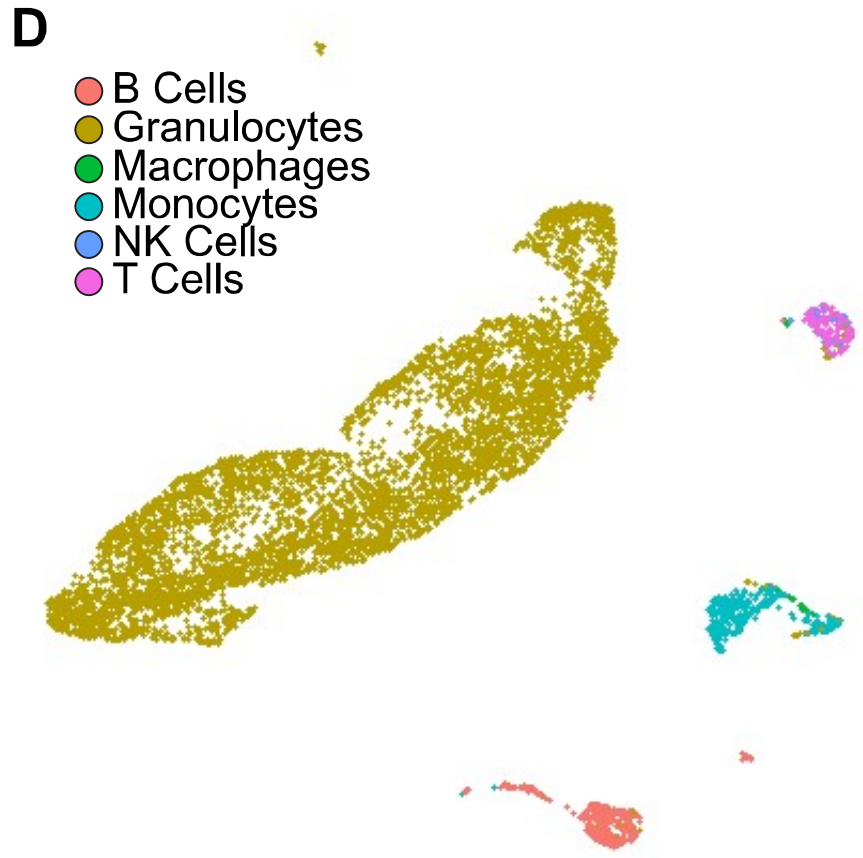

E

Annotation	Cell Count Veh.	Cell Count MDL	Percent Veh.	Percent MDL	OR	p-value
B Cells	279	76	7.69%	2.58%	0.32	5.6e-21
Granulocytes	3026	2616	83.45%	88.89%	1.59	2.6e-10
Macrophages	4	12	0.14%	0.41%	2.96	0.048
Monocytes	193	175	5.32%	5.95%	1.12	0.28
NK Cells	23	10	0.63%	0.34%	0.53	0.11
T Cells	100	54	2.76%	1.83%	0.66	0.014
Total	3626	2943	100%	100%		

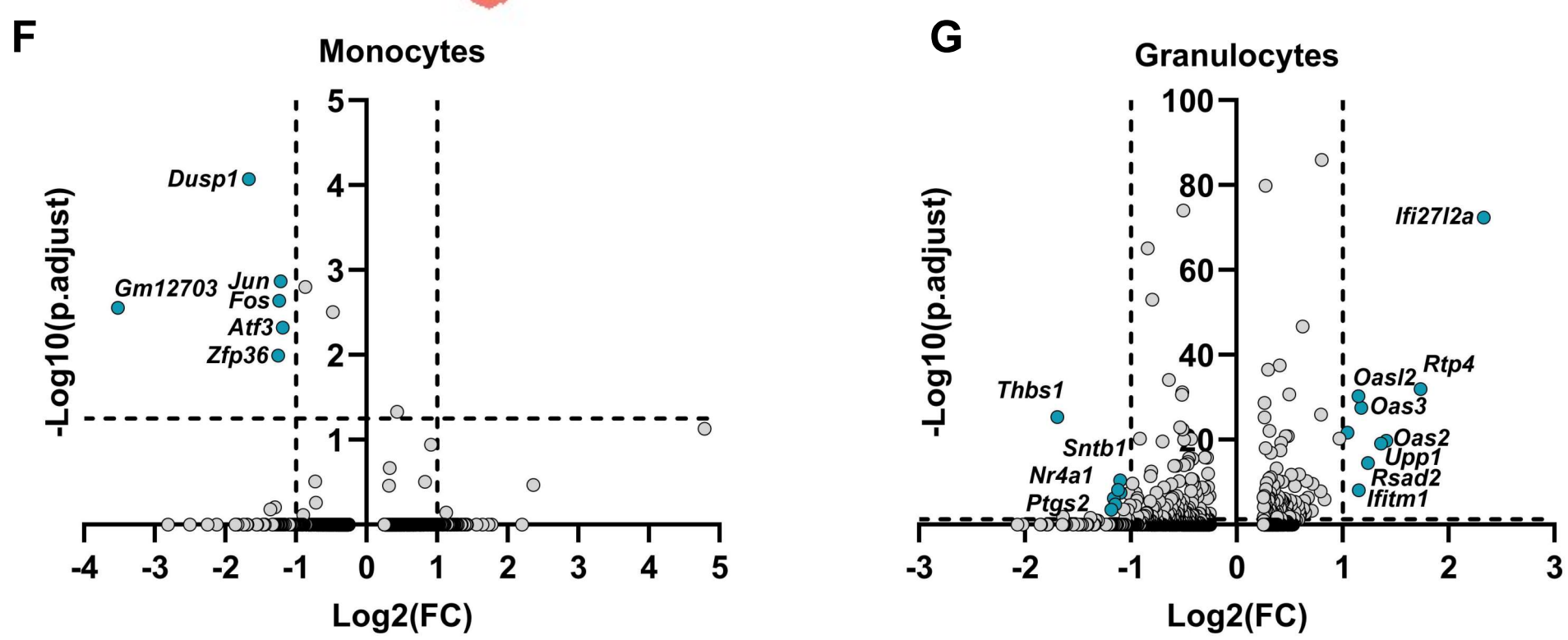

Figure S.13

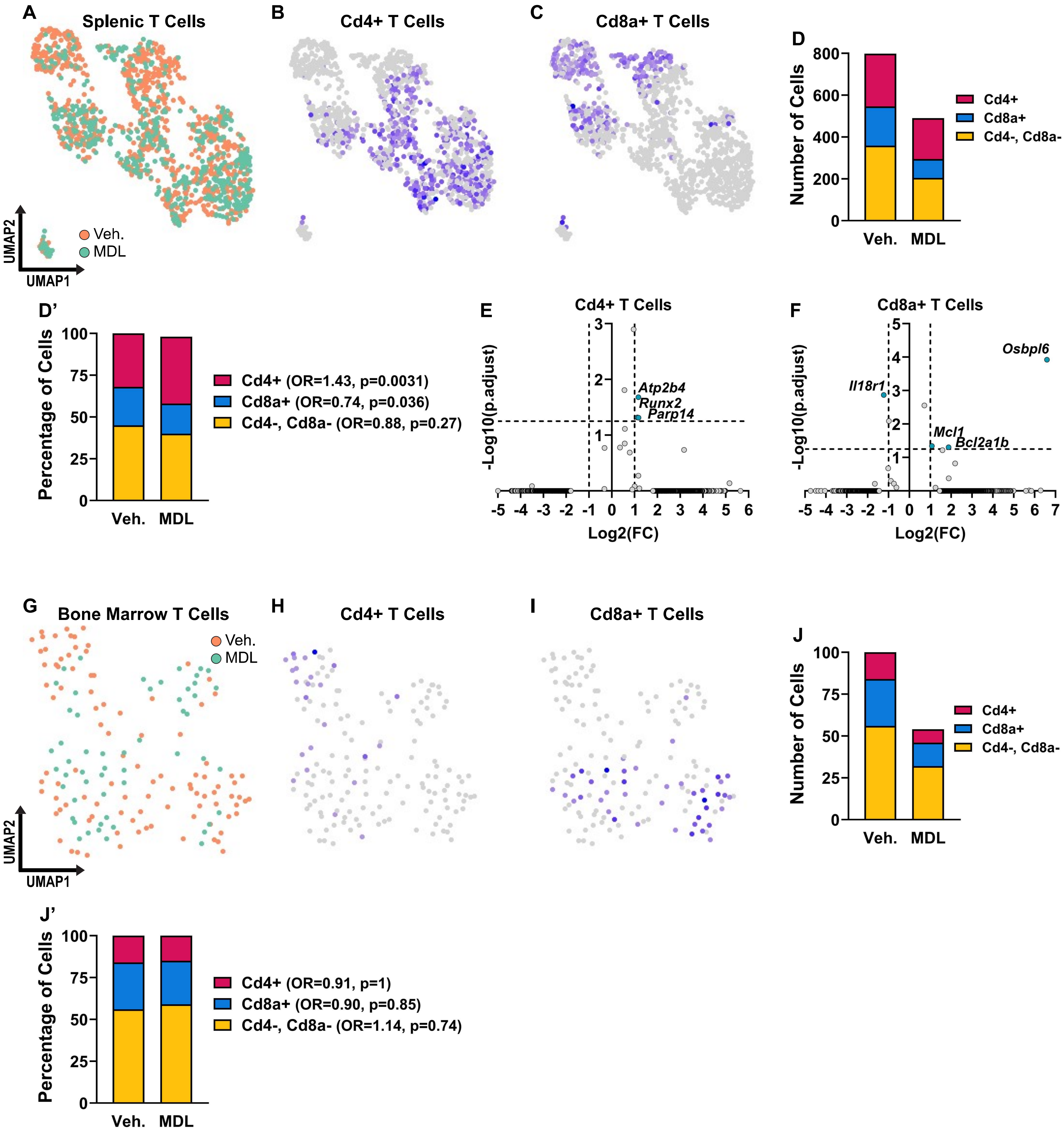
A
Splenic T Cells
Veh.
MDL
UMAP2
UMAP1
B
Cd4+ T Cells
C
Cd8a+ T Cells
D
Number of Cells
Veh.
MDL
Cd4+
Cd8a+
Cd4-, Cd8a-
D'
Percentage of Cells
Cd4+ (OR=1.43, p=0.0031)
Cd8a+ (OR=0.74, p=0.036)
Cd4-, Cd8a- (OR=0.88, p=0.27)
E
Cd4+ T Cells
-Log10(p.adjust)
Log2(FC)
Atp2b4
Runx2
Parp14
F
Cd8a+ T Cells
Osbpl6
Il18r1
Mcl1
Bcl2a1b
G
Bone Marrow T Cells
H
Cd4+ T Cells
I
Cd8a+ T Cells
J
Number of Cells
J'
Percentage of Cells
Cd4+ (OR=0.91, p=1)
Cd8a+ (OR=0.90, p=0.85)
Cd4-, Cd8a- (OR=1.14, p=0.74)

Figure S.14

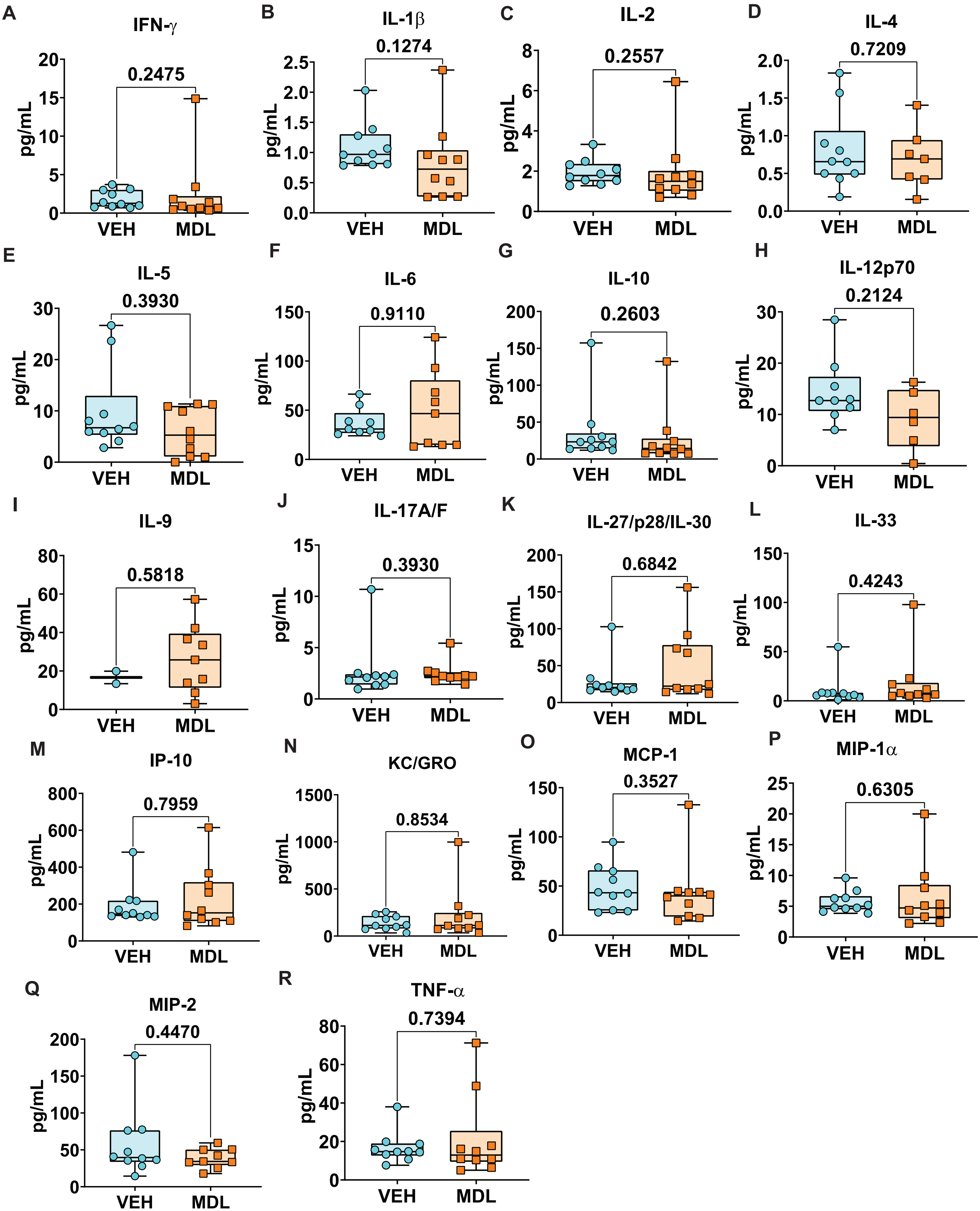
A
IFN-γ
0.2475
B
IL-1β
0.1274
C
IL-2
0.2557
D
IL-4
0.7209
E
IL-5
0.3930
F
IL-6
0.9110
G
IL-10
0.2603
H
IL-12p70
0.2124
I
IL-9
0.5818
J
IL-17A/F
0.3930
K
IL-27/p28/IL-30
0.6842
L
IL-33
0.4243
M
IP-10
0.7959
N
KC/GRO
0.8534
O
MCP-1
0.3527
P
MIP-1α
0.6305
Q
MIP-2
0.4470
R
TNF-α
0.7394
pg/mL
VEH
MDL

Figure S.15

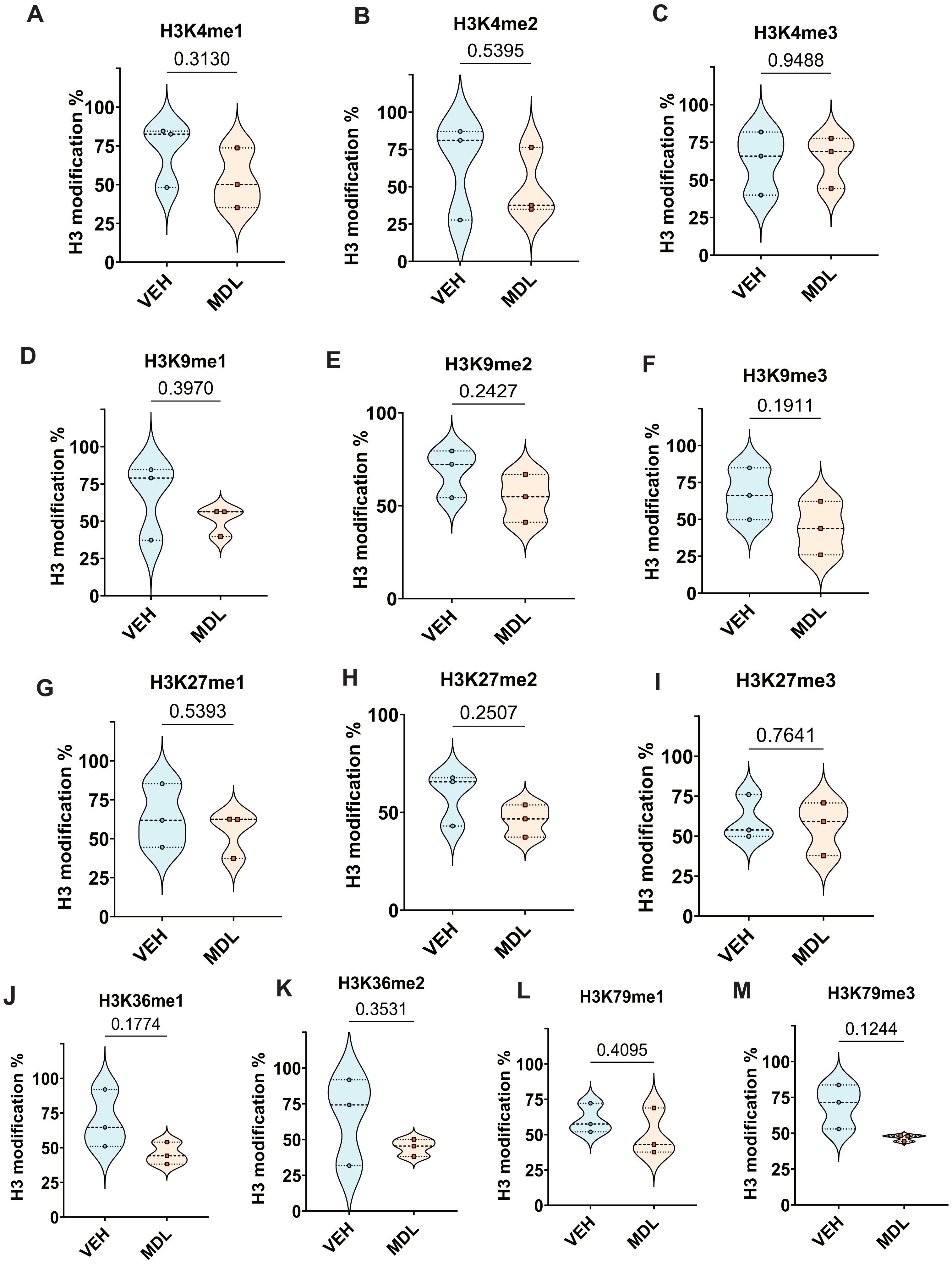

A
H3K4me1
0.3130
B
H3K4me2
0.5395
C
H3K4me3
0.9488
D
H3K9me1
0.3970
E
H3K9me2
0.2427
F
H3K9me3
0.1911
G
H3K27me1
0.5393
H
H3K27me2
0.2507
I
H3K27me3
0.7641
J
H3K36me1
0.1774
K
H3K36me2
0.3531
L
H3K79me1
0.4095
M
H3K79me3
0.1244
H3 modification %
0
25
50
75
100
VEH
MDL

Figure S.16

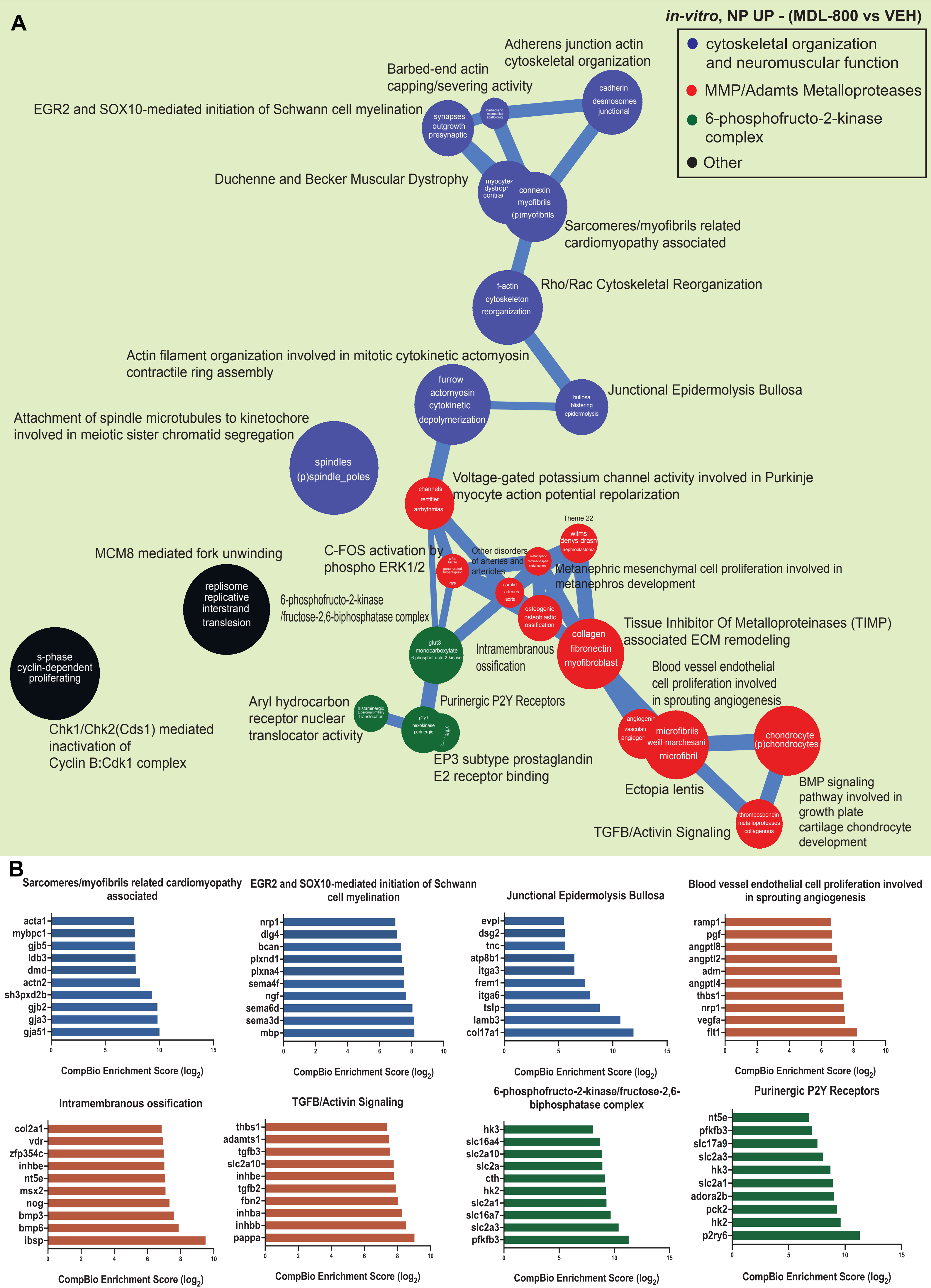
A
in-vitro, NP UP - (MDL-800 vs VEH)
cytoskeletal organization and neuromuscular function
MMP/Adamts Metalloproteases
6-phosphofructo-2-kinase complex
Other
Adherens junction actin cytoskeletal organization
Barbed-end actin capping/severing activity
EGR2 and SOX10-mediated initiation of Schwann cell myelination
cadherin desmosomes junctional
synapses outgrowth presynaptic
Duchenne and Becker Muscular Dystrophy
connexin myofibrils (p)myofibrils
Sarcomeres/myofibrils related cardiomyopathy associated
Rho/Rac Cytoskeletal Reorganization
f-actin cytoskeleton reorganization
Actin filament organization involved in mitotic cytokinetic actomyosin contractile ring assembly
furrow actomyosin cytokinetic depolymerization
Junctional Epidermolysis Bullosa
bullosa blistering epidermolysis
Attachment of spindle microtubules to kinetochore involved in meiotic sister chromatid segregation
spindles (p)spindle_poles
Voltage-gated potassium channel activity involved in Purkinje myocyte action potential repolarization
channels rectifier arrhythmias
Theme 22
wilms denys-drash nephroblastoma
MCM8 mediated fork unwinding
C-FOS activation by phospho ERK1/2
Other disorders of arteries and arterioles
Metanephric mesenchymal cell proliferation involved in metanephros development
replisome replicative interstrand translesion
6-phosphofructo-2-kinase /fructose-2,6-biphosphatase complex
carotid arteries aorta
osteogenic osteoblastic ossification
Tissue Inhibitor Of Metalloproteinases (TIMP) associated ECM remodeling
collagen fibronectin myofibroblast
s-phase cyclin-dependent proliferating
glut3 monocarboxylate 6-phosphofructo-2-kinase
Intramembranous ossification
Blood vessel endothelial cell proliferation involved in sprouting angiogenesis
Aryl hydrocarbon receptor nuclear translocator activity
Purinergic P2Y Receptors
Chk1/Chk2(Cds1) mediated inactivation of Cyclin B:Cdk1 complex
p2y1 hexokinase purinergic
microfibrils weill-marchesani microfibril
chondrocyte (p)chondrocytes
EP3 subtype prostaglandin E2 receptor binding
Ectopia lentis
BMP signaling pathway involved in growth plate cartilage chondrocyte development
TGFB/Activin Signaling
thrombospondin metalloproteases collagenous
B
Sarcomeres/myofibrils related cardiomyopathy associated
acta1
mybpc1
gjb5
ldb3
dmd
actn2
sh3pxd2b
gjb2
gja3
gja51
CompBio Enrichment Score (log2)
EGR2 and SOX10-mediated initiation of Schwann cell myelination
nrp1
dlg4
bcan
plxnd1
plxna4
sema4f
ngf
sema6d
sema3d
mbp
CompBio Enrichment Score (log2)
Junctional Epidermolysis Bullosa
evpl
dsg2
tnc
atp8b1
itga3
frem1
itga6
tslp
lamb3
col17a1
CompBio Enrichment Score (log2)
Blood vessel endothelial cell proliferation involved in sprouting angiogenesis
ramp1
pgf
angptl8
angptl2
adm
angptl4
thbs1
nrp1
vegfa
flt1
CompBio Enrichment Score (log2)
Intramembranous ossification
col2a1
vdr
zfp354c
inhbe
nt5e
msx2
nog
bmp3
bmp6
ibsp
CompBio Enrichment Score (log2)
TGFB/Activin Signaling
thbs1
adamts1
tgfb3
slc2a10
inhbe
tgfb2
fbn2
inhba
inhbb
pappa
CompBio Enrichment Score (log2)
6-phosphofructo-2-kinase/fructose-2,6-biphosphatase complex
hk3
slc16a4
slc2a10
slc2a
cth
hk2
slc2a1
slc16a7
slc2a3
pfkfb3
CompBio Enrichment Score (log2)
Purinergic P2Y Receptors
nt5e
pfkfb3
slc17a9
slc2a3
hk3
slc2a1
adora2b
pck2
hk2
p2ry6
CompBio Enrichment Score (log2)

Figure S.17

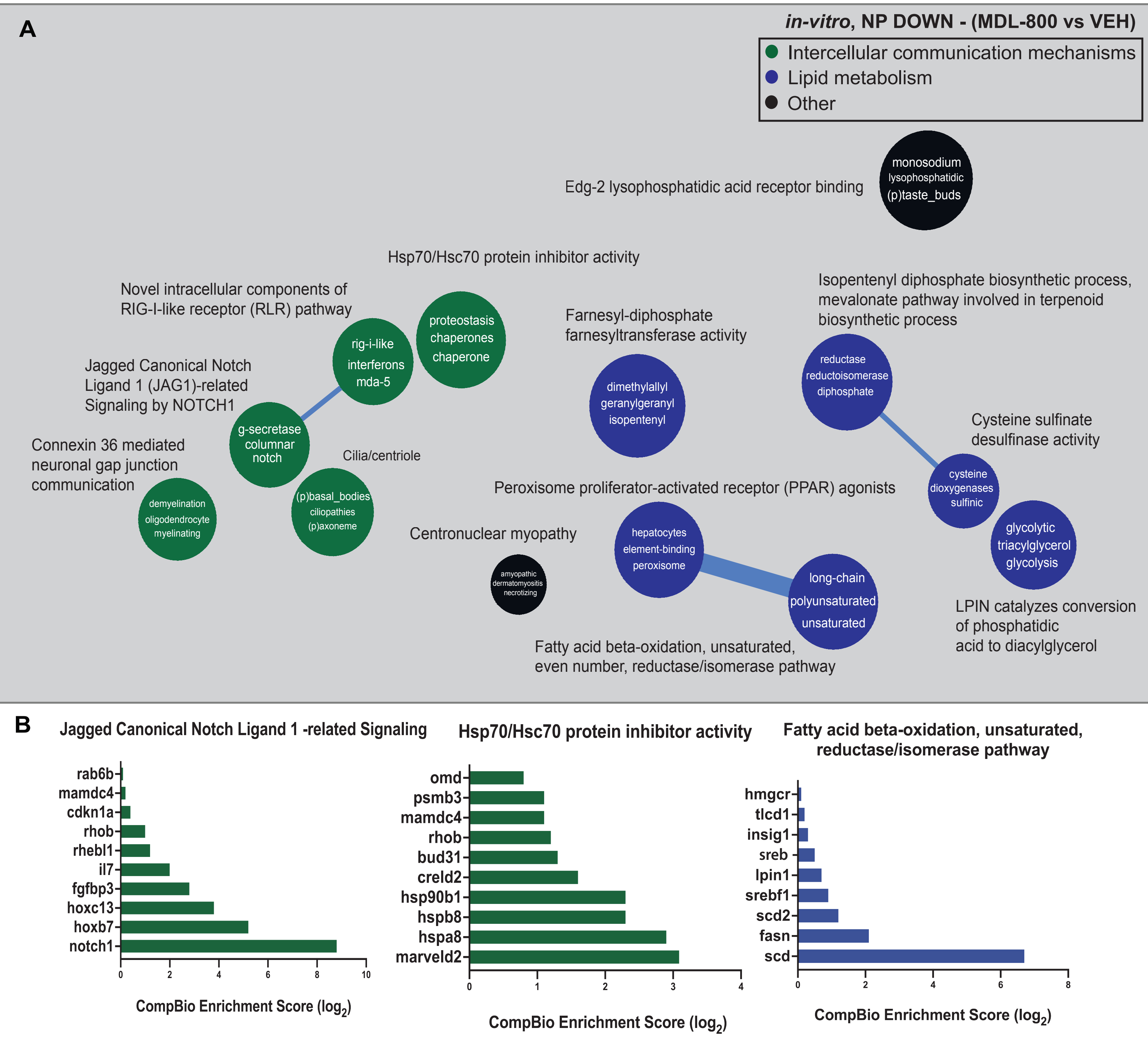

A
in-vitro, NP DOWN - (MDL-800 vs VEH)
Intercellular communication mechanisms
Lipid metabolism
Other
Edg-2 lysophosphatidic acid receptor binding
monosodium lysophosphatidic (p)taste_buds
Hsp70/Hsc70 protein inhibitor activity
Novel intracellular components of RIG-I-like receptor (RLR) pathway
proteostasis chaperones chaperone
rig-i-like interferons mda-5
Jagged Canonical Notch Ligand 1 (JAG1)-related Signaling by NOTCH1
g-secretase columnar notch
Connexin 36 mediated neuronal gap junction communication
demyelination oligodendrocyte myelinating
Cilia/centriole
(p)basal_bodies ciliopathies (p)axoneme
Farnesyl-diphosphate farnesyltransferase activity
dimethylallyl geranylgeranyl isopentenyl
Isopentenyl diphosphate biosynthetic process, mevalonate pathway involved in terpenoid biosynthetic process
reductase reductoisomerase diphosphate
Cysteine sulfinate desulfinase activity
cysteine dioxygenases sulfinic
Peroxisome proliferator-activated receptor (PPAR) agonists
hepatocytes element-binding peroxisome
Centronuclear myopathy
amyopathic dermatomyositis necrotizing
long-chain polyunsaturated unsaturated
glycolytic triacylglycerol glycolysis
LPIN catalyzes conversion of phosphatidic acid to diacylglycerol
Fatty acid beta-oxidation, unsaturated, even number, reductase/isomerase pathway
B
Jagged Canonical Notch Ligand 1 -related Signaling
rab6b
mamdc4
cdkn1a
rhob
rhebl1
il7
fgfbp3
hoxc13
hoxb7
notch1
0 2 4 6 8 10
CompBio Enrichment Score (log2)
Hsp70/Hsc70 protein inhibitor activity
omd
psmb3
mamdc4
rhob
bud31
creld2
hsp90b1
hspb8
hspa8
marveld2
0 1 2 3 4
CompBio Enrichment Score (log2)
Fatty acid beta-oxidation, unsaturated, reductase/isomerase pathway
hmgcr
tlcd1
insig1
sreb
lpin1
srebf1
scd2
fasn
scd
0 2 4 6 8
CompBio Enrichment Score (log2)

Figure S.18

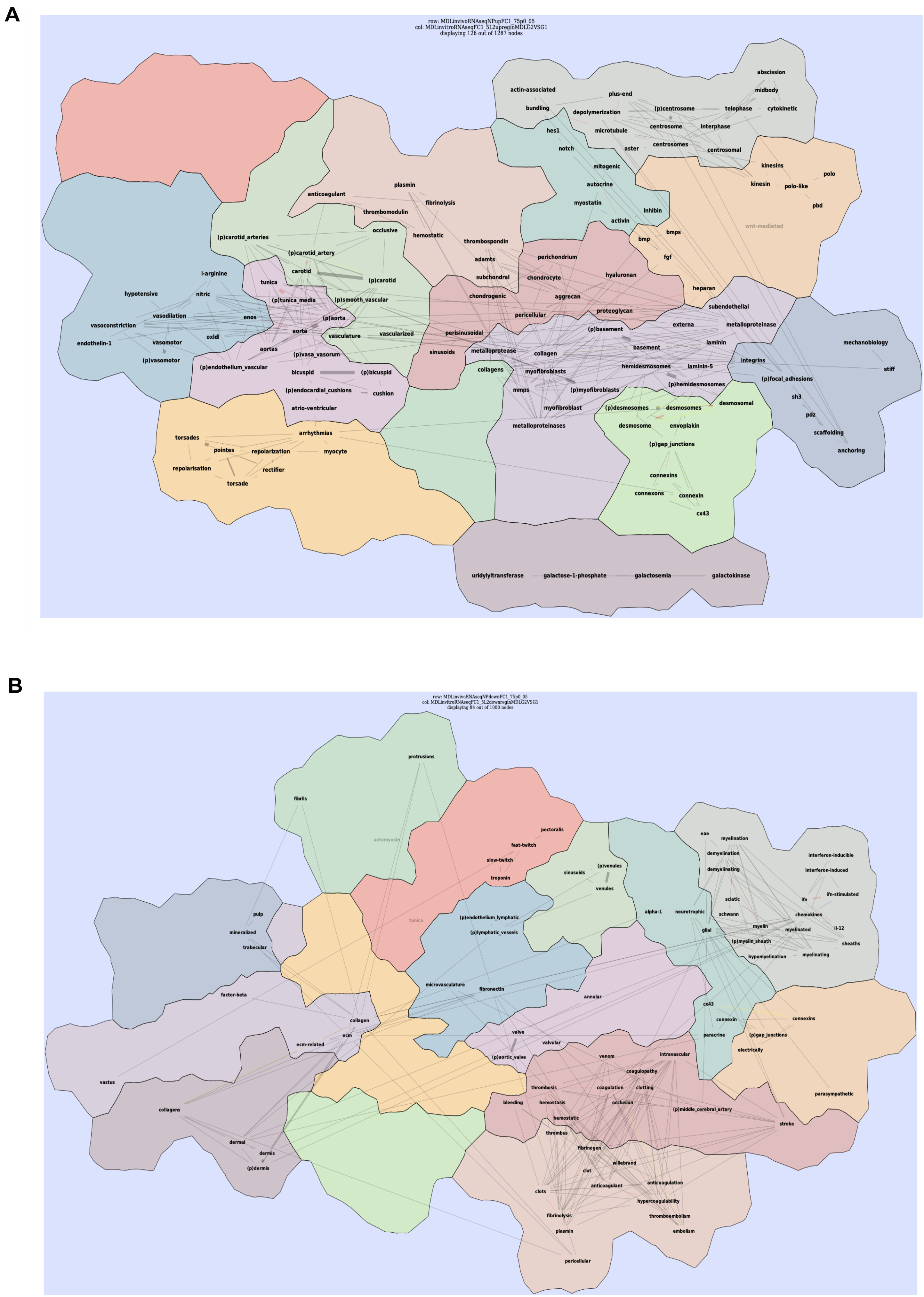
A
row: MDLinvivoRNAseqNPupFC1_75p0_05
col: MDLinvitroRNAseqFC1_5L2upreginMDLG2VSG1
displaying 126 out of 1287 nodes
actin-associated
plus-end
abscission
midbody
bundling
depolymerization
(p)centrosome
telophase
cytokinetic
hes1
microtubule
centrosome
interphase
notch
aster
centrosomes
centrosomal
mitogenic
kinesins
polo
autocrine
kinesin
polo-like
myostatin
pbd
inhibin
activin
wnt-mediated
bmps
bmp
fgf
plasmin
anticoagulant
fibrinolysis
thrombomodulin
occlusive
hemostatic
(p)carotid_arteries
thrombospondin
(p)carotid_artery
perichondrium
adamts
carotid
(p)carotid
subchondral
chondrocyte
hyaluronan
l-arginine
tunica
heparan
hypotensive
nitric
(p)tunica_media
(p)smooth_vascular
chondrogenic
aggrecan
subendothelial
vasodilation
enos
(p)aorta
pericellular
proteoglycan
vasoconstriction
aorta
externa
metalloproteinase
oxldl
vasculature
vascularized
perisinusoidal
(p)basement
endothelin-1
vasomotor
aortas
sinusoids
metalloprotease
collagen
basement
laminin
mechanobiology
(p)vasomotor
(p)vasa_vasorum
integrins
(p)endothelium_vascular
collagens
hemidesmosomes
laminin-5
stiff
bicuspid
(p)bicuspid
myofibroblasts
(p)focal_adhesions
(p)hemidesmosomes
(p)endocardial_cushions
cushion
mmps
(p)myofibroblasts
sh3
desmosomal
atrio-ventricular
myofibroblast
(p)desmosomes
desmosomes
pdz
metalloproteinases
desmosome
envoplakin
scaffolding
arrhythmias
torsades
(p)gap_junctions
anchoring
pointes
repolarization
myocyte
repolarisation
rectifier
connexins
torsade
connexons
connexin
cx43
uridylyltransferase
galactose-1-phosphate
galactosemia
galactokinase
B
row: MDLinvivoRNAseqNPdownFC1_75p0_05
col: MDLinvitroRNAseqFC1_5L2downreginMDLG2VSG1
displaying 84 out of 1000 nodes
protrusions
fibrils
pectoralis
eae
myelination
actomyosin
fast-twitch
demyelination
interferon-inducible
slow-twitch
(p)venules
demyelinating
interferon-induced
sinusoids
troponin
venules
ifn
ifn-stimulated
sciatic
pulp
alpha-1
neurotrophic
schwann
chemokines
tunica
(p)endothelium_lymphatic
myelin
il-12
mineralized
(p)lymphatic_vessels
glial
myelinated
(p)myelin_sheath
sheaths
trabecular
hypomyelination
myelinating
microvasculature
fibronectin
factor-beta
annular
cx43
collagen
connexin
connexins
ecm
valve
paracrine
(p)gap_junctions
ecm-related
valvular
electrically
(p)aortic_valve
venom
intravascular
vastus
coagulopathy
thrombosis
coagulation
clotting
parasympathetic
collagens
bleeding
hemostasis
occlusion
(p)middle_cerebral_artery
hemostatic
stroke
thrombus
dermal
fibrinogen
dermis
willebrand
(p)dermis
clot
anticoagulant
anticoagulation
clots
hypercoagulability
fibrinolysis
thromboembolism
plasmin
embolism
pericellular

Figure S.19

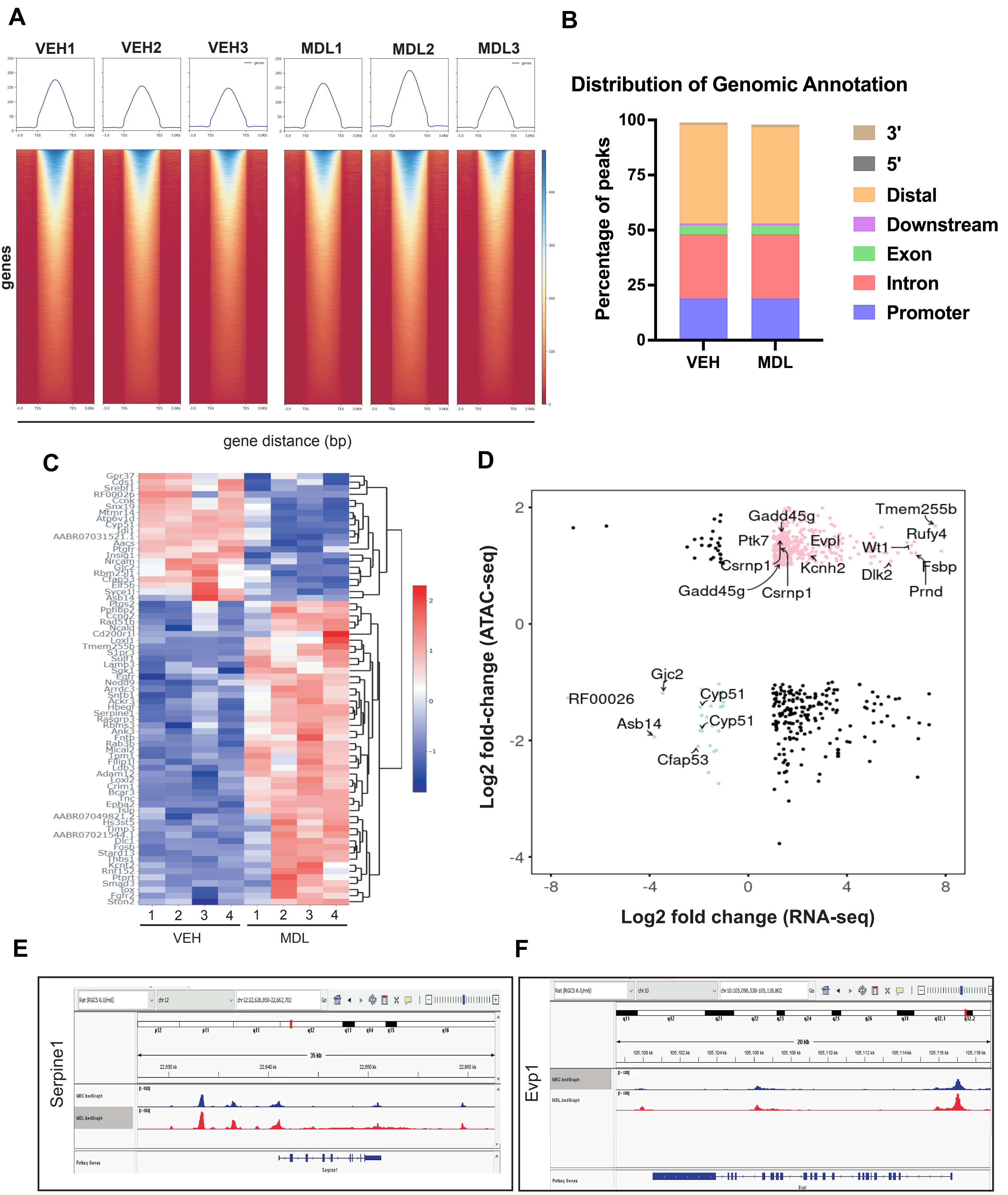
A
VEH1
VEH2
VEH3
MDL1
MDL2
MDL3
genes
gene distance (bp)
B
Distribution of Genomic Annotation
Percentage of peaks
VEH
MDL
3'
5'
Distal
Downstream
Exon
Intron
Promoter
C
VEH
MDL
D
Log2 fold-change (ATAC-seq)
Log2 fold change (RNA-seq)
Gadd45g
Ptk7
Evpl
Csrnp1
Kcnh2
Tmem255b
Rufy4
Wt1
Dlk2
Fsbp
Prnd
Gjc2
RF00026
Cyp51
Asb14
Cfap53
E
Serpine1
F
Evp1

Figure S.20

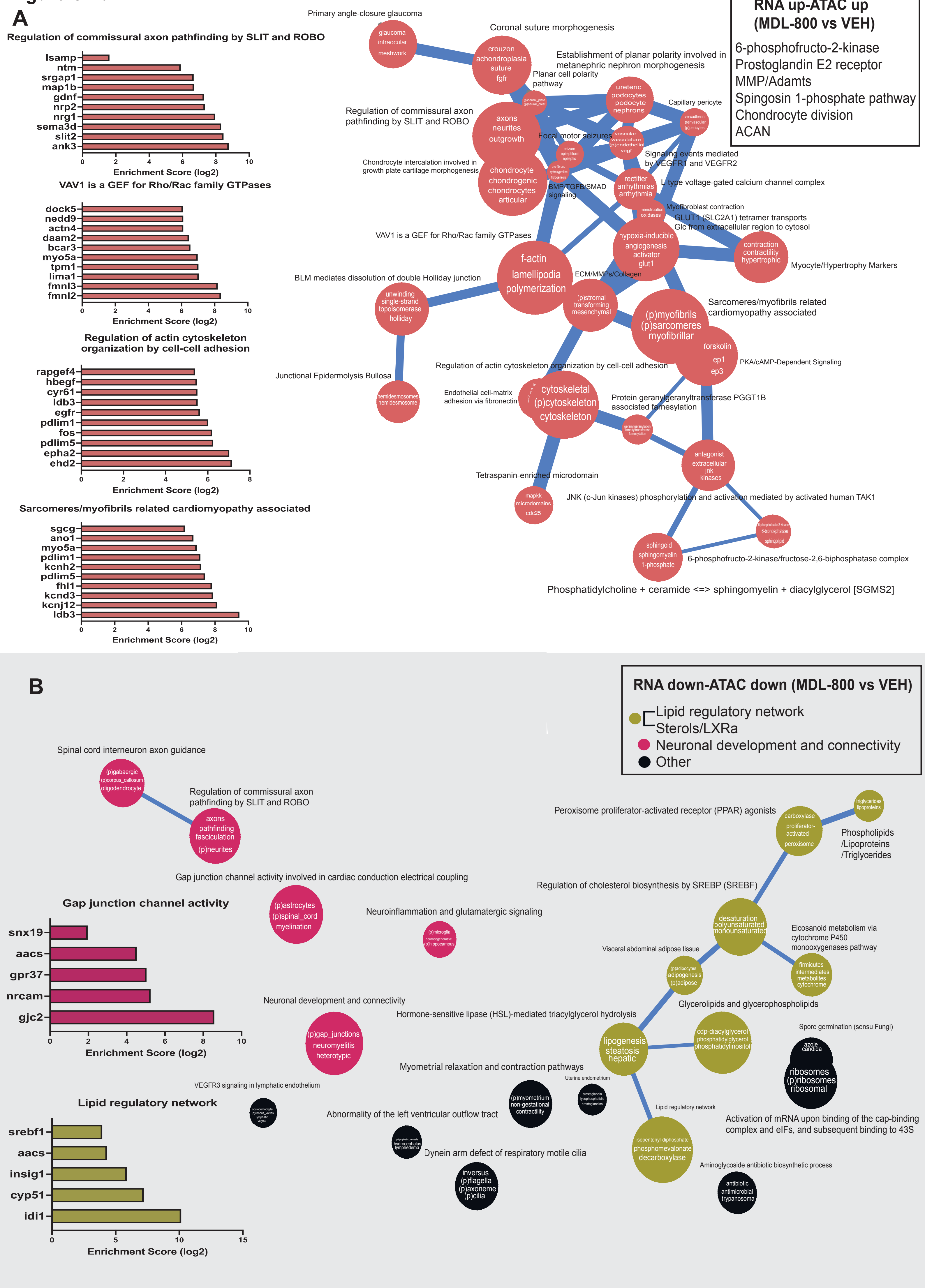

A
Regulation of commissural axon pathfinding by SLIT and ROBO
lsamp
ntm
srgap1
map1b
gdnf
nrp2
nrg1
sema3d
slit2
ank3
Enrichment Score (log2)
VAV1 is a GEF for Rho/Rac family GTPases
dock5
nedd9
actn4
daam2
bcar3
myo5a
tpm1
lima1
fmnl3
fmnl2
Enrichment Score (log2)
Regulation of actin cytoskeleton organization by cell-cell adhesion
rapgef4
hbegf
cyr61
ldb3
egfr
pdlim1
fos
pdlim5
epha2
ehd2
Enrichment Score (log2)
Sarcomeres/myofibrils related cardiomyopathy associated
sgcg
ano1
myo5a
pdlim1
kcnh2
pdlim5
fhl1
kcnd3
kcnj12
ldb3
Enrichment Score (log2)
RNA up-ATAC up (MDL-800 vs VEH)
6-phosphofructo-2-kinase
Prostoglandin E2 receptor
MMP/Adamts
Spingosin 1-phosphate pathway
Chondrocyte division
ACAN
Primary angle-closure glaucoma
glaucoma intraocular meshwork
Coronal suture morphogenesis
crouzon achondroplasia suture fgfr
Establishment of planar polarity involved in metanephric nephron morphogenesis
Planar cell polarity pathway
ureteric podocytes podocyte nephrons
Capillary pericyte
Regulation of commissural axon pathfinding by SLIT and ROBO
axons neurites outgrowth
Focal motor seizures
vascular vasculature (p)endothelial vegf
Signaling events mediated by VEGFR1 and VEGFR2
Chondrocyte intercalation involved in growth plate cartilage morphogenesis
chondrocyte chondrogenic chondrocytes articular
BMP/TGFB/SMAD signaling
rectifier arrhythmias arrhythmia
L-type voltage-gated calcium channel complex
Myofibroblast contraction
GLUT1 (SLC2A1) tetramer transports Glc from extracellular region to cytosol
VAV1 is a GEF for Rho/Rac family GTPases
hypoxia-inducible angiogenesis activator glut1
contraction contractility hypertrophic
Myocyte/Hypertrophy Markers
BLM mediates dissolution of double Holliday junction
f-actin lamellipodia polymerization
ECM/MMPs/Collagen
unwinding single-strand topoisomerase holliday
(p)stromal transforming mesenchymal
Sarcomeres/myofibrils related cardiomyopathy associated
(p)myofibrils (p)sarcomeres myofibrillar
forskolin ep1 ep3
PKA/cAMP-Dependent Signaling
Junctional Epidermolysis Bullosa
Regulation of actin cytoskeleton organization by cell-cell adhesion
hemidesmosomes hemidesmosome
Endothelial cell-matrix adhesion via fibronectin
cytoskeletal (p)cytoskeleton cytoskeleton
Protein geranylgeranyltransferase PGGT1B associated farnesylation
antagonist extracellular jnk kinases
Tetraspanin-enriched microdomain
mapkk microdomains cdc25
JNK (c-Jun kinases) phosphorylation and activation mediated by activated human TAK1
sphingoid sphingomyelin 1-phosphate
6-phosphofructo-2-kinase/fructose-2,6-biphosphatase complex
Phosphatidylcholine + ceramide <=> sphingomyelin + diacylglycerol [SGMS2]
B
RNA down-ATAC down (MDL-800 vs VEH)
Lipid regulatory network
Sterols/LXRa
Neuronal development and connectivity
Other
Spinal cord interneuron axon guidance
(p)gabaergic (p)corpus_callosum oligodendrocyte
Regulation of commissural axon pathfinding by SLIT and ROBO
axons pathfinding fasciculation (p)neurites
Gap junction channel activity involved in cardiac conduction electrical coupling
(p)astrocytes (p)spinal_cord myelination
Gap junction channel activity
snx19
aacs
gpr37
nrcam
gjc2
Enrichment Score (log2)
Neuroinflammation and glutamatergic signaling
Neuronal development and connectivity
(p)gap_junctions neuromyelitis heterotypic
VEGFR3 signaling in lymphatic endothelium
Lipid regulatory network
srebf1
aacs
insig1
cyp51
idi1
Enrichment Score (log2)
Peroxisome proliferator-activated receptor (PPAR) agonists
carboxylase proliferator-activated peroxisome
triglycerides lipoproteins
Phospholipids /Lipoproteins /Triglycerides
Regulation of cholesterol biosynthesis by SREBP (SREBF)
desaturation polyunsaturated monounsaturated
Eicosanoid metabolism via cytochrome P450 monooxygenases pathway
Visceral abdominal adipose tissue
(p)adipocytes adipogenesis (p)adipose
firmicutes intermediates metabolites cytochrome
Glycerolipids and glycerophospholipids
Hormone-sensitive lipase (HSL)-mediated triacylglycerol hydrolysis
lipogenesis steatosis hepatic
cdp-diacylglycerol phosphatidylglycerol phosphatidylinositol
Spore germination (sensu Fungi)
azole candida
ribosomes (p)ribosomes ribosomal
Myometrial relaxation and contraction pathways
Uterine endometrium
(p)myometrium non-gestational contractility
Lipid regulatory network
Abnormality of the left ventricular outflow tract
Activation of mRNA upon binding of the cap-binding complex and eIFs, and subsequent binding to 43S
hydrocephalus lymphedema
Dynein arm defect of respiratory motile cilia
isopentenyl-diphosphate phosphomevalonate decarboxylase
Aminoglycoside antibiotic biosynthetic process
inversus (p)flagella (p)axoneme (p)cilia
antibiotic antimicrobial trypanosoma

Figure S.21

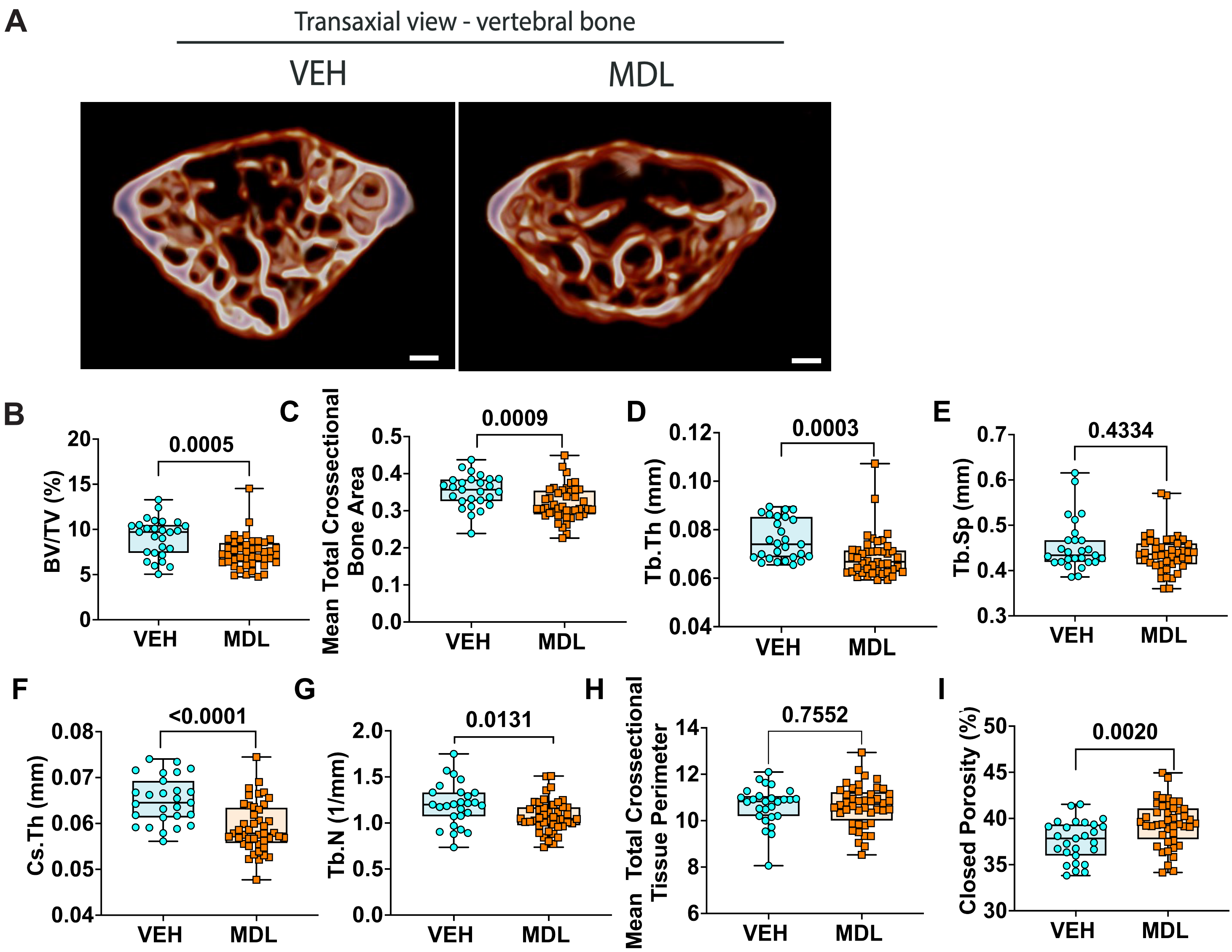

A
Transaxial view - vertebral bone
VEH
MDL
B
BV/TV (%)
0.0005
VEH
MDL
C
Mean Total Crossectional Bone Area
0.0009
D
Tb.Th (mm)
0.0003
E
Tb.Sp (mm)
0.4334
F
Cs.Th (mm)
<0.0001
G
Tb.N (1/mm)
0.0131
H
Mean Total Crossectional Tissue Perimeter
0.7552
I
Closed Porosity (%)
0.0020

Figure S.22

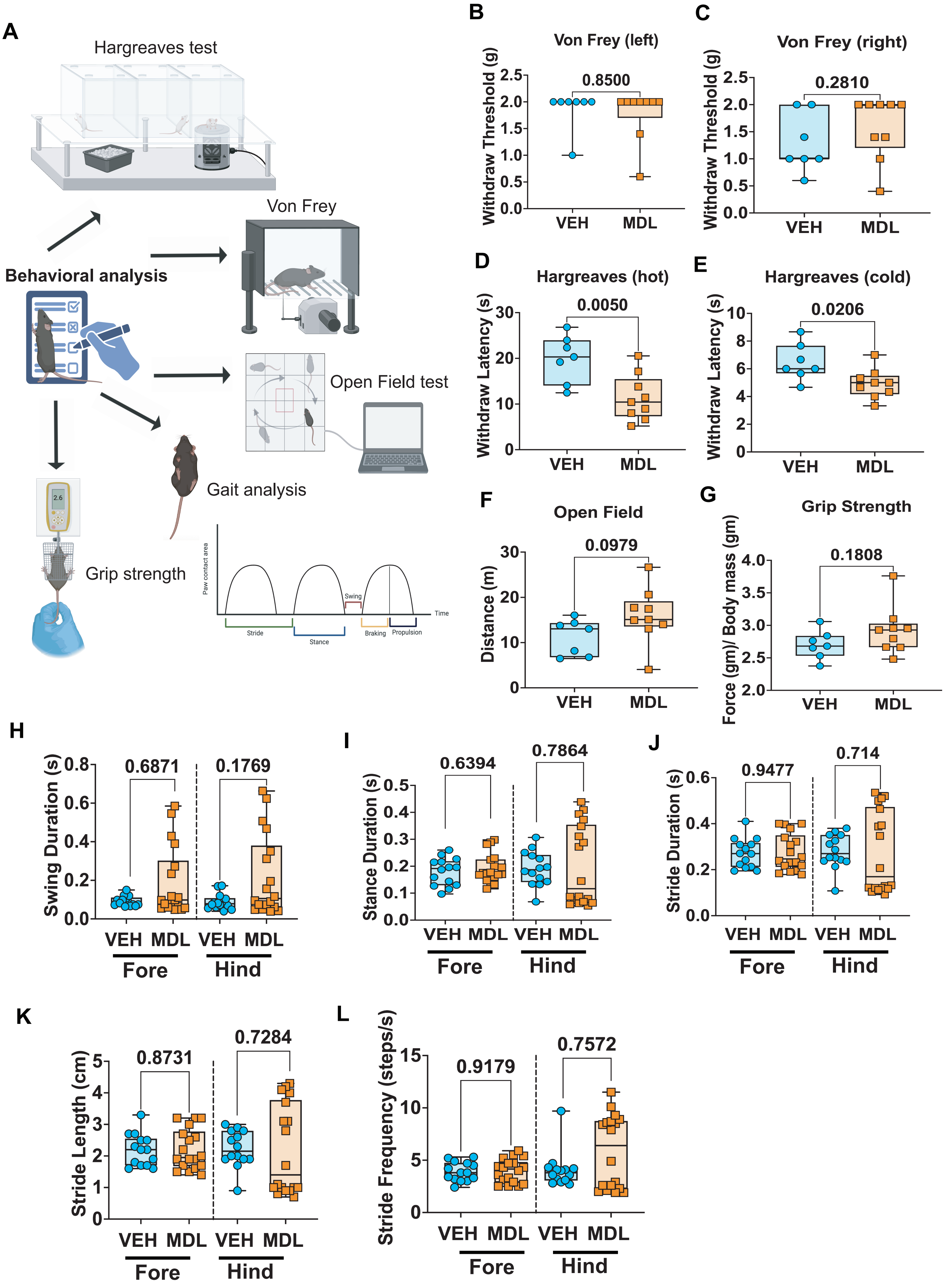

A
Hargreaves test
Von Frey
Behavioral analysis
Open Field test
Gait analysis
Grip strength
2.6
Paw contact area
Swing
Time
Stride
Stance
Braking
Propulsion
B
Von Frey (left)
Withdraw Threshold (g)
0.8500
VEH
MDL
C
Von Frey (right)
Withdraw Threshold (g)
0.2810
VEH
MDL
D
Hargreaves (hot)
Withdraw Latency (s)
0.0050
VEH
MDL
E
Hargreaves (cold)
Withdraw Latency (s)
0.0206
VEH
MDL
F
Open Field
Distance (m)
0.0979
VEH
MDL
G
Grip Strength
Force (gm)/ Body mass (gm)
0.1808
VEH
MDL
H
Swing Duration (s)
0.6871
0.1769
VEH MDL VEH MDL
Fore
Hind
I
Stance Duration (s)
0.6394
0.7864
VEH MDL VEH MDL
Fore
Hind
J
Stride Duration (s)
0.9477
0.714
VEH MDL VEH MDL
Fore
Hind
K
Stride Length (cm)
0.8731
0.7284
VEH MDL VEH MDL
Fore
Hind
L
Stride Frequency (steps/s)
0.9179
0.7572
VEH MDL VEH MDL
Fore
Hind
